# Synthesis, antimicrobial and antioxidant activity of triazole, pyrazole containing thiazole derivatives and molecular docking studies on COVID-19

**DOI:** 10.1186/s13065-023-00965-8

**Published:** 2023-06-17

**Authors:** Raghavender Matta, Jalapathi Pochampally, Bala Narsimha Dhoddi, Shankar Bhookya, Sampath Bitla, Anjini Gayatri Akkiraju

**Affiliations:** 1 Department of Chemistry, Osmania University, Hyderabad, 500007 India; 2 Department of Chemistry, Sreenidhi University, Hyderabad, 501301 India; 3 Molecular Medicine Lab, Department of Genetics & Biotechnology, Osmania University, Hyderabad, 500007 India

**Keywords:** 1, 2, 3-triazole, Pyrazole, Thiazole, Antimicrobial activity, Antioxidant, Molecular docking

## Abstract

New series of biologically active triazole and pyrazole compounds containing 2, 4-disubstituted thiazole analogues (12a-l) were synthesized from p-hydroxy benzaldehyde and phenyl hydrazine in excellent yields and purity. All the synthesized compounds were unambiguously identified based on their spectral data analyses (IR, ^1^H-NMR, ^13^C-NMR spectra, and HRMS). The final derivatives were evaluated for their in vitro anti-microbial activity after thorough purification. Among all the tested compounds, the compound **12e**, **12f** and **12 k** possess the highest growth inhibitory activity at MIC values of **4.8, 5.1** and **4.0** μg/ml respectively. The antioxidant properties of these compounds demonstrated and revealed remarkable activity compared to the standard antioxidant by using the DPPH free radical-scavenging assay. Moreover, molecular docking studies to evaluate the probable interactions with the catalytic domain of the gram-positive *S. aureus* topoisomerase IV enzyme may provide new insights for developing these new hybrids as potential antimicrobial agents. The binding affinities of compounds **12a-l** were ranging from − 10.0 to − 11.0 kcal/mol with topoisomerase IV enzyme and with COVID-19 main protease binding affinities are ranging from − 8.2 to − 9.3 kcal/mol. These docking studies reveal that the compounds **12a-l** could be the best inhibitors for the novel SARS Cov-2 virus and have more future in discovery of potent drug candidates.

**Supplementary Information:**

The online version contains supplementary material available at 10.1186/s13065-023-00965-8.

## Introduction

The 1,2,3-triazoles are renowned scaffolds that are simple to conjugate with additional heterocyclic groups. As a result, a variety of bioactive compounds with antibacterial [1, 2], anticancer [3] and anti-hypercholesterolemic [4, 5] actions have begun to target these triazole conjugated structural motifs as a common pharmacological target. On the other hand, compounds containing pyrazole scaffolds, with π-excessive monocyclic aromatic heterocyclic groups, are prone to possess an extensive biological activities like antimicrobial [6, 7], antimalarial [8, 9], anti-inflammatory [10, 11], antiviral [12, 13], antileshmanial [14–16], antiproliferative [17, 18], anticancer activities [19]. Pyrazole-based prominent drugs are available on the market, such as Pyrazofurin, Encorafenib, Celecoxib, Crizotinib, Lonazolac, etc. [20–23].

Thiazole is a specific moiety which plays a vital role in the biochemistry as a part of vitamin B1 structure. Thiazole moiety is always included in the preparation of pharmaceutical compounds containing flavourings, perfumes, and agrochemicals. Thiazole scaffold containing compounds viz*.* thiamine (B1) [24], sulfathiazole [25], abafungin (antifungal drug) [25], anti-inflammatory [26], diabetes [27], analgesic [28], cancer [29] possess a wide range of biological activities. Given these significant inhibitory activities, the new triazole and pyrazole chemical entities based 2,4-disubstituted thiazole hybrids are flexible moieties with constant attraction in research prospects and draw the attention of medicinal chemists due to their ease of synthetic feasibility, numerous biological activities when combined with other heterocyclic moieties [30–34]. Based on the literature, it is evident that the synthesis of biologically active compounds containing triazole, pyrazole, and thiazole moieties has a tremendously attractive individual interest among researchers because of their applications in medicinal chemistry, as cited above. However, heterocyclic building blocks consisting of triazole, pyrazole, and thiazole moieties in a group together have been reported in few numbers. Because of the superior biological activity and inspiration from the above-cited findings, we have been directed towards synthesizing single-molecules with three (triazole, pyrazole, and thiazole) nuclei with an anticipated biological activity.

The above findings encourage synthesizing compounds that can be used to develop novel antimicrobial, antifungal, and antioxidant agents with improved therapeutic efficacy. Herein, we have described the synthesis of a series of 1,2,3-triazole pyrazole containing thiazole derivatives, carried out their molecular docking studies on *S. aureus* topoisomerase IV enzyme and we have also studied them as potential SARS CoV2 main protease enzyme (Mpro) inhibitors.

## Results and discussion

### Synthesis and characterization

The reported compound (Z)-4-(1-(2-phenylhydrazono)ethyl) phenol derivative **3** synthesized in good yield by the nucleophile addition reaction of 1-(4-hydroxyphenyl) ethanone **1** with phenylhydrazine **2** in acetic acid at reflux temperature. Further compound **3** underwent Vilsmeier-Haack cyclization in the presence of DMF and POCl_3_ to produce pyrazole-4-carbaldehyde derivative **4** in an excellent yield [35]. Further pyrazole-4-carbaldehyde **4**, was subjected to alkylation with propargyl bromide **5** in the presence of potassium carbonate at the room temperature to obtained the good yield of corresponding 1-phenyl-3-(4-(prop-2-yn-1-yloxy)phenyl)-1H-pyrazole-4-carbaldehyde **6.** The targeted 1,4-disubstituted 1,2,3-triazole derivatives **8a–c** were synthesized by the cycloaddition of 1-phenyl-3-(4-(prop-2-yn-1-yloxy)phenyl)-1H-pyrazole-4-carbaldehyde **6** with various substituted aromatic azides **7a-c** individually by using of click chemistry in the presence of CuSO_4_.5H_2_O, sodium ascorbate [36].

All the compounds **8a-c** were obtained in moderate to be good yields and the structures of all compounds **8a-c** were confirmed by their spectroscopic data. In the proton NMR spectrum of 1-phenyl-3-(4-((1-phenyl-1H-1,2,3-triazol-4-yl)methoxy)phenyl)-1H-pyrazole-4-carbaldehyde (**8a**), the two signal singlet at δ 9.98, 8.43 ppm was assigned to the CHO, pyrazole protons respectively. The fourteen aromatic protons and one triazole proton have resonated at δ 7.76–7.12 ppm as multiplet. The signals at δ 5.30 ppm can be assigned to methylene protons. In the ^13^C NMR spectrum of 8a, the chemical signal δ 184.03 showed due to C=O functional group. The chemical signals at δ 158.9 − 126.91 ppm was attributed to the aromatic ring carbons. The chemical shift signals δ 153.7, 130.32, and 118.7 ppm were assigned to positions of pyrazole moiety. The chemical signals at δ 148.8 and 118.7 ppm were attributed to the triazole. The signals at δ 63.21 ppm can be assigned to methylene protons. The mass spectrum characterization of the compound **8a** exhibits an ion peak at 422 m/z, which can be designated as the ([M + H]^+^) ion peak, corresponding to a molecular formula C_25_H_19_N_5_O_2_. In order to construct the thiazole unit, the 1,4-disubstituted 1,2,3-triazole derivatives **8a–c** were each treated with thiosemicarbazamide **9** individually to give the compounds **10a-c** in excellent yield.

**Scheme 1.**
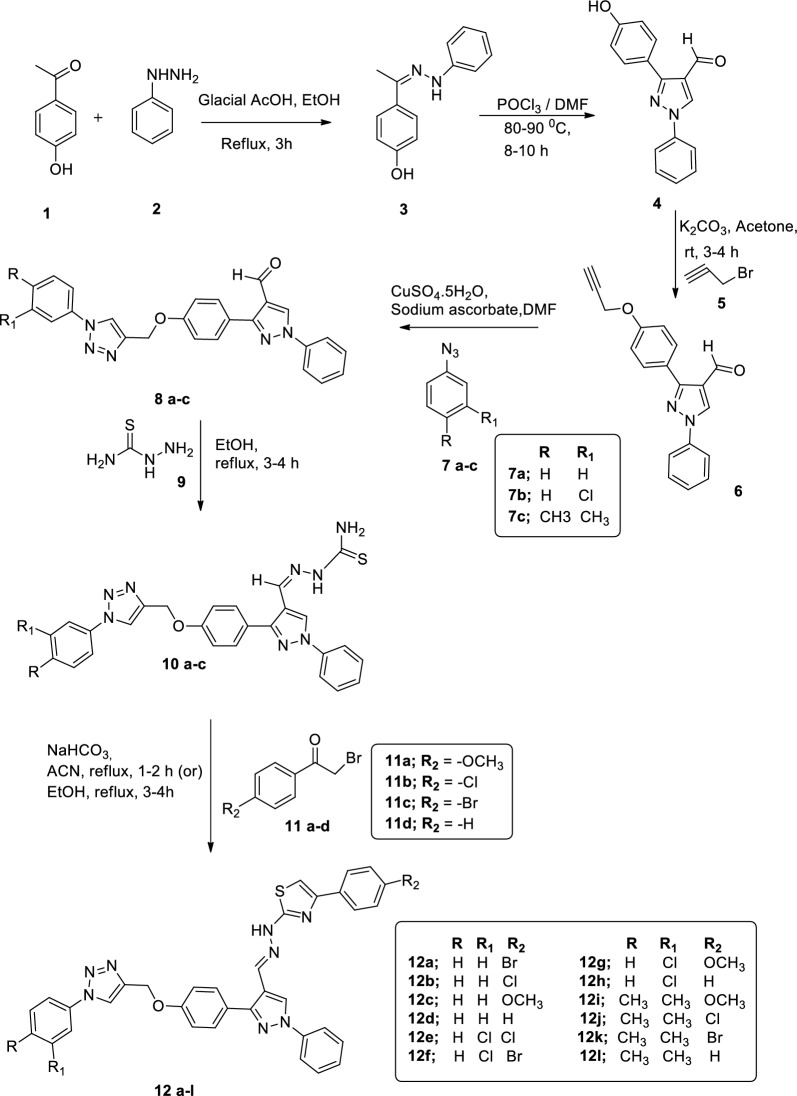
Synthesis of triazole, pyrazole contain 2,4-disubstituted thiazole analogs 12a-l

Finally, the desired compounds **12a-l** were prepared in moderate to good yields via the Hantzsch reaction of compounds **10a-c** treated with various substituted phenacyl bromides **11a-d** in the presence of sodium bicarbonate under reflux conditions [37]. The IR spectrum characterization of compound 4-(4-bromophenyl)-2-(2-((1-phenyl-3-(4-((1-phenyl-1H-1,2,3-triazol-4-yl)meth-oxy)-phenyl)-1H-pyrazol-4-yl)methylene)hydrazinyl)thiazole (**12a**) showed characteristic absorption band from 3073 cm^−1^, 2929 cm^−1^ due to C-H aromatic vibrations. In the proton NMR spectrum of compound (**12a**), the CH=N proton has resonated as a singlet at δ 8.25 ppm. The pyrazole, triazole, and thiazole proton showed as a singlet at δ 8.09, 7.70 and 6.83 respectively. The multiplet at δ 7.79 –7.09 ppm values can be attributed to the aromatic protons of compound (**12a**). The singlet at δ 5.37 ppm was assigned to the bridged methylene protons. In the ^13^C NMR spectrum of compound (**12a**), the chemical signal δ 175.4, 158.3 and 103.7 ppm were assigned to the thiazole. The chemical signal δ149.4, 130.0, 114.7 were assigned to the pyrazole. The triazole chemical signals conformed at δ 135.6 and 119.2. The chemical signals at δ 169.01–115.1 ppm were attributed to the aromatic ring carbons and chemical signals at δ 135.5 and 62.36 ppm CN and bridged methylene protons respectively. The mass spectrum characterization of the compound (**12a**) ESI–MS spectra showed the obtained peak at m/z = 672.85 ([M + H]^+^) and corresponding to a molecular formula C_34_H_25_N_8_OSBr. The chemical structures of newly synthesized compounds **12b-l** were confirmed by IR, ^1^H NMR, ^13^CNMR, and HRMS spectral data. The synthetic route to target compounds is given in Scheme 1.

### Anti-microbial activity

The antibacterial properties of synthesized target compounds (**12a-l)** were evaluated using two strains of Gram-positive bacteria (*Bacillus subtilis*, *Staphylococcus aureus)* and two strains of Gram-negative bacteria (*Escherichia coli, Pseudomonas aeruginosa).* The Minimum Inhibitory Concentration (MIC) [38] of the compounds against a bacterial activity was determined using the broth dilution method and is represented in the below Table 1**.**

**Table 1:** MIC of synthesized **12a-l**compounds (μg/ml) against gram-positive and gram-negative bacterial strains

Compound	Gram positive bacteria		Gram negative bacteria	
	*S. aureus*	*B. subtilis*	*P. aeruginosa*	*E. coli*
	MTCC 96	MTCC 441	MTCC 424	MTCC 443
**12a**	6.7 ± 0.06	6.7 ± 0.06	9.5 ± 0.06	10.0 ± 0.08
**12b**	7.2 ± 0.05	6.1 ± 0.08	9.8 ± 0.03	10.0 ± 0.08
**12c**	NA	NA	NA	NA
**12d**	NA	NA	NA	NA
**12e**	**4.8 ± 0.03**	**6.2 ± 0.03**	**9.8 ± 0.03**	**10.0 ± 0.06**
**12f**	**5.1 ± 0.08**	**6.2 ± 0.03**	**8.7 ± 0.08**	**10.0 ± 0.06**
**12 g**	8.0 ± 0.03	7.2 ± 0.05	9.8 ± 0.03	10.0 ± 0.06
**12 h**	7.2 ± 0.05	8.0 ± 0.03	9.5 ± 0.06	10.0 ± 0.08
**12i**	9.8 ± 0.03	7.2 ± 0.05	9.8 ± 0.03	> 50
**12j**	8.7 ± 0.08	8.0 ± 0.03	10.0 ± 0.06	> 25
**12 k**	**4.1 ± 0.03**	**6.2 ± 0.03**	**9.5 ± 0.06**	** > 25**
**12 l**	9.5 ± 0.06	6.2 ± 0.03	9.5 ± 0.06	> 50
*Novobiocin*	**3.9 ± 0.03**	**NT**	**NT**	**NT**
*Ampicillin*	**10.0 ± 0.08**	**10.0 ± 0.06**	**10.0 ± 0.08**	**3.9 ± 0.03**

*NT* Not tested

The bold values in the table were found to be either more potent or comparable to the standard reference

For the tested compounds, it is noted that all compounds were found to be very effective against the growth of microbial activity when compared with standard antibacterial agents. Among all twelve compounds the chemical compounds **12e, 12f,** and **12** k are profound to be better agents with significant growth inhibitory activity against all the tested strains and when compared to the standard references Novobiocin and Ampicillin as shown in Table 1. It can be seen, thatthe compounds **12e, 12f,** and **12 k** containing a para-bromo attached phenyl group directly bound to the thiazole ring showed the highest inhibitory activity, it may be attributed negative inductive effect of the Cl, Br group enhanced the activity. Also, compounds with p-chloro positions and additional -CH_3_ and -OCH_3_ substituents in the -phenyl ring (-Ph) showed seven folds of activity against *Escherichia coli* compared to standard drug, It is possible that lipophilicity of the molecules plays an important role in activity. These results reveal that compounds containing electron-donating groups promote high antibacterial activity. The only exception is, the compound **12d, **which has only aromatic group attached compound, which did not show any significant inhibitory activity towards tested microbial spit indicating presence of substituents important for activity.

The antifungal activities of the synthesized compounds **12a-l** were tested against filamentous fungi, *Aspergillus niger* MTCC 404 and *Saccharomyces cerevisiae* MTCC 1344 yeast cultures. The steak plate method was used to identify the minimum concentration of compound required to inhibit the fungal growth, which was determined by the zone of precise area length (mm) on the YEP agar plate. The experiments were performed in triplicates, represented the data in the form of Mean ± SEM, as shown in the below Table 2**.**

**Table 2:** Zone of fungal growth inhibition (mm) upon incubation with compound at 10 and 20 µM concentrations after 2 days at 37 °C

Compound	*Aspergillus niger*		*Saccharomyces cerevisiae*	
	MTCC 404		MTCC 1344	
	10 µM	20 µM	10 µM	20 µM
**12a**	**3.0 ± 0.11**	**5.5 ± 0.05**	**6.0 ± 0.08**	**6.5 ± 0.05**
**12b**	2.0 ± 0.57	2.0 ± 0.48	1.5 ± 0.03	2.0 ± 0.57
**12c**	NT	NT	NT	NT
**12d**	NT	NT	NT	NT
**12e**	3.0 ± 0.11	3.0 ± 0.11	1.5 ± 0.14	3.0 ± 0.11
**12f**	**7.3 ± 0.06**	**7.4 ± 0.05**	**6.2 ± 0.05**	**6.8 ± 0.05**
**12 g**	3.0 ± 0.11	3.0 ± 0.11	**6.0 ± 0.08**	**6.0 ± 0.08**
**12 h**	2.1 ± 0.03	3.0 ± 0.11	2.0 ± 0.00	3.0 ± 0.11
**12i**	1.5 ± 0.05	2.0 ± 0.00	**6.0 ± 0.08**	**6.2 ± 0.05**
**12j**	2.1 ± 0.03	3.0 ± 0.11	2.0 ± 0.00	2.0 ± 0.00
**12 k**	**6.8 ± 0.00**	**7.0 ± 0.08**	**6.2 ± 0.05**	**6.6 ± 0.05**
**12 l**	1.5 ± 0.05	2.0 ± 0.06	1.5 ± 0.03	2.0 ± 0.00
*Miconazole*	8.0 ± 0.11	10.0 ± 0.11	8.0 ± 0.11	9.0 ± 0.00

*NT* Not tested

The bold values in the table were found to be either more potent or comparable to the standard reference

The results clearly suggested that among all the synthesized analogs, **12a**, **12f,** and **12 k** showed prominent antifungal activity against *Aspergillus niger* MTCC 404, and compounds **12a** and **12f, 12 g, 12i,** and **12 k** showed potent fungal growth inhibition against *Saccharomyces cerevisiae* MTCC 1344 are reported in Table 2.

### Antioxidant activity

The DPPH (2,2-diphenyl-1-picrylhydrazyl) free radical-scavenging assay is usually employed to evaluate the antioxidant ability of newly derived compounds. The present study measured the free radical scavenging activity of compounds by the DPPH assay method. 0.5 mM solution of DPPH in methanol was prepared, and 100 μL of this solution was added to various concentrations of DMSO dissolved compounds ranging from 10 µM to 200 µM. The absorbance was measured at 517 nm upon incubation in the dark at room temperature for 30 min [39]. All the tests were performed in triplicate, and the percent antioxidant inhibitory activities were calculated by comparing the values of absorbance of the control and test samples. Dose-responsive curves were plotted against the concentration of the compound for percent free radical scavenging activity. The antioxidant activity results are represented in Table 3.

**Table 3:** Percent antioxidant activity of synthesized **12a-l** compounds

Compound	Compound concentration			
	10 µM	50 µM	100 µM	200 µM
**12a**	39.9	41.6	41.9	55.5
**12b**	24.09	24.4	25.1	27.3
**12c**	NT	NT	NT	NT
**12d**	NT	NT	NT	NT
**12e**	− 5.9	− 5.8	14.5	30.9
**12f**	**42.1**	47.1	**56.1**	**67.8**
**12 g**	22.5	29	33.8	42.2
**12 h**	24.09	24.4	25.1	27.3
**12i**	29.6	39.2	39.4	40.2
**12j**	24.4	24.8	26.9	28.1
**12 k**	**51.8**	58.6	**59.2**	**67**
**12 l**	− 18.9	− 19	− 19.9	− 16.8
**AA**	44.1	44.5	45.8	79.3

*NT* Not tested*; AA* Ascorbic acid (Standard)

The bold values in the table were found to be either more potent or comparable to the standard reference

**Fig. 1:**
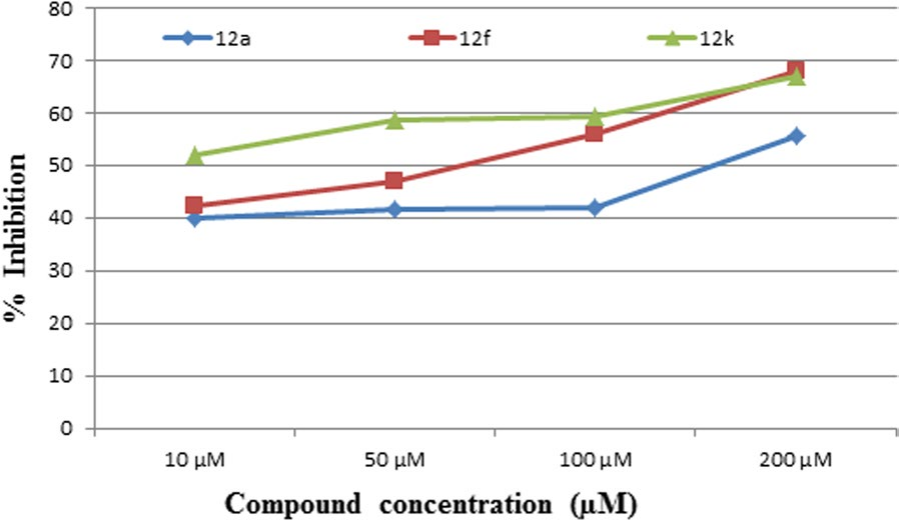
Dose-responsive curves were plotted against the concentration of compound for percent free radical scavenging activity

From the results, it is noticed that all synthesized **12a-l** analogues were found to be potent in donating hydrogen free radicals except **12i,** which showed a poor radical scavenging capacity. Compounds **12f **and **12 k** proved to be more or comparatively potent than the standard reference ascorbic acid and are shown in Table 3 and Fig. 1.

### Molecular docking studies on *S.aureus* topoisomerase IV enzyme

The molecular docking studies were performed using Vina in PyRx [40, 41] docking tool to predict the protein–ligand interactions at the molecular level. The co-crystal structure of Gram-positive *S. aureus* topoisomerase IV enzyme of the catalytic domain in complex with novobiocin (PDB ID: 4URN) is used as a target. The crystal structure of 4URN bound with novobiocin as inhibitor defined hydrogen bonding with Lys36, Asn49, Asp52, Asp76, Arg79, Arg138 and hydrophobic interaction with Asp35, Lys36 and Ile96. The 3D grid box was configured with dimensions of size_x = 24.4292534438, size_y = 36.3029041501, size_z = 27.787369965 and exhaustiveness of 8. It also showed π-cationic interaction with Lys36 and a salt bridge with Arg79, water bridges were found with Ser50, Gly78 and Thr168. All the synthesized compounds showed hydrogen bond interactions with the target protein except compound **12 l**. Amino acid residues Thr34, Glu45, Asn49, Asp52, Glu53, Asn56, Asp76 and Gly80 of 4URN were involved in H-bond interactions similar to that of novobiocin. And amino acid residues Asp35, Lys36, Ile46, Asn49, Asn56, Gly80, Met81, Pro82, Ile96, Gly121, Ala122, Val170, Lys180 and Ala181 were involved in hydrophobic interactions with topoisomerase IV enzyme. The binding affinities of compounds **12a-l** were ranging from -10.0 to -11.0 kcal/mol, values are shown in Table 4.

**Table 4:** Binding affinity of Compounds **12a-l** and interacting amino acid residues of topoisomerase IV enzyme (PDB ID: 4URN)

Compound	Binding affinity (kcal/mol)	Interacting amino acid	
		H-bond	Hydrophobic
12a	− 10.4	Thr34, Asn56, Asp76,	Asp35, Lys36, Ile46, Asn49, Asn56, Gly80, Met81, Pro82, Ile96, Gly121, Ala122, Val170, Lys180, Ala181
12b	− 10.6	Asn49, Asp52	Lys36, Glu45, Asn56, Asp76, Arg79, Met81, Pro82, Ile96, Lys180, Ala181
12c	− 10.4	Asn49, Asp52, Gly80	Thr34, Lys36, Asn49, Glu53, Met81, Ile96, Arg138, Lys180, Ala181
12d	− 10.7	Asn49, Asp52	Lys36, Arg79, Met81, Pro82, Ile96, Arg138, Lys180
12e	− 10.6	Asp52, Gly80	Lys36, Asn49, Glu53, Met81, Ile96, Arg138, Lys180, Ala181
12f	− 10.3	Asp52	Lys36, Val51, Asp52, Glu53, Arg79, Pro82, Ile96, Thr177, Lys180, Ala181, Leu201
12 g	− 10.1	Glu53, Gly80	Thr34, Asp35, Lys36, Glu45, Asp52, Glu53, Met81, Pro82, Ile96, Arg138, Lys180, Ala181
12 h	− 11.0	Asp52, Glu53, Gly80	Lys36, Asn49, Glu53, Met81, Pro82, Ile96, Arg138, Lys180, Ala181
12i	− 10.6	Asn49, Asp52, Gly80	Thr34, Lys36, Asn49, Glu53, Gly80, Met81, Pro82, Ile96, Ala122, Arg138, Ile178, Lys180, Ala181
12j	− 10.7	Glu45, Asn49, Asn56	Thr34, Lys36, Ile46, Asn49, Asp52, Glu53, Met81, Pro82, Ile96, Phe97, Ala122, Val170, Ile178, Lys180, Ala181
12 k	− 10.6	Asp52, Gly80	Thr34, Lys36, Asn49, Glu53, Met81, Pro82, Ile96, Arg138, Lys180, Ala181
12 l	− 10.0	–	Lys36, Ile46, Glu53, Tyr58, Arg79, Met81, Pro82, Ile96, Phe97, Ala122, Arg138, Val170, Lys180, Ala181

**Fig. 2:**
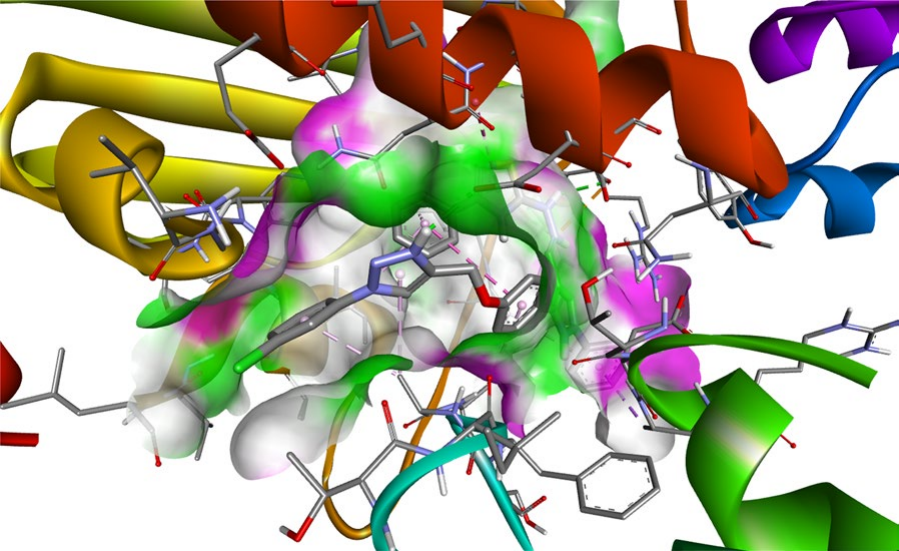
Docking pose of compound **12 h** with topoisomerase IV enzyme (PDB ID: 4URN)

**Fig. 3:**
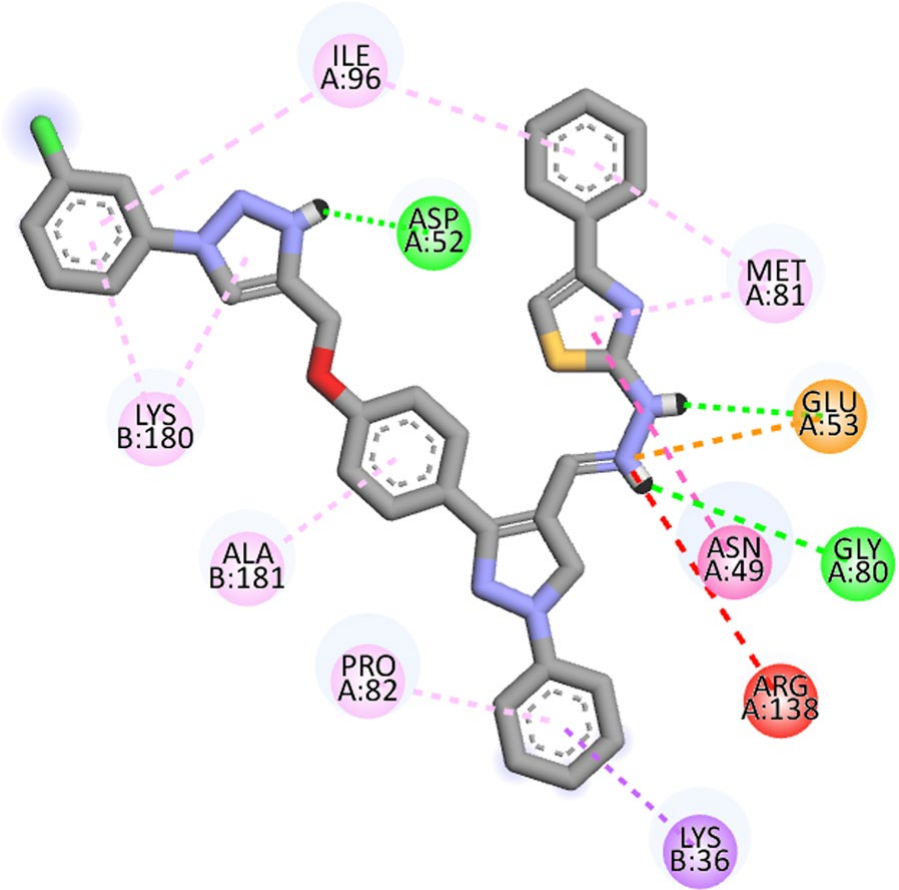
2D interactions of compound **12 h** with topoisomerase IV enzyme (PDB ID: 4URN)

**Fig. 4:**
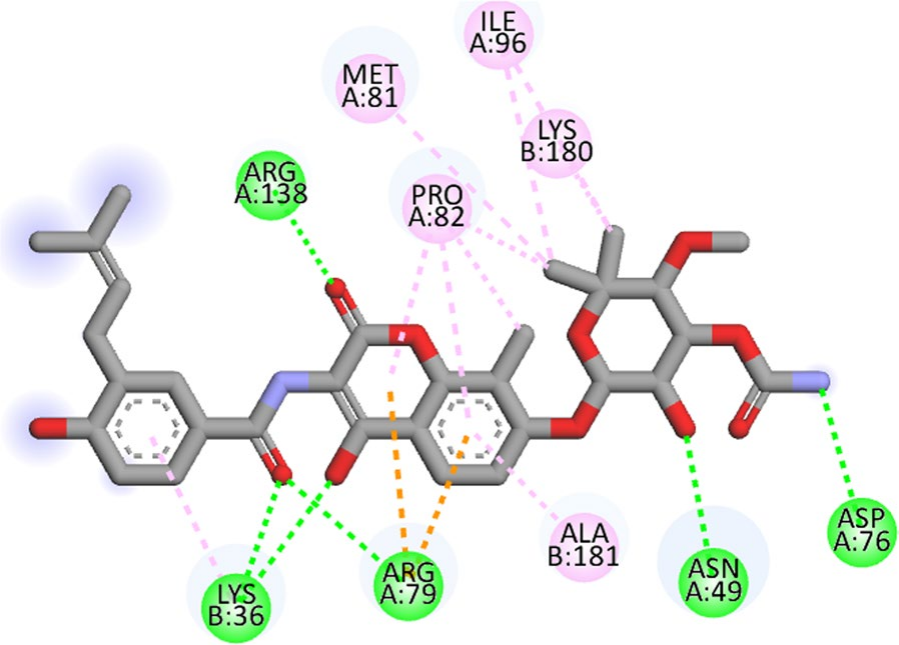
2D interactions of novobiocin in crystal structure of topoisomerase IV enzyme (PDB ID: 4URN)

**Fig. 5:**
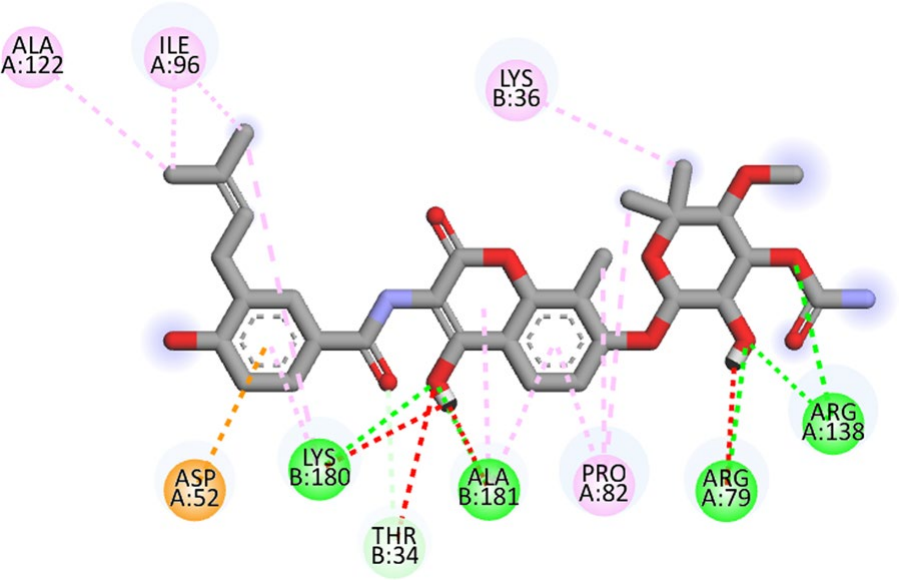
Binding interactions of re-docked Novobiocin with topoisomerase IV enzyme (PDB ID: 4URN)

**Fig. 6:**
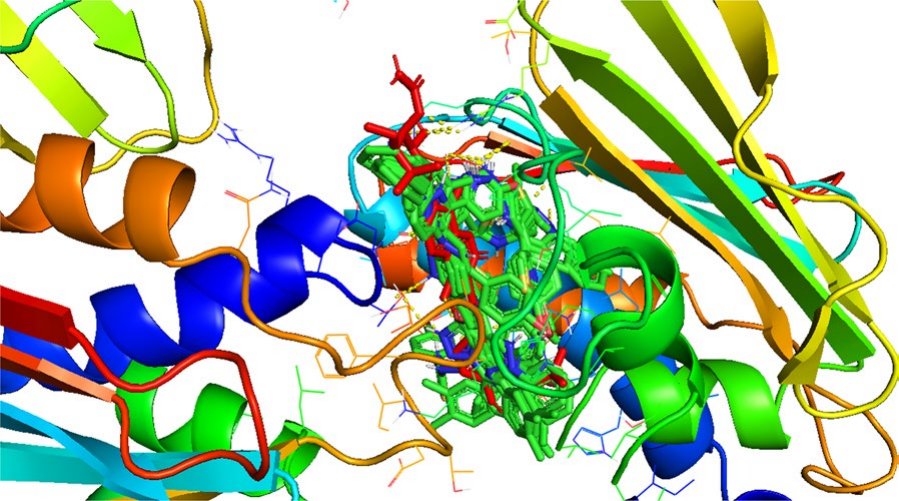
Super imposed image of all ligands (green colour) with co-crystallized ligand Novobiocin (red colour)

Compound **12 h** showed the highest docking score of about − 11.0 kcal/mol. It was involved in H-bond interactions with Asp52, Glu53 and Gly80 of 4URN and hydrophobic interactions with Lys36, Asn49, Glu53, Met81, Pro82, Ile96, Arg138, Lys180 and Ala181 of the same shown as (Figs. 2, 3, 4). The standard compound ascorbic acid was involved in only H-bond interactions with Asn49, Glu53, Asp76 and Glu80 of topoisomerase and hydrophobic interactions were absent. The docking results also validated by re-docking the co-crystallized ligand novobiocin, which scored a binding energy value of − 9.1 kcal/mol (RMSD = 1.077 Å). It produced key interactions with Arg79, Arg138, Lys180, Lys181 and hydrophobic interaction with Tr34, Lys36, Thr37, Asp52, Arg79, Pro82, Ile96, Ala122, Lys180 and Lys181 of 4URN (Fig. 5), and some of these interactions are comparable to the ligand interactions **12a-l**. Produced the super imposed image (Fig. 6) of all ligands with co-crystallized ligand novobiocin, it has showed good structural coincidence, which reveal that they could best fit into the cavity of *S. aureus* topoisomerase IV enzyme.

### Molecular docking studies on COVID-19 main protease

**Fig. 7:**
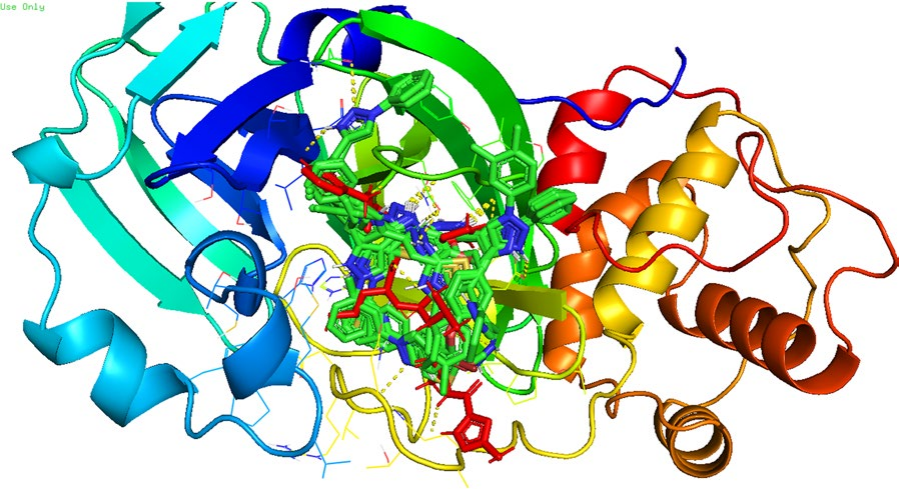
Best confirmers of ligands (green coloured) super imposed with N3 inhibitor (red coloured) in cavity of 6LU7

The prevailing pandemic conditions motivated us to carry out in silico screening against COVID-19, as a reason the crystal structure of COVID-19 main protease in complex with an N3 inhibitor (PDB ID: 6LU7) was used as the SARS CoV-2 target [42, 43]. In the crystal structure of 6LU7 in complex with N3 inhibitor, defined H-bond interactions with Phe140, Gly143, His164, Glu166, Gln189 and Thr190 and also hydrophobic interactions with His41, Met49, Met165, Leu167, Pro168, His172 and Ala191. The 3D grid box was assigned with dimensions of size_x = 19.8254655071, size_y = 21.369112532, size_z = 24.1916496015 and exhaustiveness is 8. All the synthesized ligand molecules showed H-bond interactions similar to the N3 inhibitor. Among amino acid residues Phe140, Leu141, Gly143, His163, His164, Met165, Glu166, Leu167, Arg188, Gln189 and Thr190 any one or more number of amino acids were involved in H-bond interactions with ligands. The hydrophobic interactions were involved by Thr24, Leu27, His41, Met49, Phe140, Ser144, Cys145, Met165, Glu166, Pro168, Gln189, Thr190 and Ala191 of 6LU7. The binding affinities of compounds **12a-l** were ranging from -8.2 to -9.3 kcal/mol, values are shown in Table 5. To validate the results the co-crystallized ligand N3 inhibitor re-docked into the active site pocket of 6LU7. The binding energy of N3 inhibitor was -8.0 kcal/mol (RMSD = 0.953 Å), and a super imposed image of ligands with N3 inhibitor is produced (Fig. 7). The figure explains that ligands structures are very much super imposed with N3 inhibitor structure as a result they best fit into the active site pocket of main protease.

**Table 5:** Docking scores of compounds 12a -12 l and interacting amino acids of COVID-19 main protease (PDB ID: 6LU7)

Compound	Binding affinity (kcal/mol)	Interacting amino acid	
		H-bond	Hydrophobic
12a	− 9.1	Phe140, Leu141, His163, Met165	His41, Ser144, Cys145, Glu166
12b	− 8.8	Leu141, Gly143, His163	Met49, Phe140, Ser144, Cys145, Glu166, Pro168, Thr190, Ala191
12c	− 8.3	Leu141, Gln189	Thr26, Leu27, His41, Met49, Ser144, Cys145, Pro168
12d	− 9.2	Leu141, Glu166, Gln189	His41, Ser144, Cys145, Met165, Glu166
12e	− 9.3	Phe140, Leu141, His163, Met165	His41, Phe140, Gly143, Cys145, Met165, Glu166, Gln189
12f	− 9.2	Phe140, Leu141, His163, Met165, Arg188	His41, Phe140, Gly143, Cys145, Met165, Glu166, Gln189
12 g	− 8.8	Glu166, Leu167	Met49, Asn142, Cys145, Met165, Glu166, Gln189, Thr190, Ala191,
12 h	− 8.7	Glu166, Leu167, Thr190	Leu27, His41, Met49, Cys145, Met165, Glu166, Pro168, Gln189, Thr190, Ala191
12i	− 8.7	Asn142, His164	Thr26, His41, Met49, Phe140, Gly143, Cys145, Met165, Glu166, Pro168
12j	− 8.3	Asn142, Thr190	His41, Cys145, Met165, Glu166, Leu167, Pro168, Gln189
12 k	− 9.1	Glu166. Leu167	Leu27, His41, Met49, Cys145, Met165, Glu166, Thr190, Ala191
12 l	− 8.2	Asn142, Thr190	Cys145, Met165, Glu166, Pro168

**Fig. 8:**
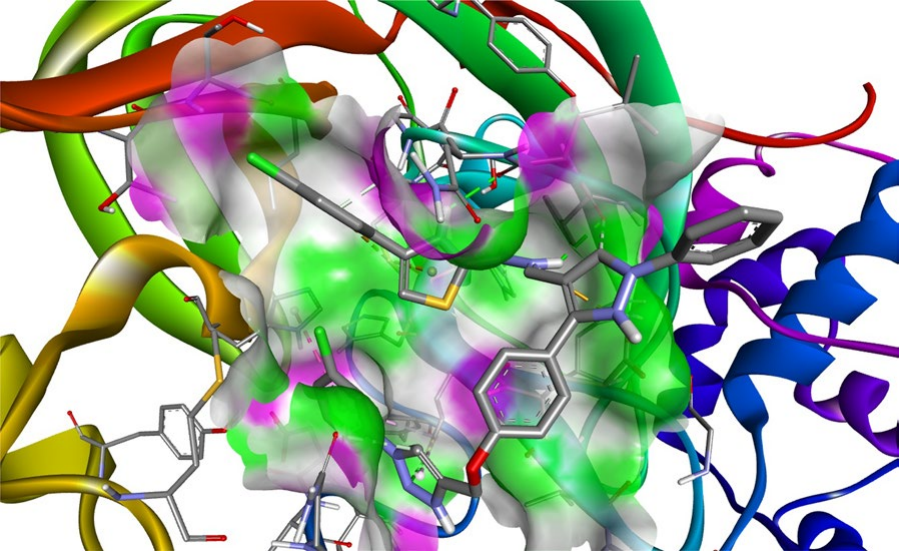
Docking pose of compound **12e** with COVID-19 main protease (PDB ID: 6LU7)

**Fig. 9:**
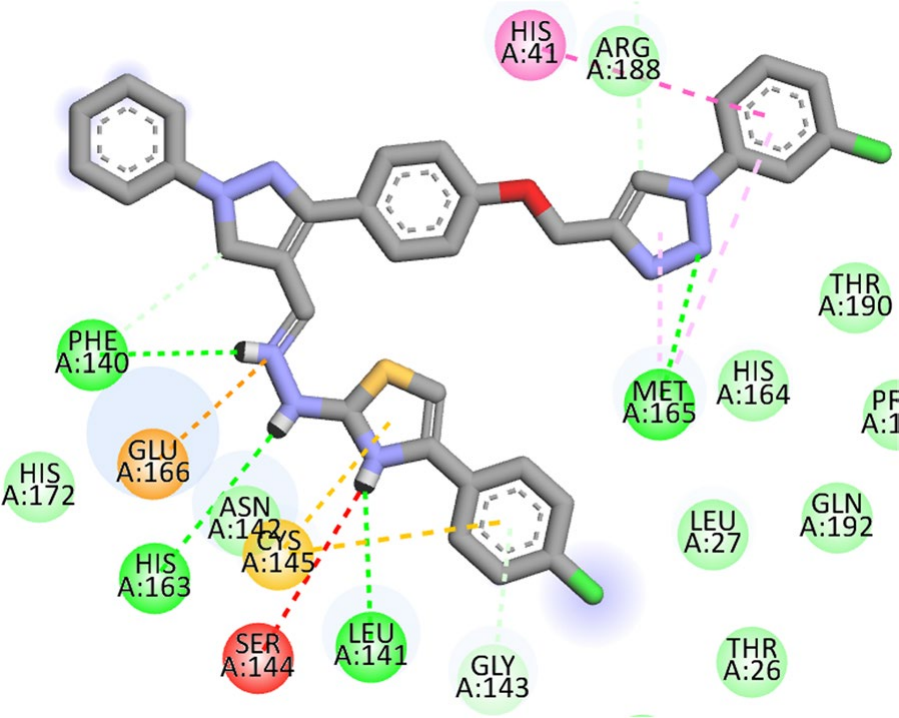
2D interactions of compound **12e** with COVID-19 main protease (PDB ID: 6LU7)

**Fig. 10:**
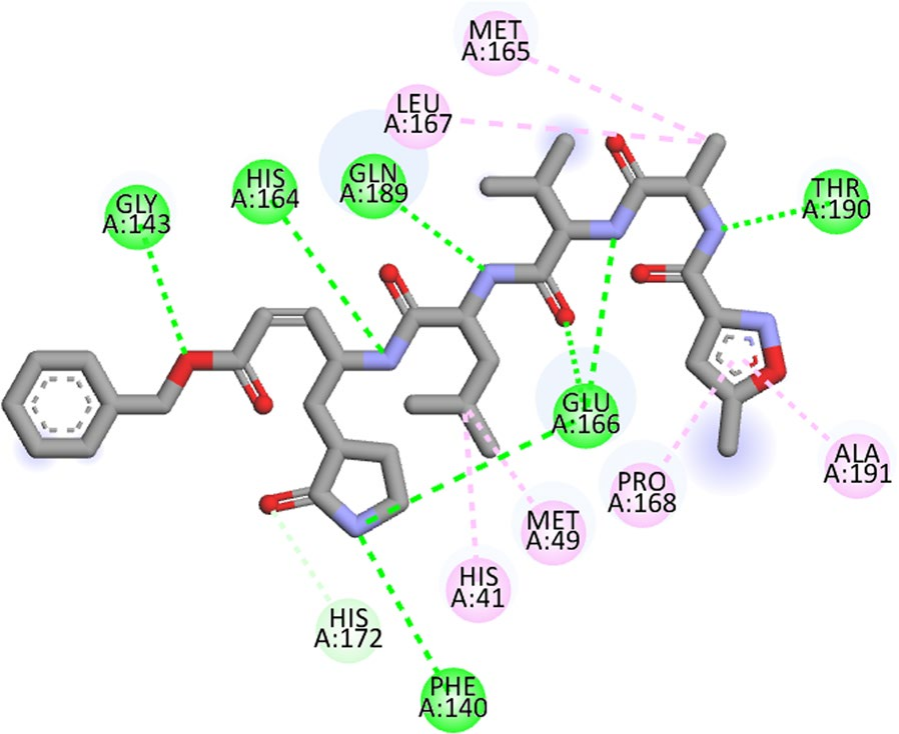
2D interactions of N3 inhibitor in crystal structure of COVID-19 main protease (PDB ID: 6LU7)

Compound 12e showed the highest docking score about -9.3 kcal/mol. It was involved in H-bond interactions with amino acid residues Phe140, Leu141, His163 and Met165 of 6LU7 and hydrophobic interactions with amino acid residues His41, Phe140, Gly143, Cys145, Met165, Glu166 and Gln189 of main protease (Figs. 8, 9, 10). The docking study reveals that all the newly synthesized molecules could be the inhibitors of COVID-19 main protease similar to the N3 inhibitor.

## Experimental section

### Materials and methods

All reactions were performed in oven-dried glassware. The melting points of the compounds **12a-l** were measured in open capillaries and are uncorrected. The NMR spectral analysis was done on a Bruker AV 400 MHz instrument using deuterated samples with TMS as an internal standard. The chemical shifts were measured in ppm against internal TMS. The IR spectral analysis was done on a Perkin–Elmer 337 spectrophotometer for solid samples in KBr pellets. On Merck silica gel 60 F254 plates, the thin layer chromatography (TLC) was carried out, and the spots were visualized with UV light at 254 nm of wavelength or by staining with aqueous basic potassium permanganate. Flash column chromatography was carried out on a Merck silica gel 60A0 (100–200 mesh). We used commercially available reagents as supplied, and some of them were distilled if required.

### Chemistry gelation procedure

#### Synthesis of 3-(3-hydroxyphenyl)-1-phenyl-1H-pyrazole-4-carbaldehyde (4, C_16_H_12_N_2_O_2_)

The starting compound 3-(3-hydroxyphenyl)-1-phenyl-1*H*-pyrazole-4-carbaldehyde was synthesized permitting to our earlier reported methods. Briefly, 4-hydroxy acetophenones (1.0 mol) **(1)** were treated with substituted phenylhydrazine (1.2 mol) **(2)** to yield corresponding hydrazones **(3)**, followed by the Vilsmeier-Haack cyclization in the presence of DMF/POCl_3_ to give the desired compound in 85% yield, which in anagreement with previously reported data.

#### Synthesis of 1-phenyl-3-(4-(prop-2-yn-1-yloxy)-phenyl)-1H-pyrazole-4-carbaldehyde (6, C_19_H_14_N_2_O_2_)

The compound (**6**), (1.510 g, 1.0 mol) was prepared by the propargylation of compound 3-(3-hydroxyphenyl)-1-phenyl-1H-pyrazole-4-carbaldehyde (4), (1.321 g, 1.0 mol), using the propargyl bromide **5**, (0.515 g, 1.5 mol) in DMF (30 mL), under nitrogen condition for 3 h at room temperature. After completion (monitored by TLC), the reaction mixture was extracted using ice-cold water (3 × 50 mL) and DCM (2 × 50 mL). Then the combined organic layer was dried with anhydrous sodium sulfate, concentrated in vacuo, and purified by silica gel column chromatography using 20% ethyl acetate in hexane as an eluent to afford 1-phenyl-3-(4-(prop-2-yn-1-yloxy)phenyl)-1H-pyrazole-4-carbaldehyde (5), as light-yellow solid in 83% yield. Spectral data of isolated compound 6 is in agreement with the reported data. Yield 83%; m.p.:104–106 °C;^1^H NMR (400 MHz, CDCl_3_) (ppm):10.1 (s, 1H, CHO), 8.43 (s, 1H, pyrazole), Ar–H [7.91 (t, 1H, *J* = 7.05 Hz), 7.46–7.43 (m, 4H), 7.40–7.42 (m, 2H), 7.10–7.08 (m, 2H)], 4.79 (s, 2H, CH_2_), 3.30 (s, 1H, CH). ^13^C NMR (100 MHz, CDCl_3_, ppm): 197.1 (CO), Ar–C [151.2 (C), 145.2 (C), 138.2 (CH), 136.6 (CH), 130.4 (C), 128.2 (C), 119.0 (CH), 111.5 (CH)], pyrazole-C [150.7 (C), 124.3 (C), 113 (CH)], 45.5 (C), 26.9 (C), 25.9 (CH). IR: *v* = 3295, 1698 cm^−1^; ESI–MS: *m/z* calcd for C_19_H_14_N_2_O_2_ 302; found 303 [M + 1]^+^.

#### General procedure for the synthesis of substituted 1-phenyl-3-(4-((1-phenyl-1H-1,2,3-triazol-4-yl)methoxy)phenyl)-1H-pyrazole-4-carbaldehyde (8a-c)

To the compound 1-phenyl-3-(4-(prop-2-yn-1-yloxy)-phenyl)-1*H*-pyrazole-4-carbaldehyde **(6)** (0.1 eq) in DMF was added aryl azides **(7a-c)** (0.1 eq), in the presence of 10 mol % CuSO_4_.5H2O, sodium ascorbate and the reaction mixture was allowed to react at room temperature for 3–4 h. The reaction condition was monitored by TLC. After completion, added the ice-cold water and extracted with ethyl acetate, and the combined organic layers were removed under reduced pressure and purified by column chromatography (eluent; ethyl acetate: hexane 30:70) to get pure compounds **8 a-c** with a yield of 80–85%.

#### 1-phenyl-3-(4-((1-phenyl-1H-1,2,3-triazol-4-yl)methoxy)phenyl)-1H-pyrazole-4-carbaldehyde (8a, C_25_H_19_N_5_O_2_)

White solid; yield 80%; m.p.:144–146 °C; ^1^H NMR (400 MHz, CDCl_3_) (ppm): 9.98 (s, 1H, CHO), 8.43 (s, 1H, pyrazole), Ar–H [7.76 (d, *J* = 4.7 Hz, 2H), 7.72 (d, *J* = 7.6 Hz, 3H), 7.50–7.40 (m, 7H), 7.31 (d, *J* = 6.9 Hz, 1H), 7.12 (s, 2H)], 5.30 (s, 2H).^13^C NMR (100 MHz, CDCl_3_, ppm): 184.03 (CHO), Ar–C [158.9 (C), 138.56 (C), 138.01 (C), 136.67 (C), 135.58 (CH), 130.32 (CH), 129.39 (CH), 128.96 (CH), 128.67 (CH), 128.03 (CH), 126.91 (CH), pyrazole-C [153.70 (C), 130.32 (CH), 118.70 (C)],triazole-H [148.8 (C), 118.70 (CH)]0.63.21 (CH_2_). IR: *v* = 3452, 2929, 1730, 1602, 1592, 1450, 1235, 1045 cm^−1^; Anal.Calcd. For C_25_H_19_N_5_O_2_: C, 71.25; H, 4.54; N, 16.62; Found: C, 71.24; H, 4.52; N, 16.64. ESI–MS: *m/z* calcd for C_25_H_19_N_5_O_2_421; found 422 [M + 1]^+^.

#### 3-(4-((1-(3-chlorophenyl)-1H-1,2,3-triazol-4-yl)methoxy)phenyl)-1-phenyl-1H-pyrazole-4-carbaldehyde (8b, C_25_H_18_ClN_5_O_2_)

White solid; yield 80%; m.p.: 142–144; ^1^H NMR (400 MHz, CDCl_3_) (ppm): 9.99 (s, 1H, CHO), 8.46 (s, 1H, pyrazole), 7.52 (s, 1H, triazole), Ar–H [7.72 (d, *J* = 7.8 Hz, 2H), 7.63 (d, *J* = 7.3 Hz, 2H), 7.36–7.38 (m, 7H), 7.11 (s, 2H),] 5.30 (s, 2H, CH_2_). ^13^C NMR (100 MHz, CDCl_3_, ppm): 185.03 (CHO), Ar–C [157 (C), 139.03 (C), 136.01 (C),131.47 (C–Cl), 131.31 (CH), 131.0 (CH), 130.39 (CH), 129.68 (C), 129.0 (CH), 127.93 (CH), 124.53 (CH), 121.82 (CH), 119.72 (CH), 115.06 (CH), pyrazole-C [154.72 (C), 131.32 (CH), 115.06 (C)], triazole-H [144.82 (C), 119.72(C), (CH)]. 63.00 (CH_2_). IR: v = 3451, 2927, 1732, 1602, 1591, 1453, 1236, 1044 cm^−1^; Anal.Calcd. For C_25_H_18_N_5_O_2_Cl: C, 65.86; H, 3.98; N, 7.78; Found: C, 65.87; H, 3.99; N, 7.76. ESI–MS: *m/z* calcd for C_25_H_18_N_5_O_2_Cl 455; found 456 [M + 1]^+^.

#### 3-(4-((1-(3,4-dimethylphenyl)-1H-1,2,3-triazol-4-yl)methoxy)phenyl)-1-phenyl-1H-pyrazole-4-carbaldehyde (8c, C_27_H_23_N_5_O_2_)

Light white solid; yield 80%; m.p.:148–150 °C; ^1^H NMR (400 MHz, CDCl_3_) (ppm): 10.08 (s, 1H, CHO), 8.55 (s, 1H, pyrazole), 7.73 (s, 1H, triazole), Ar–H [7.88–7.80 (m, 5H), 7.54 (t, *J* = 7.7 Hz, 3H), 7.42 (t, *J* = 6.4 Hz, 1H), 7.31 (d, *J* = 3.8 Hz, 1H), 7.22 (s, 2H)], 5.37 (s, 2H, CH_2_), 2.38 (s, 3H, CH_3_), 2.34 (s, 3H, CH_3_). ^13^C NMR (100 MHz, CDCl_3_, ppm): 185.06 (CHO), Ar–C [159.15 (C), 139.04 (C), 138.44 (C), 137.74 (C), 131.32 (CH), 130.86 (CH), 129.69 (CH), 127.93 (C), 124.53 (C), 122.39 (CH), 121.82 (CH), 117.95 (CH), pyrazole-C [139.04 (C), 130.68, (CH), 115.0, (C)], triazole-C [138.44 (C), 117.95 (CH)], 62.16 (CH_2_), 19.91 (CH_3_), 19.49 (CH_3_). IR: *v* = 3443, 2927, 1733, 1600, 1583, 1451, 1237, 1040 cm^−1^; Anal.Calcd. For C_27_H_23_N_5_O_2_: C, 76.94; H, 5.50; N, 9.97; Found: C, 76.93; H, 5.51; N, 9.96. ESI–MS: *m/z* calcd for C_27_H_23_N_5_O_2_449; found 450 [M + 1]^+^.

#### General procedure for the synthesis of 2-((1-phenyl-3-(4-((1-phenyl-1H-1,2,3-triazol-4-yl)methoxy)phenyl)-1H-pyrazol-4-yl)methylene)hydrazinecarbothioamide (10 a-c)

To a solution of substituted compound, **8a-c** (1.0 mmol) and ethanol (25 mL) was added thiosemicarbazamide **9** (1.0 mmol), and the reaction mixture was refluxed for 4 h. Progress of the reaction was monitored by TLC. After completion, EtOH was removed in a vacuum. The crude product was purified by silica gel column chromatography in the ratio of ethyl acetate: hexane (25:75) to afford the pure compound **10 a-c**.

#### General procedure for the Synthesis of 4-phenyl-2-(2-((1-phenyl-3-(4-((1-phenyl-1H-1,2,3-triazol-4-yl)methoxy)-ph-enyl)-1H-pyrazol-4-yl)methylene)hydrazinyl)thiazole (10 a-l)

To a solution of substituted compounds **10 a-c (1.0 eq)** and acetonitrile (25 mL) was added phenacyl bromide **11a-d** **(1.0 eq)** in the presence of sodium bicarbonate (0.5 eq), refluxed for 1–2 h. After completion (using TLC), acetonitrile was removed in vacuo. The crude product was purified by silica gel column chromatography in the ratio of ethyl acetate: hexane (30:70) to obtain the pure compound **12 a-l** in good yields.

#### 4-(4-bromophenyl)-2-(2-((1-phenyl-3-(4-((1-phenyl-1H-1,2,3-triazol-4-yl)meth-oxy)-phenyl)-1H-pyrazol-4-yl)methylene)hydrazinyl)thiazole (12a, C_34_H_25_BrN_8_OS)

White solid; yield 86%; m.p.: 184–186 °C; ^1^H NMR (400 MHz, CDCl_3_) (ppm): 8.25 (s, 1H, CH=N), 8.09 (s, 1H, pyrazole),7.70 (s, 1H, triazole), Ar–H [7.79 (d, *J* = 7.8 Hz, 2H), 7.75 (d, *J* = 7.7 Hz, 2H), 7.60 (dd, *J* = 8.1, 5.4 Hz, 4H), 7.52 (d, *J* = 5.9 Hz, 2H), 7.49–7.42 (m, 6H), 7.34 (d, *J* = 7.4 Hz, 1H), 7.09 (d, *J* = 8.6 Hz, 2H)], 6.83 (s, 1H, thiazole), 5.37 (s, 2H, CH_2_).^13^C NMR (100 MHz, CDCl_3_, ppm): thiazole-C [175.4 (C), 158.39 (C), 103.74 (CH)], Ar–C [169.01 (C), 139.50 (C), 131.79 (C), 131.79 (C), 130.92 (CH), 129.84 (CH), 129.52 (CH), 129.0 (CH), 128.86 (CH), 127.02 (CH), 127.04 (CH), 127.04 (CH), 126.43 (C), 120.94 (CH), 120.67 (CH), 118.58 (CH), 115.17 (CH)], pyrazole-C [149.43 (C), 130.05 (CH), 114.77(C)], triazole-C [135.60 (C), 119.29 (CH)],135.55 (CN), 62.36 (CH_2_). IR: *v* = 3668, 3451, 2929, 1692, 1593, 1451, 1237, 1045 cm^−1^; Anal. Calcd. For C_34_H_25_N_8_OSBr: C, 60.63; H, 3.74; N, 16.64; Found: C, 60.61; H, 3.70; N, 16.65. ESI–MS: *m/z* calcd for C_34_H_25_N_8_OSBr 671.7; found 672.85 [M + 1]^+^.

#### 4-(4-chlorophenyl)-2-(2-((1-phenyl-3-(4-((1-phenyl-1H-1,2,3-triazol-4-yl)meth-oxy)-phenyl)-1H-pyrazol-4-yl)methylene)hydrazinyl)thiazole (12b, C_34_H_25_ClN_8_OS)

White solid; yield 82%; m.p.: 188–190 °C; ^1^H NMR (400 MHz, CDCl_3_) (ppm): 8.35 (s, 1H, CH =N), 8.09 (s, 1H, pyrazole), 7.94 (s, 1H, triazole), Ar–H [7.79 (d, *J* = 7.9 Hz, 2H), 7.75 (d, *J* = 6.4 Hz, 2H), 7.67 (d, *J* = 8.4 Hz, 3H), 7.54–7.47 (m, 8H), 7.35 (d, *J* = 6.2 Hz, 2H), 7.14 (d, *J* = 7.7 Hz, 2H), 6.81 (s, 1H, thiazole), 5.38 (s, 2H, CH_2_). ^13^C NMR (100 MHz, CDCl_3_, ppm): thiazole-C [168.38 (C), 145.17 (C), 95.01 (CH)], Ar–C [155.59 (C), 141.71 (C), 138.56 (C), 137.83 (C), 130.10 (C), 129.54 (C), 129.03 (CH), 128.89 (CH), 127.2 (CH), 127.11 (CH), 125.65 (CH), 122.42 (CH), 120.89 (CH), 118.58 (CH), 105.98 (CH), 103.40 (CH), pyrazole-C [141.71 (C), 130.9 (CH), 114.99 (C)], triazole-C [137.83 (C), 119.33 (CH)], 137.83 (CN), 62.03 (CH_2_). IR: *v* = 3668, 3453, 2929, 1692, 1602, 1593, 1451, 1237, 1045 cm^−1^; Anal. Calcd. For C_34_H_25_ClN_8_OS: C, 64.91; H, 4.01; N, 17.81; Found: C, 64.90; H, 4.00; N, 17.82. ESI–MS: *m/z* calcd for C_34_H_25_ClN_8_OS 628.8; found 629.0952 [M + 1]^+^.

#### 4-(4-methoxyphenyl)-2-(2-((1-phenyl-3-(4-((1-phenyl-1H-1,2,3-triazol-4-yl)meth-oxy)-phenyl)-1H-pyrazol-4-yl)methylene)hydrazinyl)thiazole (12c, C_35_H_28_N_8_O_2_S)

White solid; yield: 84%; m.p.: 182–184 °C; ^1^H NMR (400 MHz, CDCl_3_) (ppm): 8.90 (s, 1H, CH=N), 8.55 (s, 1H, pyrazole), 8.12 (s, 1H, triazole), Ar–H [7.83–7.80 (m, 4H), 7.73–7.68 (m, 7H), 7.49–7.46 (m, 3H), 7.15 (dd, *J* = 5.6&3.6 Hz, 2H), 7.05–6.99 (m, 4H, thiazole)], 5.38 (s, 2H, CH_2_), 3.91 (s, 3H, CH_3_). ^13^C NMR (100 MHz, CDCl_3_, ppm): thiazole-C [168.82 (C), 151.28 (C), 114.29 (CH)], Ar–C [159.02 (C), 154.26 (C), 133.39 (C), 129.82 (CH), 129.65 (CH), 129.53 (CH), 128.95 (CH), 128.60 (CH), 127.9 (CH), 127.30 (CH), 127.0 (C), 121.09 (CH), 121.09 (CH), 120.69 (CH), 119.65 (CH), 119.28 (CH), 115.09 (CH),130.13 (CN), pyrazole-C [144.75 (C), 130.34 (CH), 114.97 (C)], triazole-C [144.75(C), 119.49, (CH)], 62.05 (CH_2_), 55.34 (CH_3_). IR: *v* = 3673, 3456, 2926, 1698, 1602, 1508, 1449, 1248, 1046 cm^−1^; Anal. Calcd. For C_35_H_28_N_8_O_2_S: C, 67.29; H, 4.52; N, 17.94; Found: C, 67.30; H, 4.50; N, 17.93. ESI–MS: *m/z* calcd for C_35_H_28_N_8_O_2_S 624; found 625.0 [M + 1]^+^.

#### 4-phenyl-2-(2-((1-phenyl-3-(4-((1-phenyl-1H-1,2,3-triazol-4-yl)methoxy)phenyl)-1H-pyrazol-4-yl)methylene)hydrazinyl)thiazole (12d, C_34_H_26_N_8_OS)

White solid; yield 80%; m.p.: 176–178 °C; ^1^H NMR (400 MHz, CDCl_3_) (ppm): 8.23 (s, 1H, CH = N), 8.08 (s, 1H, pyrazole), Ar–H [7.79 – 7.61 (m, 7H), 7.52–7.48 (m, 8H) 7.42–7.36 (m, 4H), 7.33 (d, *J* = 7.4 Hz, 1H), 7.09 (s, 1H), 6.83 (s, 1H, thiazole)], 5.37 (s, 2H, CH_2_).^13^C NMR (100 MHz, CDCl_3_, ppm): thiazole-C [169.0 (C), 151.6 (C), 103.8 (CH)], Ar–C [158.4 (C), 139.4 (C), 136.9 (C), 135.3 (C), 133.4 (CH), 131.7 (CH), 129.8 (CH), 129.5 (CH), 128.9, (CH), 127.5 (CH), 127.0 (CH), 126.3 (CH), 125.6 (C), 121.0 (CH), 120.6 (CH), pyrazole-C [144.8 (C), 130.0 (C), 114.9 (CH)], triazole-C [139.4 (C), 119.3 (CH)]. 62.1 (CH_2_). IR: *v* = 367, 3444, 2926, 1698, 1602, 1508, 1449, 1248, 1037 cm^−1^; Anal.Calcd. For C_34_H_26_N_8_OS: C, 68.67; H, 4.41; N, 18.84; Found: C, 68.65; H, 4.40; N, 18.83. ESI–MS: *m/z*calcd for C_34_H_26_N_8_OS 594.7; found 595.1578 [M + 1]^+^.

#### 4-(4-chlorophenyl)-2-(2-((3-(4-((1-(3-chlorophenyl)-1H-1,2,3-triazol-4-yl)methoxy) phenyl)-1-phenyl-1H-pyrazol-4-yl)methylene)hydrazinyl)thiazole (12e, C_34_H_24_Cl_2_N_8_OS)

White solid; yield: 84%; m.p.: 196–198 °C; ^1^H NMR (400 MHz, CDCl_3_) (ppm): 8.26 (s, 1H, CH=N), 8.08 (s, 1H, pyrazole),7.73 (s, 1H, triazole), Ar–H [7.81–7.77 (m, 3H), 7.66 (t, *J* = 7.5 Hz, 4H), 7.59 (d, *J* = 8.4 Hz, 2H), 7.50–7.43 (m, 5H), 7.30–7.26 (m, 4H), 7.08 (d, *J* = 8.4 Hz, 1H)], 6.82 (s, 1H, thiazole), 5.36 (s, 2H, CH_2_). ^13^C NMR (100 MHz, CDCl_3_, ppm): thiazole-C [176.3 (C), 151.9 (C), 109.9 (CH)], Ar–C [163.7 (C), 152.6 (C),140.0 (C), 136.9 (C), 134.5 (C), 130.8 (C), 127.1 (C),130.3 (C), 130.1 (CH), 129.8 (CH), 129.5 (CH), 129.4 (CH), 129.1 (CH), 129.1 (CH), 128.9 (CH), 128.7 (CH), 127.3 (CH), 121.0 (CH), 120.9 (CH), 120.6 (CH), 119.7 (CH), 119.3 (CH), 115.1 (CH)], pyrazole-C [158.5 (C), 130.0 (CH), 114.9 (C)], triazole-C [144.7 (C), 119.2 (CH)], 62.0 (CH_2_]. IR: *v* = 3673, 3437, 2932, 1692, 1581, 1495, 1235, 1045 cm^−1^; Anal.Calcd. for C_34_H_24_N_8_OSCl_2_: C, 61.54; H, 3.65; N, 16.89; Found: C, 61.53; H, 3.63; N, 16.88.ESI–MS: *m/z* calcd for C_34_H_24_N_8_OSCl_2_ 662.6; found 663.0325 [M + 1]^+^.

#### 4-(4-bromophenyl)-2-(2-((3-(4-((1-(3-chlorophenyl)-1H-1,2,3-triazol-4-yl)methoxy)-phenyl)-1-phenyl-1H-pyrazol-4-yl)methylene)hydrazinyl)thiazole (12f, C_34_H_24_BrClN_8_OS)

White solid; yield 83%; m.p.: 192–194 °C; ^1^H NMR (400 MHz, CDCl_3_) (ppm): 8.29 (s, 1H, CH=N), 8.08 (s, 1H, pyrazole), Ar–H [7.80 (dd, *J* = 4.2, 1.7 Hz, 2H), 7.78 (s, 1H, triazole), 7.72 (d, *J* = 8.2 Hz, 1H), 7.69 (d, *J* = 1.9 Hz, 1H), 7.64 (d, *J* = 4.1 Hz, 2H), 7.61 (dd, *J* = 5.8 & 3.8 Hz, 2H), 7.55–7.52 (m, 1H), 7.49–7.45 (m, 6H), 7.36 (dd, *J* = 4.8 & 3.1 Hz, 1H), 7.10 (d, *J* = 6.4 Hz, 1H)], 6.82 (s, 1H, thiazole), 5.36 (s, 2H, CH_2_). ^13^C NMR (100 MHz, CDCl_3_, ppm): thiazole-C [173.5 (C), 145.1 (C), 103.6 (CH)], Ar–C [168.9 (C), 136.3 (C), 136.0 (C), 135.6 (C), 131.8 (C), 130.1 (CH), 130.0 (CH), 129.5 (CH), 129.2 (CH), 129.0 (CH), 128.7 (CH), 127.5 (CH), 127.0 (CH), 126.5 (CH), 125.6 (C), 124.2, 123.2 (C), 123.0 (CH), 120.8 (CH), 118.5 (CH), 116.4 (CH), pyrazole-C [158.3 (C), 130.9 (CH), 114.9 (CH)], triazole-C [144.0 (C), 119.3 (CH)], 62.0 (CH_2_). IR: *v* = 3664, 3452, 2932, 1690, 1602, 1599, 1451, 1246, 1033 cm^−1^; Anal. Calcd. For C_34_H_24_N_8_OSBrCl: C, 57.68; H, 3.42; N, 15.83; Found: C, 57.67; H, 3.40; N, 15.82. ESI–MS: *m/z* calcd for C_34_H_24_N_8_OSBrCl 708.1; found 709.10 [M + 1]^+^.

#### 2-(2-((3-(4-((1-(3-chlorophenyl)-1H-1,2,3-triazol-4-yl)methoxy)phenyl)-1-phenyl-1H-pyrazol-4-yl)methylene)hydrazinyl)-4-(4-methoxyphenyl)thiazole (12 g, C_35_H_27_ClN8O_2_S)

White solid; yield 82%; m.p.: 186–188 °C; ^1^H NMR (400 MHz, CDCl_3_) (ppm): 8.89 (s, 1H, CH=N), 8.54 (s, 1H, pyrazole), Ar–H [8.11–8.10 (m, 1H), 7.83–7.79 (m, 4H), 7.73 (m, 6H), 7.49–7.46 (m, 3H), 7.15 (dd, *J* = 5.6 & 3.6 Hz, 2H), 7.04–6.96 (m, 4)], 5.39 (s, 2H, CH_2_), 3.92 (s, 3H, CH_3_).^13^C NMR (100 MHz, CDCl_3_, ppm): thiazole-C [171.42, 148.72, 111.09 (CH_2_)], Ar–C [163.11 (C), 160.27 (C), 129.83 (C), 129.66 (C), 129.53 (C), 129.01 (CH), 127.20 (CH), 126.93 (CH), 126.88 (CH), 125.71 (CH), 124.87 (CH), 120.88 (CH), 118.58 (CH), 116.20 (CH), 115.00 (CH), 114.23 (CH), 113.99 (CH), pyrazole-C [156.49 (C), 130.91 (CH), 114.80 (C)], triazole-C [140.07 (C), 119.32 (CH)], 61.98 (CH_2_), 55.34 (CH_3_). IR: *v* = 3664, 3452, 2932, 1690, 1599, 1503, 1451, 1246, 1033 cm^−1^; Anal. Calcd. For C_35_H_27_N_8_O_2_SCl: C, 63.77; H, 4.13; N, 17.00; Found: C, 63.76; H, 4.14; N, 17.01. ESI–MS: *m/z* calcd for C_35_H_27_N_8_O_2_SCl 658.05; found 659.0424 [M + 1]^+^.

#### 2-(2-((3-(4-((1-(3-chlorophenyl)-1H-1,2,3-triazol-4-yl)methoxy)phenyl)-1-phenyl-1H-pyrazol-4-yl)methylene)hydrazinyl)-4-phenylthiazole (12 h, C_34_H_25_ClN_8_OS)

Yield: 85%,white solid; m.p.: 180–182 °C; ^1^H NMR (400 MHz, CDCl_3_) (ppm): 8.29 (s, 1H, CH=N), 8.08 (s, 1H, pyrazole), 7.68 (s, 1H, triazole), Ar–H [7.82–7.76 (m, 6H), 7.60 (d, *J* = 8.7 Hz, 2H), 7.53 – 7.42 (m, 5H), 7.39–7.28 (m, 4H), 7.10 (d, *J* = 8.8 Hz, 2H)], 6.86 (s, 1H, thiazole), 5.38 (s, 2H, CH_2_). ^13^C NMR (100 MHz, CDCl_3_, ppm): thiazole-C [169.08 (C), 151.79 (C), 103.36(CH)], Ar–C [158.38 (C), 137.80 (C), 135.60 (C), 134.40 (C), 129.96 (C), 129.52 (C), 129.02 (CH), 128.75 (CH), 127.98 (CH), 127.00 (CH), 126.47 (CH), 125.97 (CH), 120.89 (CH), 118.58 (CH), 116.60 (CH), pyrazole-C [145.32 (C), 130.91 (C), 114.95 (CH)], triazole-C [139.60 (C), 119.24 (CH)], 62.04 (CH_2_). IR: *v* = 3673, 3437, 2932, 1581, 1495, 1446, 1235, 1045 cm^−1^; Anal. Calcd. For C_34_H_25_N_8_OSCl: C, 64.91; H, 4.01; N, 17.81; Found: C, 64.90; H, 4.00; N, 17.82.ESI–MS: *m/z* calcd for C_34_H_25_N_8_OSCl 628.2; found 629.1371 [M + 1]^+^.

#### 2-(2-((3-(4-((1-(3,4-dimethylphenyl)-1H-1,2,3-triazol-4-yl)methoxy)phenyl)-1-phenyl-1H-pyrazol-4-yl)methylene)hydrazinyl)-4-(4-methoxyphenyl)thiazole (12i, C_37_H_32_N_8_O_2_S)

White solid; yield 82%; m.p.: 170–172 °C; ^1^H NMR (400 MHz, CDCl_3_) (ppm): 8.31(s, 1H, CH=N), 8.02 (s, 1H, pyrazole), 7.43 (s, 1H, thiazole), Ar–H [7.80 (t, *J* = 7.4 Hz, 3H), 7.74–7.68 (m, 3H), 7.61 (s, 2H), 7.56–7.46 (m, 4H), 7.32 (dd, *J* = 14.2 & 7.7 Hz, 3H), 7.12 (d, *J* = 8.1 Hz, 2H)], 6.84 (s, 1H, thiazole), 5.37 (s, 2H, CH_2_), 3.80 (s, 3H, OCH_3_), 2.33 (s, 3H, CH_3_), 2.31 (s, 3H, CH_3_). ^13^C NMR (100 MHz, CDCl_3_, ppm): thiazole-C [164.4 (C), 151.8 (C), 114.9 (CH)], pyrazole-C [142.8 (C), 138.4 (C),117.9 (CH)], triazole-C [139.5 (C), 119.2 (CH)], Ar–H [161.9 (C), 158.4 (C), 137.7 (C), 134.8 (C), 133.5 (C), 130.9 (C), 130.6 (C), 130.0 (C), 129.5 (CH), 128.8 (C), 127.2 (C), 127.0 (C), 121.9 (C), 61.6 (CH_2_), 55.8 (OCH_3_), 19.9 (CH_3_), 19.5 (CH_3_). IR: *v* = 367, 3436, 2926, 1691, 1602, 1509, 1247, 1035 cm^−1^; Anal. Calcd. For C_37_H_32_N_8_O_2_S: C, 68.08; H, 4.94; N, 17.17; Found: C, 68.05; H, 4.92; N, 17.15. ESI–MS: *m/z* calcd for C_37_H_32_N_8_O_2_S 652.2; found 653.1621 [M + 1]^+^.

#### 4-(4-chlorophenyl)-2-(2-((3-(4-((1-(3,4-dimethylphenyl)-1H-1,2,3-triazol-4-yl)methoxy)phenyl)-1-phenyl-1H-pyrazol-4-yl)methylene)hydrazinyl)thiazole(12j,C_36_H_29_ClN_8_OS)

White solid; yield 80%; m.p.: 174–176 °C; ^1^H NMR (400 MHz, CDCl_3_) (ppm): 8.32(s, 1H, CH = N), 8.05 (s, 1H, pyrazole), 7.42 (s, 1H, triazole), Ar–H [7.79 (t, *J* = 7.6 Hz, 3H), 7.74–7.68 (m, 3H), 7.62 (s, 2H), 7.55–7.47 (m, 4H), 7.33 (dd, *J* = 14.5 & 7.6 Hz, 3H), 7.12 (d, *J* = 8.1 Hz, 2H), 6.84 (s, 1H, thiazole), 5.37 (s, 2H, CH_2_), 2.34 (s, 3H, CH_3_), 2.32 (s, 3H, CH_3_).^13^C NMR (100 MHz, CDCl_3_, ppm): thiazole-C [161.9 (C), 151.8 (C), 114.9 (CH)], pyrazole-C [139.5 (C), 130.9 (CH), 117.9 (C)], triazole-C [138.4 (C), 119.2 (CH)], Ar–H [158.4 (C), 137.7 (C), 130.0 (C), 121.7 (C), 134.8 (C), 133.5 (C), 130.6 (C), 129.5 (C), 128.8 (CH), 128.8 (CH), 127.7 (CH), 127.2 (CH), 127.0 (CH), 126.3 (CH), 125.5 (CH), 61.6 (CH_2_), 19.9 (CH_3_), 19.5 (CH_3_). IR: *v* = 3435, 3074, 2928, 1721, 1602, 1578, 1510, 1448, 1236, 1048 cm^−1^; Anal. Calcd. For C_36_H_29_N_8_OSCl: C, 65.79; H, 4.45; N, 17.05; Found: C, 65.78; H, 4.16; N, 17.04. ESI–MS: *m/z* calcd for C_36_H_29_N_8_OSCl 656.1; found 657.1523 [M + 1]^+^.

#### 4-(4-bromophenyl)-2-(2-((3-(4-((1-(3,4-dimethylphenyl)-1H-1,2,3-triazol-4-yl)methoxy)phenyl)-1-phenyl-1H-pyrazol-4-yl)methylene)hydrazinyl)thiazole (12 k, C_36_H_29_BrN_8_OS)

White solid; yield 84%; m.p.: 164–166 °C; ^1^H NMR (400 MHz, CDCl_3_) (ppm): 8.34 (s, 1H, CH=N), 8.04 (s, 1H, pyrazole), 7.53 (s, 1H, triazole), Ar–H [7.80 (d, *J* = 8.0 Hz, 2H), 7.69–7.61 (m, 5H), 7.49 (dd, *J* = 7.8 & 5.3 Hz, 5H), 7.43 (d, *J* = 5.9 Hz, 2H), 7.14 (d, *J* = 8.7 Hz, 2H), 6.84 (s, 1H, thiazole), 5.30 (m, 2H, CH_2_), 2.35 (s, 3H, CH_3_), 2.32 (s, 3H, CH_3_)].^13^C NMR (100 MHz, CDCl_3_, ppm): thiazole-C [161.9 (C), 151.8 (C), 114.9 (CH)], pyrazole-C [138.4 (C),130.9 (CH), 117.9 (C)], triazole-C [146.8 (C), 119.2 (CH)], Ar–H [158.4 (C), 137.7 (C), 134.8 (C), 133.5 (C), 130.6 (C), 130.0 (C), 128.8 (C), 127.2 (CH), 127.0 (CH), 125.5 (CH), 121.7 (CH), 121.1 (CH)],61.6 (CH_2_), 19.9 (CH_3_), 19.5 (CH_3_). IR: v = 3535, 3435, 2928, 1578, 1448, 1236, 1048 cm^−1^; Anal. Calcd. For C_36_H_29_N_8_OSBr: C, 61.63; H, 4.17; N, 15.97; Found: C, 61.62; H, 4.18; N, 15.98.ESI–MS: *m/z* calcd for C_36_H_29_N_8_OSBr 700.0; found 701.0441 [M + 1]^+^.

#### 2-(2-((3-(4-((1-(3,4-dimethylphenyl)-1H-1,2,3-triazol-4-yl)methoxy)phenyl)-1-phenyl-1H-pyrazol-4-yl)methylene)hydrazinyl)-4-phenylthiazole (12 l,C_36_H_30_N_8_OS)

White solid; yield 82%; m.p.: 168–170 °C; ^1^H NMR (400 MHz, CDCl_3_) (ppm): 8.32 (s, 1H, CH=N), 8.03 (s, 1H, pyrazole), Ar–H [7.79 (d, *J* = 7.6 Hz, 2H), 7.75 (d, *J* = 8.1 Hz, 2H), 7.53 (s, 3H), 7.48 (d, *J* = 8.2 Hz, 3H), 7.43 (d, *J* = 8.5 Hz, 3H), 7.36 (s, 1H, triazole), 7.31 (d, *J* = 6.8 Hz, 2H), 7.15 – 7.11 (m, 3H), 6.81 (s, 1H, thiazole), 2.34 (s, 3H, CH_3_), 2.32 (s, 3H, CH_3_).^13^C NMR (100 MHz, CDCl_3_, ppm): thiazole-C [170.84 (C), 153.45 (C), 114.24 (CH)], Ar–C [162.04 (C), 138.42 (C), 137.73 (C), 134.91 (C), 134.64 (C), 133.74 (C), 133.39 (C), 130.32 (CH), 130.14 (CH), 129.78 (CH), 129.65 (CH), 129.49 (CH), 127.67 (CH), 127.21 (CH), 124.48 (CH), 121.84 (CH), 119.32 (CH), 117.98 (C), 115.10 (CH), 114.94 (CH)], pyrazole-C [148.11 (C), 130.68 (CH), 114.33 (C)], triazole-C [145.49 (C), 119.65 (CH)], 62.13 (CH_2_), 19.94 (CH_3_), 19.51 (CH_3_). IR: *v* = 3667, 3494, 3121, 2926, 1691, 1602, 1509, 1452, 1247, 1035 cm^−1^; Anal. Calcd. For C_36_H_30_N_8_OS: C, 69.43; H, 4.86; N, 17.99; Found: C, 69.43; H, 4.84; N, 17.98. ESI–MS: *m/z* calcd for C_36_H_30_N_8_OS 621.7; found 622.85 [M + 1]^+^.

### *Protocol for the evaluation of anti*microbial activity

All microbial cultures received were grown on Muller-Hinton Agar plates (20 ml/plate) at 37 °C for 24 h in sterile conditions and were treated as stock cultures. A loop full of the grown cultures was then inoculated into sterile autoclaved 10 mL Luria–Bertani Broth, Miller (LB) broth medium containing Tryptone (10 g/L), Yeast extract (5 g/L), Sodium chloride (10 g/L), pH 7.5, and kept aside in an incubator shaker for 12 h at 37 °C with continuous shaking at 220 rpm for growth. After exactly 12 h, growth was observed through the formation of turbidity in cultures and confirmed by comparison with the control which was not inoculated. Subsequently, 1% of the grown culture was re-inoculated into fresh new LB broth and incubated in the same conditions as above for another 3.5 to 4 h until it reached the log phase. The culture grew and entered into the log phase, which is very active in participating in any reactions, and was confirmed by an absorbance of OD 0.4 to 0.6 at 600 nM. Therefore, it can be recommended for testing antibacterial activity.

### Minimum inhibitory concentration (MIC) determination using Broth method

Luria–Bertani Broth, Miller (LB broth), a potent microbiological growth medium, was prepared and sterilized using an autoclave under aseptic conditions, with 121 °C/15 Lbs pressure for 30 min, and used for antibiotic susceptibility testing. The microbial strains to be tested were grown at 37 °C for 12 h with continuous shaking at 220 rpm and maintained in the lag phase by storing them at 4 °C. The compounds synthesized for testing antimicrobial activity were dissolved in DMSO to produce a concentration of 1 mg/ml, which was treated as a stock solution. Subsequently, different concentrations of the compound ranging from minimum to maximum were tested against bacterial culture growth to determine its MIC value. The antibacterial activity of the compounds was determined by treating various concentrations of the compounds with 1ul/ml log phase cultures, followed by incubation for 12 h at 37 degrees C with continuous shaking at 220 rpm. Ampicillin, a standard drug with a 1% stock concentration, was used as a positive control for the antibacterial activity, and a blank was prepared by adding 1ul/ml culture to the broth and maintained as a negative control. Each tube contained 2 ml of fresh LB broth and 2ul of log phase bacterial culture with varying concentrations of compounds to check its MIC. All tests were performed in triplicate to validate the observed results (Mean ± SEM) by measuring the variability of data.

$${\text{Standard}}\,{\text{Error of mean (SEM)}}\,{ = }\,{\text{STDEV(X}}_{{\text{i}}} \, - \,{\text{X}}_{{\text{n}}} )/\sqrt {({\text{COUNT}}({\text{X}}_{{\text{i}}} \, - \,{\text{X}}_{{\text{n}}} ))}$$

where Xi-Xn is the number of data point values in triplicates.

### Protocol for antifungal activity determination and methodology

The antifungal activities of the synthesized compounds were tested against filamentous fungi *Aspergillus niger* MTCC 404 and *Saccharomyces cerevisiae* MTCC 1344 yeast cultures. They were obtained from the Culture Collection Division at Department of Microbiology, Osmania University and are chosen based on the nature of active most frequent contaminant of food. The fungal cultures were grown on yeast extract agar (YEP) media containing chloramphenicol (50 μg/mL) for 2–4 days at 28 °C. After 2 days, the black spores and lawn of cultures were collected and stored in an aqueous solution of 40% (v/v) glycerol at − 80 °C. Antifungal assay can be done by cylinder plate or disc method and cross streak plate method, broth dilution method. Here in the present study we used cross steak plate method to identify the minimum concentration of compound required to inhibit the fungal growth.

YEP agar media (commonly used to assess susceptibility of diverse mutants) was prepared by mixing the bactopeptone, sodium chloride and maintained final pH 7 at 25 °C. Sterilization of media is carried at 121 °C/15 lbs pressure for 30 min, and cooled till room temperature and poured into sterile petriplates. Aqueous culture of stock fungal strains was spreaded evenly on plates and different diluted concentration of compounds was streaked on plate. The measured zone of clear area length (mm) on YEP agar plate determined the efficacy of compound for fungal growth inhibition. Experiments were performed in triplicates to represent the data in form of Mean ± SEM.

### Antioxidant activity (DPPH free radical scavenging activity)

The antioxidant activity of the test compounds was checked by transferring concentrations of the compound ranging from 10 µM to 200 µM into separate sterile Eppendorf tubes. Each concentration was performed in triplicate to avoid mean differences. Transfer 100 μl of freshly prepared DPPH reagent to an Eppendorf tube and add distilled water to make up the mixture volume up to 1 mL. Gently shake and vortex the mixture and keep it in the dark at room temperature for 30 min. Further, a blank for the test is prepared by adding various concentrations of diluent compounds and a constant volume of the reagent to make the reaction up to 1 mL. The absorbance of the test compounds was measured using a UV spectrophotometer at 517 nm. Percentage antioxidant activity calculation

$$\mathrm{\% Antioxidant\, activity}=\frac{\mathrm{Mean\, abs \,of \,Control}-\mathrm{Mean \,abs \,of \,sample}}{\mathrm{Mean \,abs \,of \,Control}} \mathrm{X} 100$$

### Molecular docking studies

Molecular docking studies were performed by using Vina in PyRx docking tool to predict the protein–ligand interactions at molecular level. The co-crystal structure of Gram positive *S. aureus* topoisomerase IV enzyme of catalytic domain in complex with novobiocin (PDB ID: 4URN) and the crystal structure of COVID-19 main protease (6LU7) were used as targets. Topoisomerase IV is a known efficient DNA decatenase, it plays a crucial role in separation of daughter chromosomes during DNA replication. Antibiotics like novobiocin and ampicillin acts upon bacteria by targeting this topoisomerase IV [44, 45]. Target proteins were downloaded from Protein Data Bank and prepared by Biovia discovery studio tool. The water molecules were removed and polar hydrogens were added to the protein. The ligand molecules and protein structures determined were imported into PyRx and converted into PDBQT format. The active sites of target molecules were identified from the existing ligand molecules in crystal structures. The docking simulations were performed after assigning grid box and grid centers in Vina wizard. The docking results were visualized by using Pymol and Biovia Discovery Studio Visulaizer.

## Conclusion

In summary, we have synthesized a new series of pyrazole, triazole, based 2,4-di substituted thiazole derivatives **12a-l.** The synthesized compounds **12a-l** was characterized by ^1^H NMR, ^13^C NMR, ESI-Mass, HRMS spectral data. The anti-microbial activities of these compounds were evaluated against various bacterial and fungal strains. The synthesized compounds **12a, 12f,** and **12 k** showed good activity against the tested antimicrobial, similarly these compounds have shown potent DPPH scavenging activity and it was emerged as potential molecules for further development. Moreover, the best dock scored compounds from our current in silico study were further evaluated for minimal microbial growth-inhibitory studies. The binding affinities of compounds 12a-l with topoisomerase IV enzyme ranged from-10.0 to − 11.0 kcal/mol, while COVID-19 main protease binding affinities ranged from − 8.2 to − 9.3 kcal/mol. These docking studies reveal that the compounds **12a-l** could be the best inhibitors for novel SARS Cov-2 virus and of more interested potent drug candidates.

## Supplementary Information

**Additional file 1: Fig. S1.** ^**1**^**H-NMR of **1-phenyl-3-(4-((1-phenyl-1H-1,2,3-triazol-4-yl)methoxy)phenyl)-1H-pyrazole-4-carbaldehyde (**8a**, C_25_H_19_N_5_O_2_). **Fig. S2. **^**13**^**C-NMR of **1-phenyl-3-(4-((1-phenyl-1H-1,2,3-triazol-4-yl)methoxy)phenyl)-1H-pyrazole-4-carbaldehyde (**8a**,C_25_H_19_N_5_O_2_). **Fig. S3. **^**1**^**H-NMR of** 3-(4-((1-(3-chlorophenyl)-1H-1,2,3-triazol-4-yl)methoxy)phenyl)-1-phenyl-1H-pyrazole-4-carbaldehyde (**8b, **C_25_H_18_ClN_5_O_2_). **Fig. S4. **^**13**^**C-NMR of** 3-(4-((1-(3-chlorophenyl)-1H-1,2,3-triazol-4-yl)methoxy)phenyl)-1-phenyl-1H-pyrazole-4-carbaldehyde (**8b, **C_25_H_18_ClN_5_O_2_). **Fig. S5. **^**1**^**H-NMR of** 3-(4-((1-(3,4-dimethylphenyl)-1H-1,2,3-triazol-4-yl)methoxy)phenyl)-1-phenyl-1H-pyrazole-4-carbaldehyde (**8c, **C_27_H_23_N_5_O_2_). **Fig. S6.** ^**13**^**C-NMR of** 3-(4-((1-(3,4-dimethylphenyl)-1H-1,2,3-triazol-4-yl)methoxy)phenyl)-1-phenyl-1H-pyrazole-4-carbaldehyde (**8c**, C_27_H_23_N_5_O_2_). **Fig. S7. **^**1**^**H-NMR of** 4-(4-bromophenyl)-2-(2-((1-phenyl-3-(4-((1-phenyl-1H-1,2,3-triazol-4-yl)meth-oxy)-phenyl)-1H-pyrazol-4-yl)methylene) hydrazinyl) thiazole **(12a**, C_34_H_25_BrN_8_OS). **Fig. S8. **^**13**^**C-NMR of** 4-(4-bromophenyl)-2-(2-((1-phenyl-3-(4-((1-phenyl-1H-1,2,3-triazol-4-yl)meth-oxy)-phenyl)-1H-pyrazol-4-yl)methylene) hydrazinyl) thiazole **(12a**, C_34_H_25_BrN_8_OS). **Fig. S9. FT-IR of** 4-(4-bromophenyl)-2-(2-((1-phenyl-3-(4-((1-phenyl-1H-1,2,3-triazol-4-yl)meth-oxy)-phenyl)-1H-pyrazol-4-yl)methylene) hydrazinyl) thiazole **(12a**, C_34_H_25_BrN_8_OS). **Fig. S10. Mass of** 4-(4-bromophenyl)-2-(2-((1-phenyl-3-(4-((1-phenyl-1H-1,2,3-triazol-4-yl)meth-oxy)-phenyl)-1H-pyrazol-4-yl)methylene) hydrazinyl) thiazole **(12a**, C_34_H_25_BrN_8_OS). **Fig. S11. **^**1**^**H-NMR of** 4-(4-chlorophenyl)-2-(2-((1-phenyl-3-(4-((1-phenyl-1H-1,2,3-triazol-4-yl)meth-oxy)-phenyl)-1H-pyrazol-4-yl)methylene)hydrazinyl) thiazole (**12b**, C_34_H_25_ClN_8_OS). **Fig. S12. **^**13**^**C-NMR of** 4-(4-chlorophenyl)-2-(2-((1-phenyl-3-(4-((1-phenyl-1H-1,2,3-triazol-4-yl)meth-oxy)-phenyl)-1H-pyrazol-4-yl)methylene)hydrazinyl) thiazole (**12b, **C_34_H_25_ClN_8_OS). **Fig. S13. FT-IR of** 4-(4-chlorophenyl)-2-(2-((1-phenyl-3-(4-((1-phenyl-1H-1,2,3-triazol-4-yl)meth-oxy)-phenyl)-1H-pyrazol-4-yl)methylene)hydrazinyl) thiazole (**12b**, C_34_H_25_ClN_8_OS). **Fig. S14. ESI-Mass of** 4-(4-chlorophenyl)-2-(2-((1-phenyl-3-(4-((1-phenyl-1H-1,2,3-triazol-4-yl)meth-oxy)-phenyl)-1H-pyrazol-4-yl)methylene)hydrazinyl) thiazole (**12b, **C_34_H_25_ClN_8_OS). **Fig. S15. **^**1**^**H-NMR of **4-(4-methoxyphenyl)-2-(2-((1-phenyl-3-(4-((1-phenyl-1H-1,2,3-triazol-4-yl)meth-oxy)-phenyl)-1H-pyrazol-4-yl)methylene)hydrazinyl) thiazole (**12c,**C_35_H_28_N_8_O_2_S). **Fig. S16. **^**13**^**C-NMR of** 4-(4-methoxyphenyl)-2-(2-((1-phenyl-3-(4-((1-phenyl-1H-1,2,3-triazol-4-yl)meth-oxy)-phenyl)-1H-pyrazol-4-yl)methylene)hydrazinyl) thiazole (**12c**,C_35_H_28_N_8_O_2_S). **Fig. S17. FT-IR of** 4-(4-methoxyphenyl)-2-(2-((1-phenyl-3-(4-((1-phenyl-1H-1,2,3-triazol-4-yl)meth-oxy)-phenyl)-1H-pyrazol-4-yl)methylene)hydrazinyl) thiazole (**12c,**C_35_H_28_N_8_O_2_S). **Fig. S18. ESI-Mass of** 4-(4-methoxyphenyl)-2-(2-((1-phenyl-3-(4-((1-phenyl-1H-1,2,3-triazol-4-yl)meth-oxy)-phenyl)-1H-pyrazol-4-yl)methylene) hydrazinyl)thiazole (**12c**,C_35_H_28_N_8_O_2_S). **Fig. S19. **^**1**^**H-NMR of** 4-phenyl-2-(2-((1-phenyl-3-(4-((1-phenyl-1H-1,2,3-triazol-4-yl)methoxy)phenyl)-1H-pyrazol-4-yl)methylene)hydrazinyl)thiazole (**12d**, C_34_H_26_N_8_OS). **Fig. S20. **^**13**^**C-NMR of** 4-phenyl-2-(2-((1-phenyl-3-(4-((1-phenyl-1H-1,2,3-triazol-4-yl)methoxy)phenyl)-1H-pyrazol-4-yl)methylene)hydrazinyl)thiazole (**12d**, C_34_H_26_N_8_OS). **Fig. S21. ESI-Mass of** 4-phenyl-2-(2-((1-phenyl-3-(4-((1-phenyl-1H-1,2,3-triazol-4-yl)methoxy)phenyl)-1H-pyrazol-4-yl)methylene)hydrazinyl)thiazole (**12d**, C_34_H_26_N_8_OS). **Fig. S22. ESI-Mass of** 4-phenyl-2-(2-((1-phenyl-3-(4-((1-phenyl-1H-1,2,3-triazol-4-yl)methoxy)phenyl)-1H-pyrazol-4-yl)methylene)hydrazinyl)thiazole (**12d**, C_34_H_26_N_8_OS). **Fig. S23. **^**1**^**H-NMR of** 4-(4-chlorophenyl)-2-(2-((3-(4-((1-(3-chlorophenyl)-1H-1,2,3-triazol-4-yl)methoxy)phenyl)-1-phenyl-1H-pyrazol-4-yl)methylene) hydrazinyl)thiazole (**12e, **C_34_H_24_Cl_2_N_8_OS). **Fig. S24. **^**13**^**C-NMR of **4-(4-chlorophenyl)-2-(2-((3-(4-((1-(3-chlorophenyl)-1H-1,2,3-triazol-4-yl)methoxy)phenyl)-1-phenyl-1H-pyrazol-4-yl)methylene) hydrazinyl)thiazole (**12e, **C_34_H_24_Cl_2_N_8_OS). **Fig. S25. FT-IR of** 4-(4-chlorophenyl)-2-(2-((3-(4-((1-(3-chlorophenyl)-1H-1,2,3-triazol-4-yl)methoxy)phenyl)-1-phenyl-1H-pyrazol-4-yl)methylene) hydrazinyl)thiazole (**12e, **C_34_H_24_Cl_2_N_8_OS). **Fig. S26. ESI-Mass of** 4-(4-chlorophenyl)-2-(2-((3-(4-((1-(3-chlorophenyl)-1H-1,2,3-triazol-4-yl)methoxy)phenyl)-1-phenyl-1H-pyrazol-4-yl)methylene) hydrazinyl)thiazole (**12e, **C_34_H_24_Cl_2_N_8_OS). **Fig. S27. **^**1**^**H-NMR of** 4-(4-bromophenyl)-2-(2-((3-(4-((1-(3-chlorophenyl)-1H-1,2,3-triazol-4-yl)methoxy)-phenyl)-1-phenyl-1H-pyrazol-4-yl)methylene) hydrazinyl)thiazole (**12f, **C_34_H_24_BrClN_8_OS). **Fig. S28. **^**13**^**C-NMR of** 4-(4-bromophenyl)-2-(2-((3-(4-((1-(3-chlorophenyl)-1H-1,2,3-triazol-4-yl)methoxy)-phenyl)-1-phenyl-1H-pyrazol-4-yl)methylene) hydrazinyl)thiazole (**12f, **C_34_H_24_BrClN_8_OS). **Fig. S29. FT-IR of** 4-(4-bromophenyl)-2-(2-((3-(4-((1-(3-chlorophenyl)-1H-1,2,3-triazol-4-yl)methoxy)-phenyl)-1-phenyl-1H-pyrazol-4-yl)methylene) hydrazinyl)thiazole (**12f**, C_34_H_24_BrClN_8_OS). **Fig. S30. ESI-Mass of** 4-(4-bromophenyl)-2-(2-((3-(4-((1-(3-chlorophenyl)-1H-1,2,3-triazol-4-yl)methoxy)-phenyl)-1-phenyl-1H-pyrazol-4-yl)methylene) hydrazinyl)thiazole (**12f**, C_34_H_24_BrClN_8_OS). **Fig. S31. **^**1**^**H-NMR of** 2-(2-((3-(4-((1-(3-chlorophenyl)-1H-1,2,3-triazol-4-yl)methoxy)phenyl)-1-phenyl-1H-pyrazol-4-yl)methylene)hydrazinyl)-4-(4-methoxyphenyl)thiazole(**12g**, C_35_H_27_ClN8O_2_S). **Fig. S32. **^**13**^**C-NMR of** 2-(2-((3-(4-((1-(3-chlorophenyl)-1H-1,2,3-triazol-4-yl)methoxy)phenyl)-1-phenyl-1H-pyrazol-4-yl)methylene)hydrazinyl)-4-(4-methoxyphenyl)thiazole(**12g**, C_35_H_27_ClN8O_2_S). **Fig. S33. FT-IR of** 2-(2-((3-(4-((1-(3-chlorophenyl)-1H-1,2,3-triazol-4-yl)methoxy)phenyl)-1-phenyl-1H-pyrazol-4-yl)methylene)hydrazinyl)-4-(4-methoxyphenyl)thiazole(**12g**, C_35_H_27_ClN8O_2_S). **Fig. S34. ESI-Mass of** 2-(2-((3-(4-((1-(3-chlorophenyl)-1H-1,2,3-triazol-4-yl)methoxy)phenyl)-1-phenyl-1H-pyrazol-4-yl)methylene)hydrazinyl)-4-(4-methoxyphenyl)thiazole(**12g**, C_35_H_27_ClN8O_2_S). **Fig. S35. **^**1**^**H-NMR of** 2-(2-((3-(4-((1-(3-chlorophenyl)-1H-1,2,3-triazol-4-yl)methoxy)phenyl)-1-phenyl-1H-pyrazol-4-yl)methylene)hydrazinyl)-4-phenylthiazole(**12h, **C_34_H_25_ClN_8_OS). **Fig. S36. **^**13**^**C-NMR of** 2-(2-((3-(4-((1-(3-chlorophenyl)-1H-1,2,3-triazol-4-yl)methoxy)phenyl)-1-phenyl-1H-pyrazol-4-yl)methylene)hydrazinyl)-4-phenylthiazole(**12h**, C_34_H_25_ClN_8_OS). **Fig. S37. FT-IR of** 2-(2-((3-(4-((1-(3-chlorophenyl)-1H-1,2,3-triazol-4-yl)methoxy)phenyl)-1-phenyl-1H-pyrazol-4-yl)methylene)hydrazinyl)-4-phenylthiazole(**12h**, C_34_H_25_ClN_8_OS). **Fig. S38. ESI-Mass of** 2-(2-((3-(4-((1-(3-chlorophenyl)-1H-1,2,3-triazol-4-yl)methoxy)phenyl)-1-phenyl-1H-pyrazol-4-yl)methylene)hydrazinyl)-4-phenylthiazole(**12h**, C_34_H_25_ClN_8_OS). **Fig. S39. **^**1**^**H-NMR of** 2-(2-((3-(4-((1-(3,4-dimethylphenyl)-1H-1,2,3-triazol-4-yl)methoxy)phenyl)-1-phenyl-1H-pyrazol-4-yl)methylene)hydrazinyl)-4-(4-methoxyphenyl)thiazole (**12i,** C_37_H_32_N_8_O_2_S). **Fig. S40. **^**13**^**C-NMR of** 2-(2-((3-(4-((1-(3,4-dimethylphenyl)-1H-1,2,3-triazol-4-yl)methoxy)phenyl)-1-phenyl-1H-pyrazol-4-yl)methylene)hydrazinyl)-4-(4-methoxyphenyl)thiazole (**12i**, C_37_H_32_N_8_O_2_S). **Fig. S41. FT-IR of** 2-(2-((3-(4-((1-(3,4-dimethylphenyl)-1H-1,2,3-triazol-4-yl)methoxy)phenyl)-1-phenyl-1H-pyrazol-4-yl)methylene)hydrazinyl)-4-(4-methoxyphenyl)thiazole (**12i**, C_37_H_32_N_8_O_2_S). **Fig. S42. ESI-Mass of** 2-(2-((3-(4-((1-(3,4-dimethylphenyl)-1H-1,2,3-triazol-4-yl)methoxy)phenyl)-1-phenyl-1H-pyrazol-4-yl)methylene)hydrazinyl)-4-(4-methoxyphenyl)thiazole (**12i**, C_37_H_32_N_8_O_2_S). **Fig. S43. **^**1**^**H-NMR of** 4-(4-chlorophenyl)-2-(2-((3-(4-((1-(3,4-dimethylphenyl)-1H-1,2,3-triazol-4-yl)methoxy)phenyl)-1-phenyl-1H-pyrazol-4-yl)methylene)hydrazinyl)thiazole(**12j**,C_36_H_29_ClN_8_OS). **Fig. S44. **^**13**^**C-NMR of** 4-(4-chlorophenyl)-2-(2-((3-(4-((1-(3,4-dimethylphenyl)-1H-1,2,3-triazol-4-yl)methoxy)phenyl)-1-phenyl-1H-pyrazol-4-yl)methylene)hydrazinyl)thiazole(**12j**,C_36_H_29_ClN_8_OS). **Fig. S45. FT-IR of** 4-(4-chlorophenyl)-2-(2-((3-(4-((1-(3,4-dimethylphenyl)-1H-1,2,3-triazol-4-yl)methoxy)phenyl)-1-phenyl-1H-pyrazol-4-yl)methylene)hydrazinyl)thiazole(**12j**,C_36_H_29_ClN_8_OS). Fig. S46. ESI-Mass of 4-(4-chlorophenyl)-2-(2-((3-(4-((1-(3,4-dimethylphenyl)-1H-1,2,3-triazol-4-yl)methoxy)phenyl)-1-phenyl-1H-pyrazol-4-yl)methylene)hydrazinyl)thiazole(**12j**,C_36_H_29_ClN_8_OS). **Fig. S47. **^**1**^**H-NMR of** 4-(4-bromophenyl)-2-(2-((3-(4-((1-(3,4-dimethylphenyl)-1H-1,2,3-triazol-4-yl)methoxy)phenyl)-1-phenyl-1H-pyrazol-4-yl)methylene)hydrazinyl)thiazole(**12k**, C_36_H_29_BrN_8_OS). **Fig. S48. **^**13**^**C-NMR of** 4-(4-bromophenyl)-2-(2-((3-(4-((1-(3,4-dimethylphenyl)-1H-1,2,3-triazol-4-yl)methoxy)phenyl)-1-phenyl-1H-pyrazol-4-yl)methylene)hydrazinyl)thiazole(**12k**, C_36_H_29_BrN_8_OS). **Fig. S49. FT-IR of** 4-(4-bromophenyl)-2-(2-((3-(4-((1-(3,4-dimethylphenyl)-1H-1,2,3-triazol-4-yl)methoxy)phenyl)-1-phenyl-1H-pyrazol-4-yl)methylene)hydrazinyl)thiazole(**12k**, C_36_H_29_BrN_8_OS). **Fig. S50. ESI-Mass of** 4-(4-bromophenyl)-2-(2-((3-(4-((1-(3,4-dimethylphenyl)-1H-1,2,3-triazol-4-yl)methoxy)phenyl)-1-phenyl-1H-pyrazol-4-yl)methylene)hydrazinyl)thiazole(**12k**, C_36_H_29_BrN_8_OS). **Fig. S51. **^**1**^**H-NMR of** 2-(2-((3-(4-((1-(3,4-dimethylphenyl)-1H-1,2,3-triazol-4-yl)methoxy)phenyl)-1-phenyl-1H-pyrazol-4-yl) methylene)hydrazinyl)-4-phenylthiazole(**12l**,C_36_H_30_N_8_OS). **Fig. S52. **^**13**^**C-NMR of** 2-(2-((3-(4-((1-(3,4-dimethylphenyl)-1H-1,2,3-triazol-4-yl)methoxy)phenyl)-1-phenyl-1H-pyrazol-4-yl) methylene)hydrazinyl)-4-phenylthiazole(**12l**,C_36_H_30_N_8_OS). **Fig. S53. FT-IR of** 2-(2-((3-(4-((1-(3,4-dimethylphenyl)-1H-1,2,3-triazol-4-yl)methoxy)phenyl)-1-phenyl-1H-pyrazol-4-yl) methylene)hydrazinyl)-4-phenylthiazole(**12l**,C_36_H_30_N_8_OS). **Fig. S54. ESI-Mass of** 2-(2-((3-(4-((1-(3,4-dimethylphenyl)-1H-1,2,3-triazol-4-yl)methoxy)phenyl)-1-phenyl-1H-pyrazol-4-yl) methylene)hydrazinyl)-4-phenylthiazole(**12l**,C_36_H_30_N_8_OS). **Fig. S55.** Zone of inhibition test for antimicrobial activity. **Table S1**. MIC of synthesized **12a-l**compounds (μg/ml) against gram-positive and gram-negative bacterial strains

## Data Availability

All data generated or analyzed during this study are included in this published article [and its Additional files] ^1^H-NMR,^13^C-NMR, FR-IR, and Mass spectral data of all new compounds 8a-c and 12a-l. Biological evaluation data.
