# Supplementary material for: Synthesis, antimicrobial and antioxidant activity of triazole, pyrazole containing thiazole derivatives and molecular docking studies on COVID-19
Source: BMC Chem. 2023 Jun 17;17(1):61. doi: 10.1186/s13065-023-00965-8 (PMC10276907; doi:10.1186/s13065-023-00965-8)
Supplement: Supplementary file 1 — Additional file 1: Fig. S1. 1H-NMR of 1-phenyl-3-(4-((1-phenyl-1H-1,2,3-triazol-4-yl)methoxy)phenyl)-1H-pyrazole-4-carbaldehyde (8a, C25H19N5O2). Fig. S2. 13C-NMR of 1-phenyl-3-(4-((1-phenyl-1H-1,2,3-triazol-4-yl)methoxy)phenyl)-1H-pyrazole-4-carbaldehyde (8a,C25H19N5O2). Fig. S3. 1H-NMR of 3-(4-((1-(3-chlorophenyl)-1H-1,2,3-triazol-4-yl)methoxy)phenyl)-1-phenyl-1H-pyrazole-4-carbaldehyde (8b, C25H18ClN5O2). Fig. S4. 13C-NMR of 3-(4-((1-(3-chlorophenyl)-1H-1,2,3-triazol-4-yl)methoxy)phenyl)-1-phenyl-1H-pyrazole-4-carbaldehyde (8b, C25H18ClN5O2). Fig. S5. 1H-NMR of 3-(4-((1-(3,4-dimethylphenyl)-1H-1,2,3-triazol-4-yl)methoxy)phenyl)-1-phenyl-1H-pyrazole-4-carbaldehyde (8c, C27H23N5O2). Fig. S6. 13C-NMR of 3-(4-((1-(3,4-dimethylphenyl)-1H-1,2,3-triazol-4-yl)methoxy)phenyl)-1-phenyl-1H-pyrazole-4-carbaldehyde (8c, C27H23N5O2). Fig. S7. 1H-NMR of 4-(4-bromophenyl)-2-(2-((1-phenyl-3-(4-((1-phenyl-1H-1,2,3-triazol-4-yl)meth-oxy)-phenyl)-1H-pyrazol-4-yl)methylene) hydrazinyl) thiazole (12a, C34H25BrN8OS). Fig. S8. 13C-NMR of 4-(4-bromophenyl)-2-(2-((1-phenyl-3-(4-((1-phenyl-1H-1,2,3-triazol-4-yl)meth-oxy)-phenyl)-1H-pyrazol-4-yl)methylene) hydrazinyl) thiazole (12a, C34H25BrN8OS). Fig. S9. FT-IR of 4-(4-bromophenyl)-2-(2-((1-phenyl-3-(4-((1-phenyl-1H-1,2,3-triazol-4-yl)meth-oxy)-phenyl)-1H-pyrazol-4-yl)methylene) hydrazinyl) thiazole (12a, C34H25BrN8OS). Fig. S10. Mass of 4-(4-bromophenyl)-2-(2-((1-phenyl-3-(4-((1-phenyl-1H-1,2,3-triazol-4-yl)meth-oxy)-phenyl)-1H-pyrazol-4-yl)methylene) hydrazinyl) thiazole (12a, C34H25BrN8OS). Fig. S11. 1H-NMR of 4-(4-chlorophenyl)-2-(2-((1-phenyl-3-(4-((1-phenyl-1H-1,2,3-triazol-4-yl)meth-oxy)-phenyl)-1H-pyrazol-4-yl)methylene)hydrazinyl) thiazole (12b, C34H25ClN8OS). Fig. S12. 13C-NMR of 4-(4-chlorophenyl)-2-(2-((1-phenyl-3-(4-((1-phenyl-1H-1,2,3-triazol-4-yl)meth-oxy)-phenyl)-1H-pyrazol-4-yl)methylene)hydrazinyl) thiazole (12b, C34H25ClN8OS). Fig. S13. FT-IR of 4-(4-chlorophenyl)-2-(2-((1-phenyl-3-(4-((1-phenyl-1H-1,2,3-triazol-4-yl)met [file 13065_2023_965_MOESM1_ESM.docx]

**Synthesis, antimicrobial and antioxidant activity of triazole, pyrazole containing thiazole derivatives and molecular docking studies on COVID-19**

Matta Raghavender^1^, Jalapathi Pochampally^1*^, Bala Narsimha Dhoddi^1^, Bhookya Shankar^2, 3^, Sampath Bitla^4^_,_ and Anjini Gayatri Akkiraju^5^

^1^Department of Chemistry, Osmania University, Hyderabad, Telangana, 500007, India

^2^School of Chemistry, University of Hyderabad, Central University, Telangana, 500046, India

^3^Department of Chemistry, Sreenidhi University, Hyderabad-501301, Telangana, India

^4^Department of Chemistry, Osmania University, Hyderabad, Telangana, 500001, India

^5^Molecular Medicine Lab, Department of Genetics & Biotechnology, Osmania University, Hyderabad, Telangana, 500007, India

Corresponding author Email: pochampalli.ou.chemi@gmail.com

**Figure legends**

**Fig. S1. ^1^H-NMR of** 1-phenyl-3-(4-((1-phenyl-1H-1,2,3-triazol-4-yl)methoxy)phenyl)-1H-pyrazole-4-carbaldehyde (**8a,** C_25_H_19_N_5_O_2_).

**Fig. S2. ^13^C-NMR of** 1-phenyl-3-(4-((1-phenyl-1H-1,2,3-triazol-4-yl)methoxy)phenyl)-1H-pyrazole-4-carbaldehyde (**8a,**C_25_H_19_N_5_O_2_).

**Fig. S3. ^1^H-NMR of** 3-(4-((1-(3-chlorophenyl)-1H-1,2,3-triazol-4-yl)methoxy)phenyl)-1-phenyl-1H-pyrazole-4-carbaldehyde (**8b,** C_25_H_18_ClN_5_O_2_).

**Fig. S4. ^13^C-NMR of** 3-(4-((1-(3-chlorophenyl)-1H-1,2,3-triazol-4-yl)methoxy)phenyl)-1-phenyl-1H-pyrazole-4-carbaldehyde (**8b,** C_25_H_18_ClN_5_O_2_).

**Fig. S5. ^1^H-NMR of** 3-(4-((1-(3,4-dimethylphenyl)-1H-1,2,3-triazol-4-yl)methoxy)phenyl)-1-phenyl-1H-pyrazole-4-carbaldehyde (**8c,** C_27_H_23_N_5_O_2_)

**Fig. S6. ^13^C-NMR of** 3-(4-((1-(3,4-dimethylphenyl)-1H-1,2,3-triazol-4-yl)methoxy)phenyl)-1-phenyl-1H-pyrazole-4-carbaldehyde (**8c,** C_27_H_23_N_5_O_2_).

**Fig. S7. ^1^H-NMR of** 4-(4-bromophenyl)-2-(2-((1-phenyl-3-(4-((1-phenyl-1H-1,2,3-triazol-4-yl)meth-oxy)-phenyl)-1H-pyrazol-4-yl)methylene) hydrazinyl) thiazole **(12a,** C_34_H_25_BrN_8_OS).

**Fig. S8. ^13^C-NMR of** 4-(4-bromophenyl)-2-(2-((1-phenyl-3-(4-((1-phenyl-1H-1,2,3-triazol-4-yl)meth-oxy)-phenyl)-1H-pyrazol-4-yl)methylene) hydrazinyl) thiazole **(12a,** C_34_H_25_BrN_8_OS).

**Fig. S9. FT-IR of** 4-(4-bromophenyl)-2-(2-((1-phenyl-3-(4-((1-phenyl-1H-1,2,3-triazol-4-yl)meth-oxy)-phenyl)-1H-pyrazol-4-yl)methylene) hydrazinyl) thiazole **(12a,** C_34_H_25_BrN_8_OS).

**Fig. S10. Mass of** 4-(4-bromophenyl)-2-(2-((1-phenyl-3-(4-((1-phenyl-1H-1,2,3-triazol-4-yl)meth-oxy)-phenyl)-1H-pyrazol-4-yl)methylene) hydrazinyl) thiazole **(12a,** C_34_H_25_BrN_8_OS).

**Fig. S11. ^1^H-NMR of** 4-(4-chlorophenyl)-2-(2-((1-phenyl-3-(4-((1-phenyl-1H-1,2,3-triazol-4-yl)meth-oxy)-phenyl)-1H-pyrazol-4-yl)methylene)hydrazinyl) thiazole (**12b,** C_34_H_25_ClN_8_OS)

**Fig. S12. ^13^C-NMR of** 4-(4-chlorophenyl)-2-(2-((1-phenyl-3-(4-((1-phenyl-1H-1,2,3-triazol-4-yl)meth-oxy)-phenyl)-1H-pyrazol-4-yl)methylene)hydrazinyl) thiazole (**12b,** C_34_H_25_ClN_8_OS).

**Fig. S13. FT-IR of** 4-(4-chlorophenyl)-2-(2-((1-phenyl-3-(4-((1-phenyl-1H-1,2,3-triazol-4-yl)meth-oxy)-phenyl)-1H-pyrazol-4-yl)methylene)hydrazinyl) thiazole (**12b,** C_34_H_25_ClN_8_OS).

**Fig. S14. ESI-Mass of** 4-(4-chlorophenyl)-2-(2-((1-phenyl-3-(4-((1-phenyl-1H-1,2,3-triazol-4-yl)meth-oxy)-phenyl)-1H-pyrazol-4-yl)methylene)hydrazinyl) thiazole (**12b,** C_34_H_25_ClN_8_OS).

**Fig. S15. ^1^H-NMR of** 4-(4-methoxyphenyl)-2-(2-((1-phenyl-3-(4-((1-phenyl-1H-1,2,3-triazol-4-yl)meth-oxy)-phenyl)-1H-pyrazol-4-yl)methylene)hydrazinyl) thiazole (**12c,**C_35_H_28_N_8_O_2_S).

**Fig. S16. ^13^C-NMR of** 4-(4-methoxyphenyl)-2-(2-((1-phenyl-3-(4-((1-phenyl-1H-1,2,3-triazol-4-yl)meth-oxy)-phenyl)-1H-pyrazol-4-yl)methylene)hydrazinyl) thiazole (**12c,**C_35_H_28_N_8_O_2_S)

**Fig. S17. FT-IR of** 4-(4-methoxyphenyl)-2-(2-((1-phenyl-3-(4-((1-phenyl-1H-1,2,3-triazol-4-yl)meth-oxy)-phenyl)-1H-pyrazol-4-yl)methylene)hydrazinyl) thiazole (**12c,**C_35_H_28_N_8_O_2_S).

**Fig. S18. ESI-Mass of** 4-(4-methoxyphenyl)-2-(2-((1-phenyl-3-(4-((1-phenyl-1H-1,2,3-triazol-4-yl)meth-oxy)-phenyl)-1H-pyrazol-4-yl)methylene) hydrazinyl)thiazole (**12c,**C_35_H_28_N_8_O_2_S).

**Fig. S19. ^1^H-NMR of** 4-phenyl-2-(2-((1-phenyl-3-(4-((1-phenyl-1H-1,2,3-triazol-4-yl)methoxy)phenyl)-1H-pyrazol-4-yl)methylene)hydrazinyl)thiazole (**12d,** C_34_H_26_N_8_OS).

**Fig. S20. ^13^C-NMR of** 4-phenyl-2-(2-((1-phenyl-3-(4-((1-phenyl-1H-1,2,3-triazol-4-yl)methoxy)phenyl)-1H-pyrazol-4-yl)methylene)hydrazinyl)thiazole (**12d,** C_34_H_26_N_8_OS).

**Fig. S21. ESI-Mass of** 4-phenyl-2-(2-((1-phenyl-3-(4-((1-phenyl-1H-1,2,3-triazol-4-yl)methoxy)phenyl)-1H-pyrazol-4-yl)methylene)hydrazinyl)thiazole (**12d,** C_34_H_26_N_8_OS).

**Fig. S22. ESI-Mass of** 4-phenyl-2-(2-((1-phenyl-3-(4-((1-phenyl-1H-1,2,3-triazol-4-yl)methoxy)phenyl)-1H-pyrazol-4-yl)methylene)hydrazinyl)thiazole (**12d,** C_34_H_26_N_8_OS).

**Fig. S23. ^1^H-NMR of** 4-(4-chlorophenyl)-2-(2-((3-(4-((1-(3-chlorophenyl)-1H-1,2,3-triazol-4-yl)methoxy)phenyl)-1-phenyl-1H-pyrazol-4-yl)methylene) hydrazinyl)thiazole (**12e,** C_34_H_24_Cl_2_N_8_OS)

**Fig. S24. ^13^C-NMR of** 4-(4-chlorophenyl)-2-(2-((3-(4-((1-(3-chlorophenyl)-1H-1,2,3-triazol-4-yl)methoxy)phenyl)-1-phenyl-1H-pyrazol-4-yl)methylene) hydrazinyl)thiazole (**12e,** C_34_H_24_Cl_2_N_8_OS)

**Fig. S25. FT-IR of** 4-(4-chlorophenyl)-2-(2-((3-(4-((1-(3-chlorophenyl)-1H-1,2,3-triazol-4-yl)methoxy)phenyl)-1-phenyl-1H-pyrazol-4-yl)methylene) hydrazinyl)thiazole (**12e,** C_34_H_24_Cl_2_N_8_OS)

**Fig. S26. ESI-Mass of** 4-(4-chlorophenyl)-2-(2-((3-(4-((1-(3-chlorophenyl)-1H-1,2,3-triazol-4-yl)methoxy)phenyl)-1-phenyl-1H-pyrazol-4-yl)methylene) hydrazinyl)thiazole (**12e,** C_34_H_24_Cl_2_N_8_OS)

**Fig. S27. ^1^H-NMR of** 4-(4-bromophenyl)-2-(2-((3-(4-((1-(3-chlorophenyl)-1H-1,2,3-triazol-4-yl)methoxy)-phenyl)-1-phenyl-1H-pyrazol-4-yl)methylene) hydrazinyl)thiazole (**12f,** C_34_H_24_BrClN_8_OS)

**Fig. S28. ^13^C-NMR of** 4-(4-bromophenyl)-2-(2-((3-(4-((1-(3-chlorophenyl)-1H-1,2,3-triazol-4-yl)methoxy)-phenyl)-1-phenyl-1H-pyrazol-4-yl)methylene) hydrazinyl)thiazole (**12f,** C_34_H_24_BrClN_8_OS)

**Fig. S29. FT-IR of** 4-(4-bromophenyl)-2-(2-((3-(4-((1-(3-chlorophenyl)-1H-1,2,3-triazol-4-yl)methoxy)-phenyl)-1-phenyl-1H-pyrazol-4-yl)methylene) hydrazinyl)thiazole (**12f,** C_34_H_24_BrClN_8_OS)

**Fig. S30. ESI-Mass of** 4-(4-bromophenyl)-2-(2-((3-(4-((1-(3-chlorophenyl)-1H-1,2,3-triazol-4-yl)methoxy)-phenyl)-1-phenyl-1H-pyrazol-4-yl)methylene) hydrazinyl)thiazole (**12f,** C_34_H_24_BrClN_8_OS).

**Fig. S31. ^1^H-NMR of** 2-(2-((3-(4-((1-(3-chlorophenyl)-1H-1,2,3-triazol-4-yl)methoxy)phenyl)-1-phenyl-1H-pyrazol-4-yl)methylene)hydrazinyl)-4-(4-methoxyphenyl)thiazole(**12g,** C_35_H_27_ClN8O_2_S**)**

**Fig. S32. ^13^C-NMR of** 2-(2-((3-(4-((1-(3-chlorophenyl)-1H-1,2,3-triazol-4-yl)methoxy)phenyl)-1-phenyl-1H-pyrazol-4-yl)methylene)hydrazinyl)-4-(4-methoxyphenyl)thiazole(**12g,** C_35_H_27_ClN8O_2_S**)**

**Fig. S33. FT-IR of** 2-(2-((3-(4-((1-(3-chlorophenyl)-1H-1,2,3-triazol-4-yl)methoxy)phenyl)-1-phenyl-1H-pyrazol-4-yl)methylene)hydrazinyl)-4-(4-methoxyphenyl)thiazole(**12g,** C_35_H_27_ClN8O_2_S**)**

**Fig. S34. ESI-Mass of** 2-(2-((3-(4-((1-(3-chlorophenyl)-1H-1,2,3-triazol-4-yl)methoxy)phenyl)-1-phenyl-1H-pyrazol-4-yl)methylene)hydrazinyl)-4-(4-methoxyphenyl)thiazole(**12g,** C_35_H_27_ClN8O_2_S**).**

**Fig. S35. ^1^H-NMR of** 2-(2-((3-(4-((1-(3-chlorophenyl)-1H-1,2,3-triazol-4-yl)methoxy)phenyl)-1-phenyl-1H-pyrazol-4-yl)methylene)hydrazinyl)-4-phenylthiazole(**12h,** C_34_H_25_ClN_8_OS).

**Fig. S36. ^13^C-NMR of** 2-(2-((3-(4-((1-(3-chlorophenyl)-1H-1,2,3-triazol-4-yl)methoxy)phenyl)-1-phenyl-1H-pyrazol-4-yl)methylene)hydrazinyl)-4-phenylthiazole(**12h,** C_34_H_25_ClN_8_OS).

**Fig. S37. FT-IR of** 2-(2-((3-(4-((1-(3-chlorophenyl)-1H-1,2,3-triazol-4-yl)methoxy)phenyl)-1-phenyl-1H-pyrazol-4-yl)methylene)hydrazinyl)-4-phenylthiazole(**12h,** C_34_H_25_ClN_8_OS).

**Fig. S38. ESI-Mass of** 2-(2-((3-(4-((1-(3-chlorophenyl)-1H-1,2,3-triazol-4-yl)methoxy)phenyl)-1-phenyl-1H-pyrazol-4-yl)methylene)hydrazinyl)-4-phenylthiazole(**12h,** C_34_H_25_ClN_8_OS).

**Fig. S39. ^1^H-NMR of** 2-(2-((3-(4-((1-(3,4-dimethylphenyl)-1H-1,2,3-triazol-4-yl)methoxy)phenyl)-1-phenyl-1H-pyrazol-4-yl)methylene)hydrazinyl)-4-(4-methoxyphenyl)thiazole (**12i,** C_37_H_32_N_8_O_2_S).

**Fig. S40. ^13^C-NMR of** 2-(2-((3-(4-((1-(3,4-dimethylphenyl)-1H-1,2,3-triazol-4-yl)methoxy)phenyl)-1-phenyl-1H-pyrazol-4-yl)methylene)hydrazinyl)-4-(4-methoxyphenyl)thiazole (**12i,** C_37_H_32_N_8_O_2_S).

**Fig. S41. FT-IR of** 2-(2-((3-(4-((1-(3,4-dimethylphenyl)-1H-1,2,3-triazol-4-yl)methoxy)phenyl)-1-phenyl-1H-pyrazol-4-yl)methylene)hydrazinyl)-4-(4-methoxyphenyl)thiazole (**12i,** C_37_H_32_N_8_O_2_S).

**Fig. S42. ESI-Mass of** 2-(2-((3-(4-((1-(3,4-dimethylphenyl)-1H-1,2,3-triazol-4-yl)methoxy)phenyl)-1-phenyl-1H-pyrazol-4-yl)methylene)hydrazinyl)-4-(4-methoxyphenyl)thiazole (**12i,** C_37_H_32_N_8_O_2_S).

**Fig. S43. ^1^H-NMR of** 4-(4-chlorophenyl)-2-(2-((3-(4-((1-(3,4-dimethylphenyl)-1H-1,2,3-triazol-4-yl)methoxy)phenyl)-1-phenyl-1H-pyrazol-4-yl)methylene)hydrazinyl)thiazole(**12j,**C_36_H_29_ClN_8_OS).

**Fig. S44. ^13^C-NMR of** 4-(4-chlorophenyl)-2-(2-((3-(4-((1-(3,4-dimethylphenyl)-1H-1,2,3-triazol-4-yl)methoxy)phenyl)-1-phenyl-1H-pyrazol-4-yl)methylene)hydrazinyl)thiazole(**12j,**C_36_H_29_ClN_8_OS).

**Fig. S45. FT-IR of** 4-(4-chlorophenyl)-2-(2-((3-(4-((1-(3,4-dimethylphenyl)-1H-1,2,3-triazol-4-yl)methoxy)phenyl)-1-phenyl-1H-pyrazol-4-yl)methylene)hydrazinyl)thiazole(**12j,**C_36_H_29_ClN_8_OS).

**Fig. S46. ESI-Mass of** 4-(4-chlorophenyl)-2-(2-((3-(4-((1-(3,4-dimethylphenyl)-1H-1,2,3-triazol-4-yl)methoxy)phenyl)-1-phenyl-1H-pyrazol-4-yl)methylene)hydrazinyl)thiazole(**12j,**C_36_H_29_ClN_8_OS).

**Fig. S47. ^1^H-NMR of** 4-(4-bromophenyl)-2-(2-((3-(4-((1-(3,4-dimethylphenyl)-1H-1,2,3-triazol-4-yl)methoxy)phenyl)-1-phenyl-1H-pyrazol-4-yl)methylene)hydrazinyl)thiazole(**12k,** C_36_H_29_BrN_8_OS**).**

**Fig. S48. ^13^C-NMR of** 4-(4-bromophenyl)-2-(2-((3-(4-((1-(3,4-dimethylphenyl)-1H-1,2,3-triazol-4-yl)methoxy)phenyl)-1-phenyl-1H-pyrazol-4-yl)methylene)hydrazinyl)thiazole(**12k,** C_36_H_29_BrN_8_OS**).**

**Fig. S49. FT-IR of** 4-(4-bromophenyl)-2-(2-((3-(4-((1-(3,4-dimethylphenyl)-1H-1,2,3-triazol-4-yl)methoxy)phenyl)-1-phenyl-1H-pyrazol-4-yl)methylene)hydrazinyl)thiazole(**12k,** C_36_H_29_BrN_8_OS**).**

**Fig. S50. ESI-Mass of** 4-(4-bromophenyl)-2-(2-((3-(4-((1-(3,4-dimethylphenyl)-1H-1,2,3-triazol-4-yl)methoxy)phenyl)-1-phenyl-1H-pyrazol-4-yl)methylene)hydrazinyl)thiazole(**12k,** C_36_H_29_BrN_8_OS**).**

**Fig. S51. ^1^H-NMR of** 2-(2-((3-(4-((1-(3,4-dimethylphenyl)-1H-1,2,3-triazol-4-yl)methoxy)phenyl)-1-phenyl-1H-pyrazol-4-yl) methylene)hydrazinyl)-4-phenylthiazole(**12l,**C_36_H_30_N_8_OS).

**Fig. S52. ^13^C-NMR of** 2-(2-((3-(4-((1-(3,4-dimethylphenyl)-1H-1,2,3-triazol-4-yl)methoxy)phenyl)-1-phenyl-1H-pyrazol-4-yl) methylene)hydrazinyl)-4-phenylthiazole(**12l,**C_36_H_30_N_8_OS).

**Fig. S53. FT-IR of** 2-(2-((3-(4-((1-(3,4-dimethylphenyl)-1H-1,2,3-triazol-4-yl)methoxy)phenyl)-1-phenyl-1H-pyrazol-4-yl) methylene)hydrazinyl)-4-phenylthiazole(**12l,**C_36_H_30_N_8_OS).

**Fig. S54. ESI-Mass of** 2-(2-((3-(4-((1-(3,4-dimethylphenyl)-1H-1,2,3-triazol-4-yl)methoxy)phenyl)-1-phenyl-1H-pyrazol-4-yl) methylene)hydrazinyl)-4-phenylthiazole(**12l,**C_36_H_30_N_8_OS).

**Biological Evaluation Raw Data**

**Docking Raw Data**

**NMR spectral FIDs**


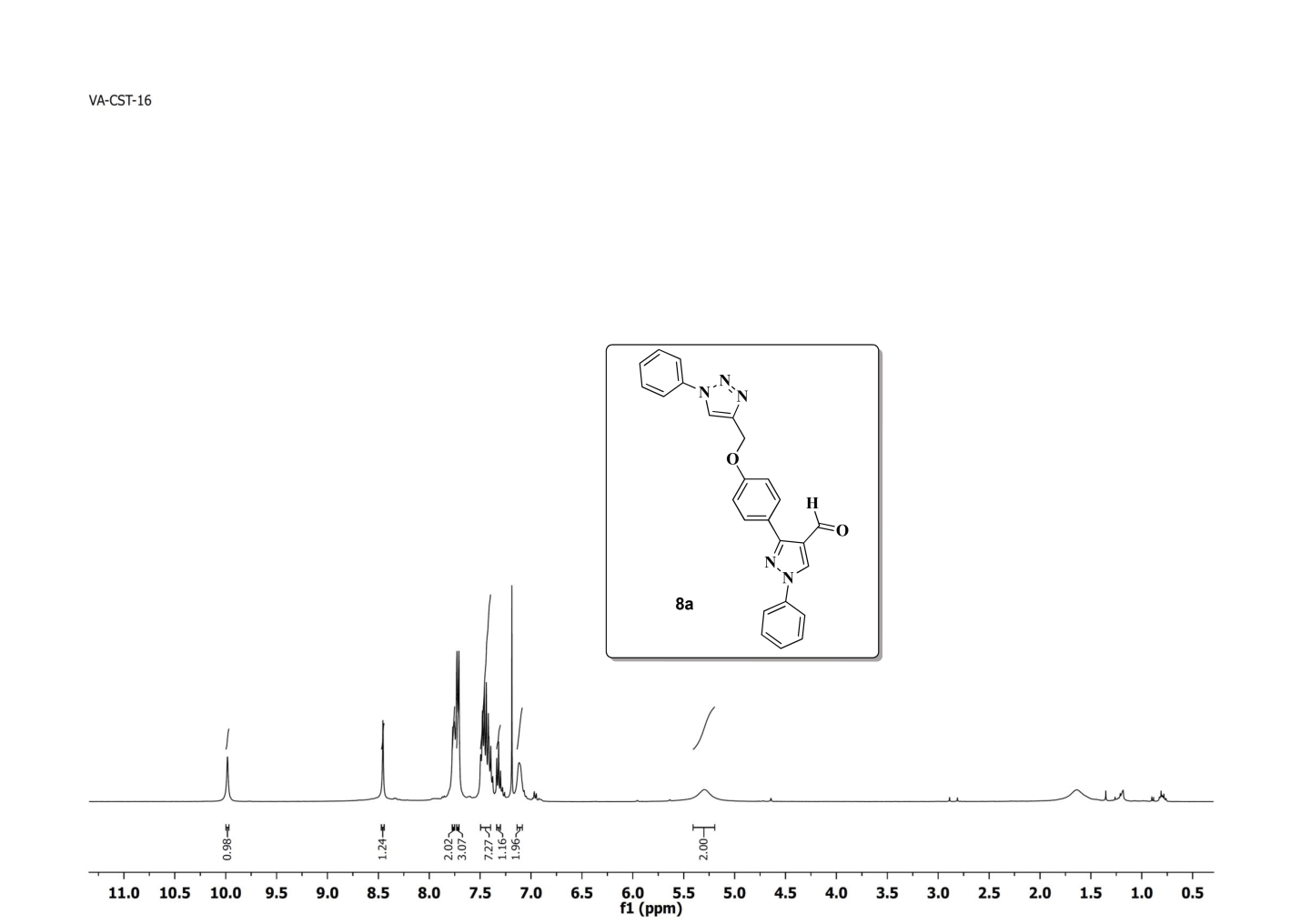
**Fig. S1. ^1^H NMR of 1-phenyl-3-(4-((1-phenyl-1H-1,2,3-triazol-4-yl)methoxy)phenyl)-1H-pyrazole-4-carbaldehyde** **8a (400 MHz, CDCl_3_)**


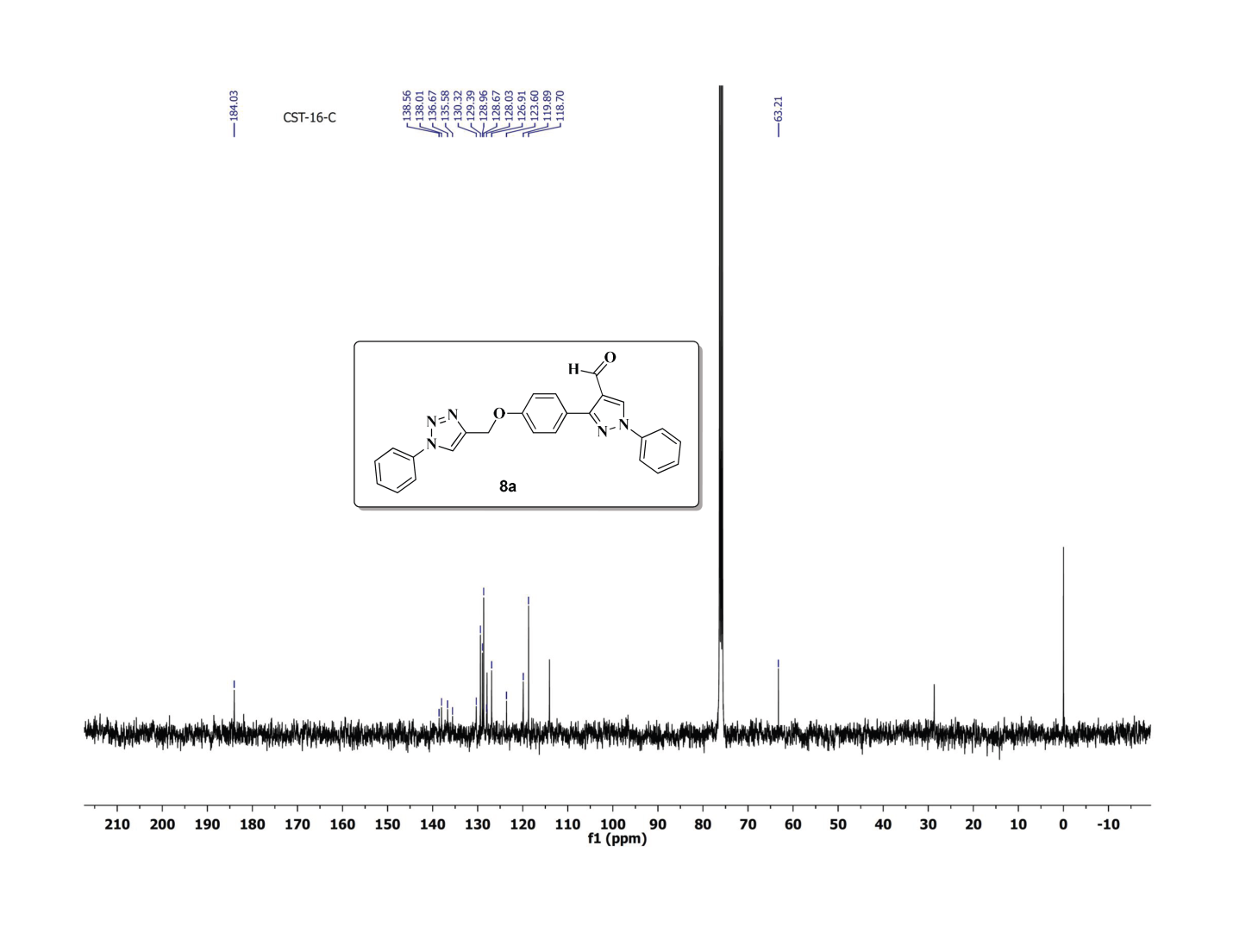


**Fig. S2. ^13^C NMR of 1-phenyl-3-(4-((1-phenyl-1H-1,2,3-triazol-4-yl)methoxy)phenyl)-1H-pyrazole-4-carbaldehyde** **8a (100 MHz, CDCl_3_)**

**^
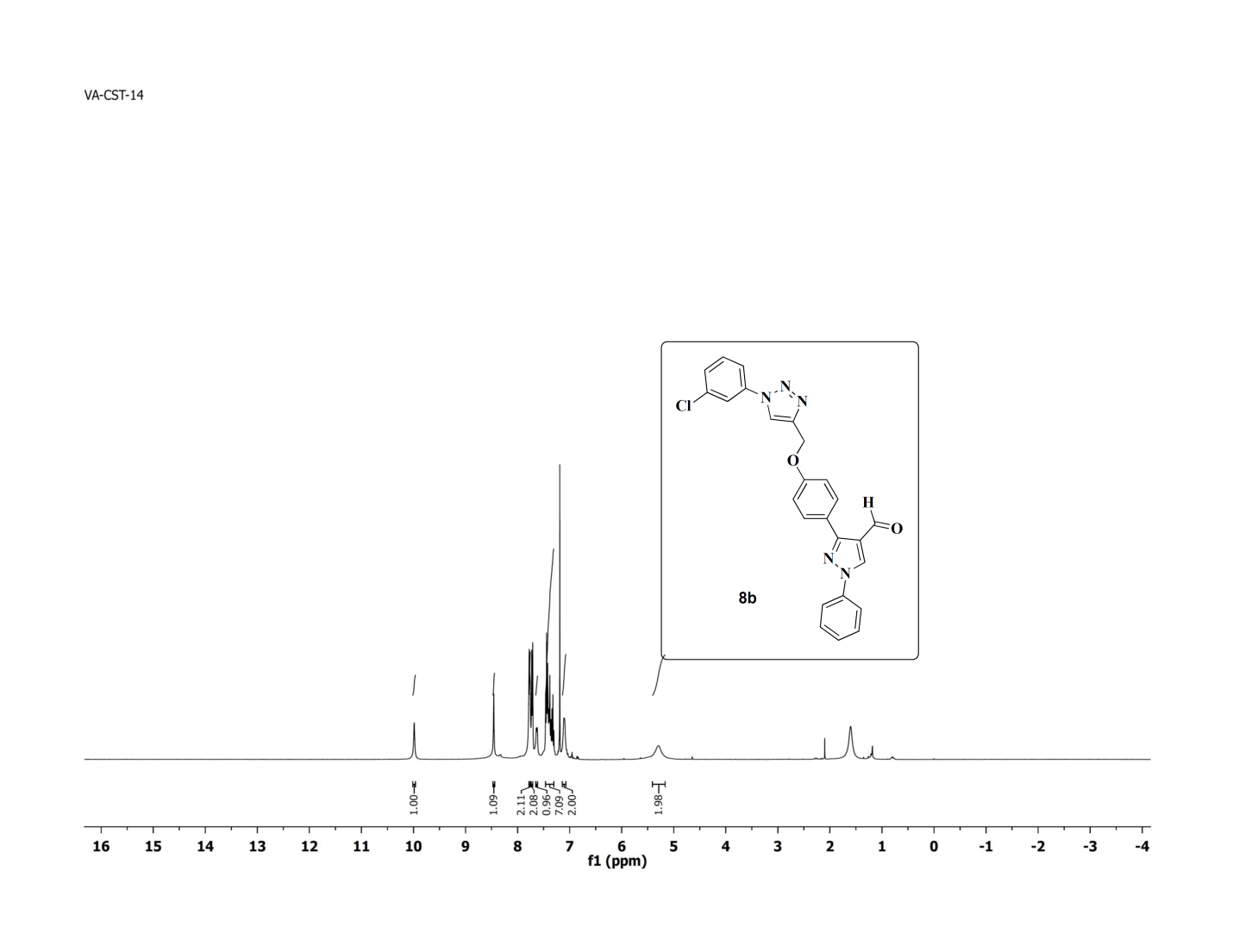
^**

**Fig. S3. ^1^HNMR of 3-(4-((1-(3-chlorophenyl)-1H-1,2,3-triazol-4-yl)methoxy)phenyl)-1-phenyl-1H-pyrazole-4-carbaldehyde 8b (400 MHz, CDCl_3_)**

**
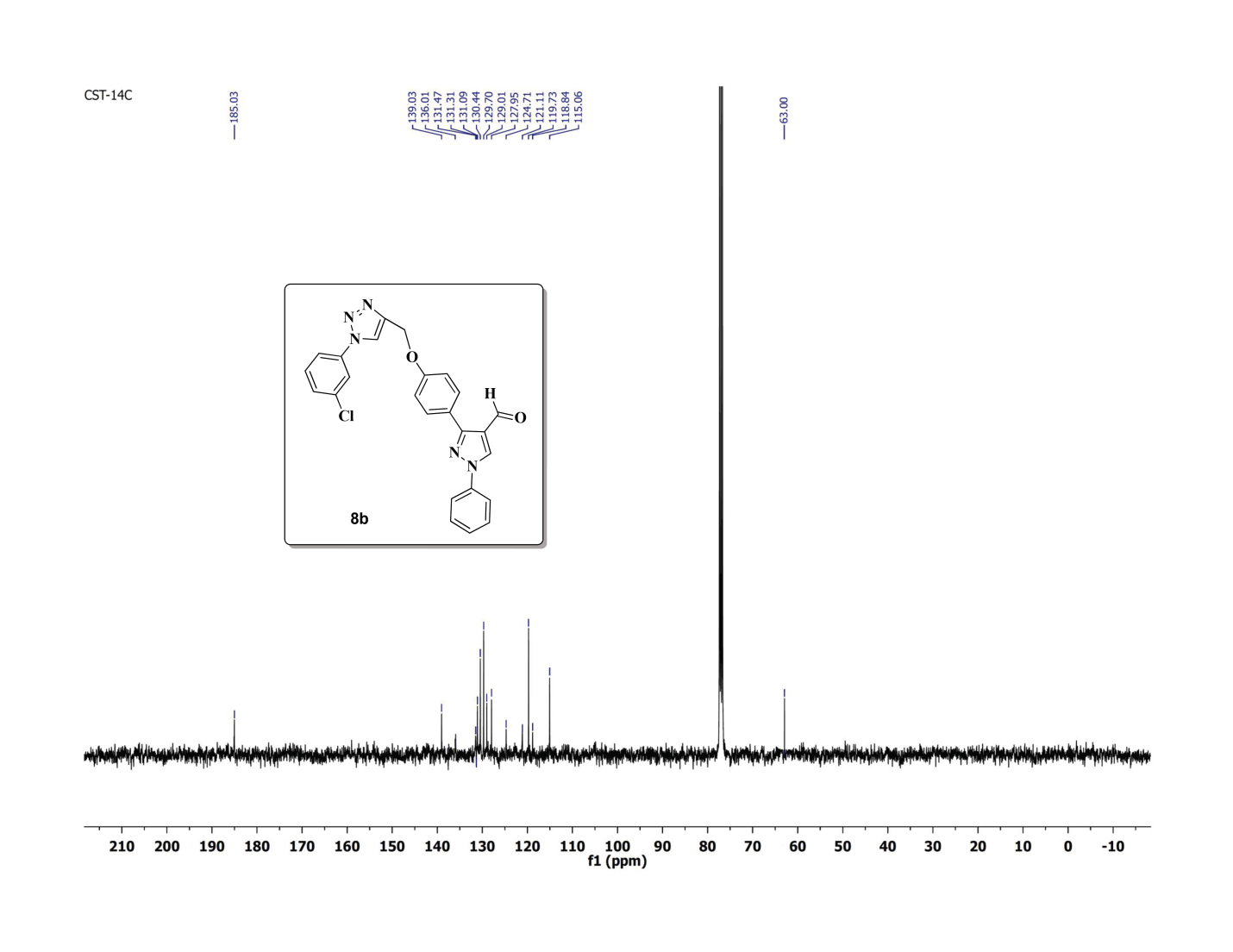
**

**Fig. S4. ^13^C-NMR of 3-(4-((1-(3-chlorophenyl)-1H-1,2,3-triazol-4-yl)methoxy)phenyl)-1-phenyl-1H-pyrazole-4-carbaldehyde** **8b (100 MHz, CDCl_3_)**

**
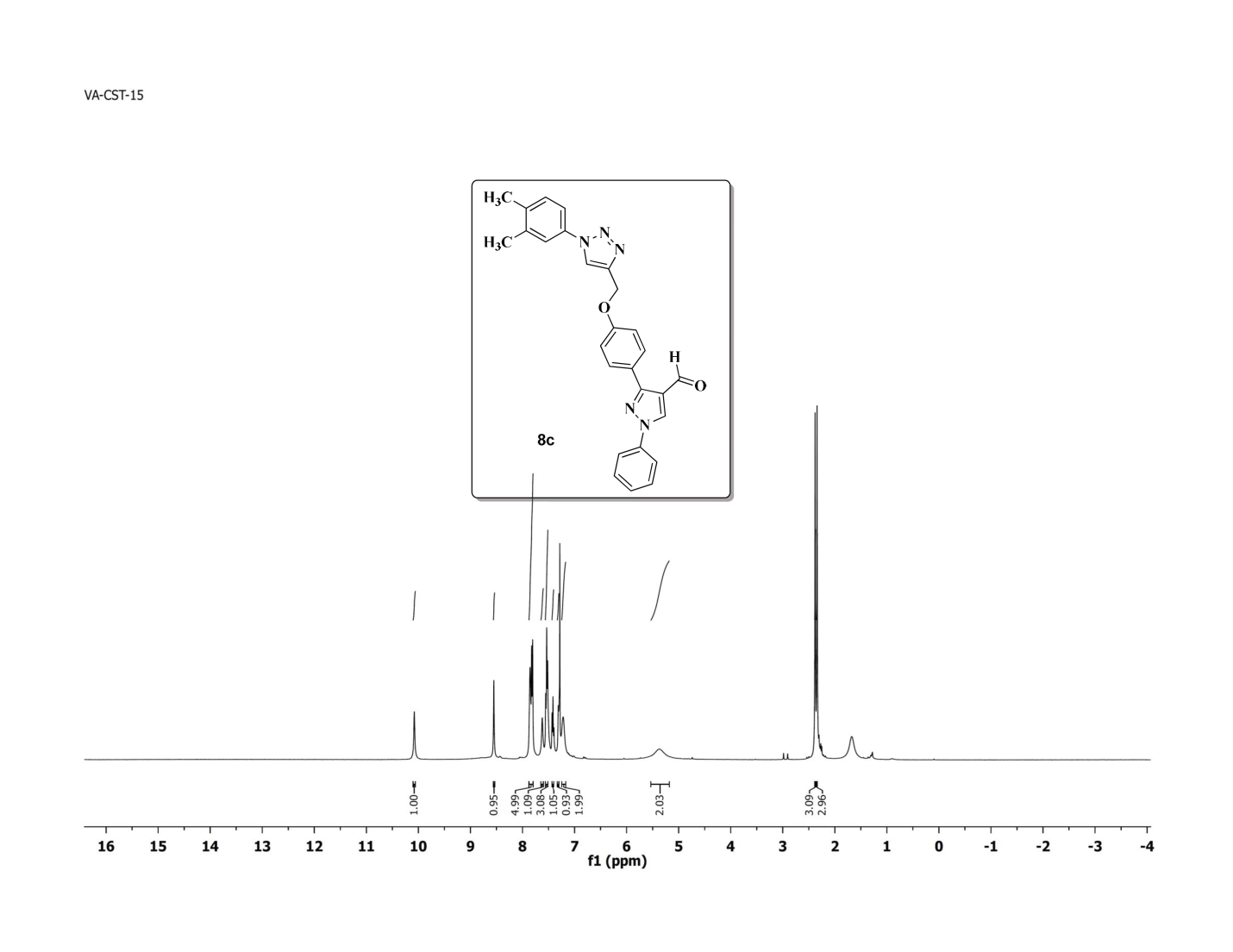
Fig. S5. ^1^H-NMR of 3-(4-((1-(3,4-dimethylphenyl)-1H-1,2,3-triazol-4-yl)methoxy)phenyl)-1-phenyl-1H-pyrazole-4-carbaldehyde 8c (400 MHz, CDl_3_)**


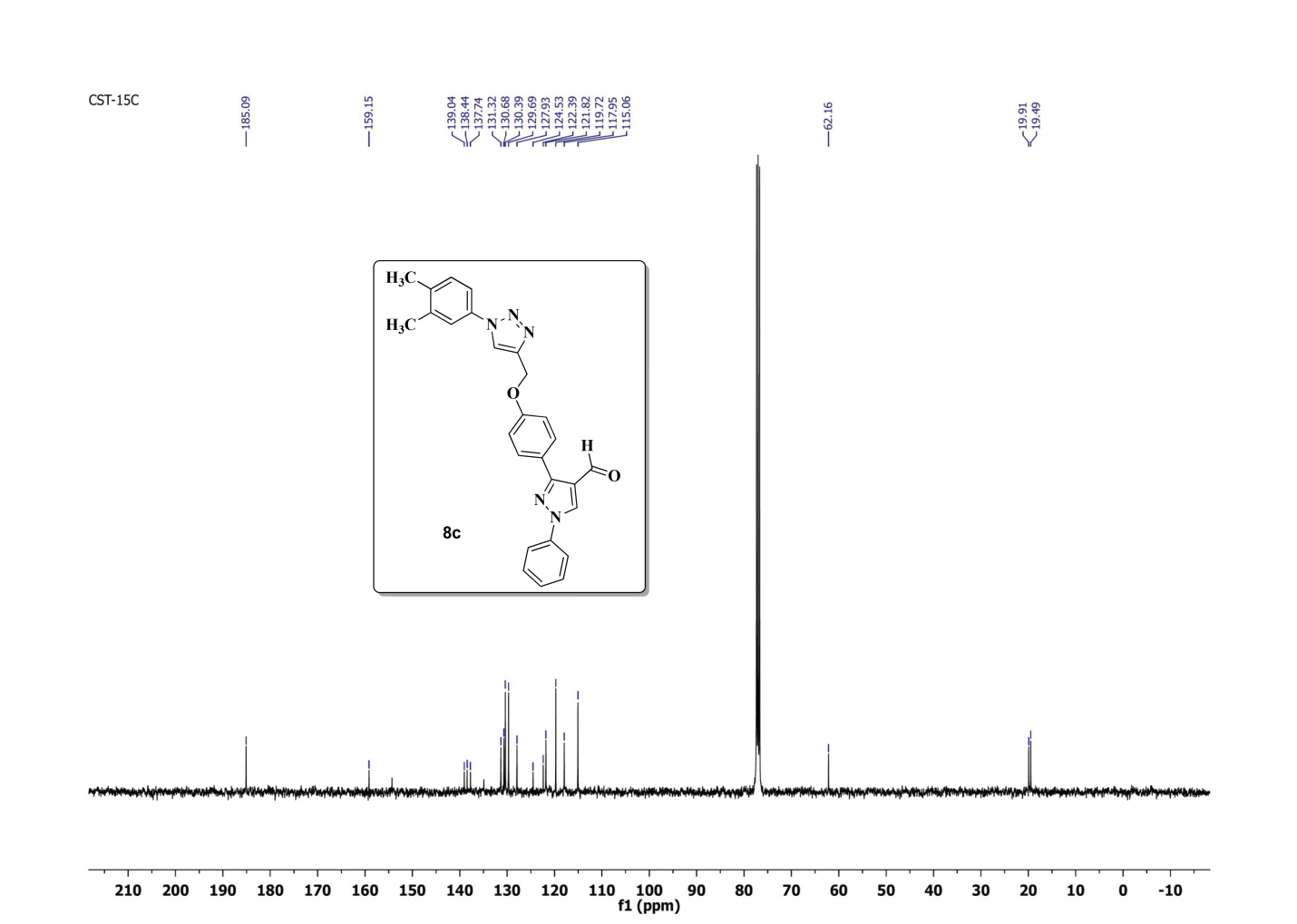
 **Fig. S6. ^13^C NMR of 3-(4-((1-(3,4-dimethylphenyl)-1H-1,2,3-triazol-4-yl)methoxy)phenyl)-1-phenyl-1H-pyrazole-4-carbaldehyde 8c (100 MHz, CDCl_3_)**


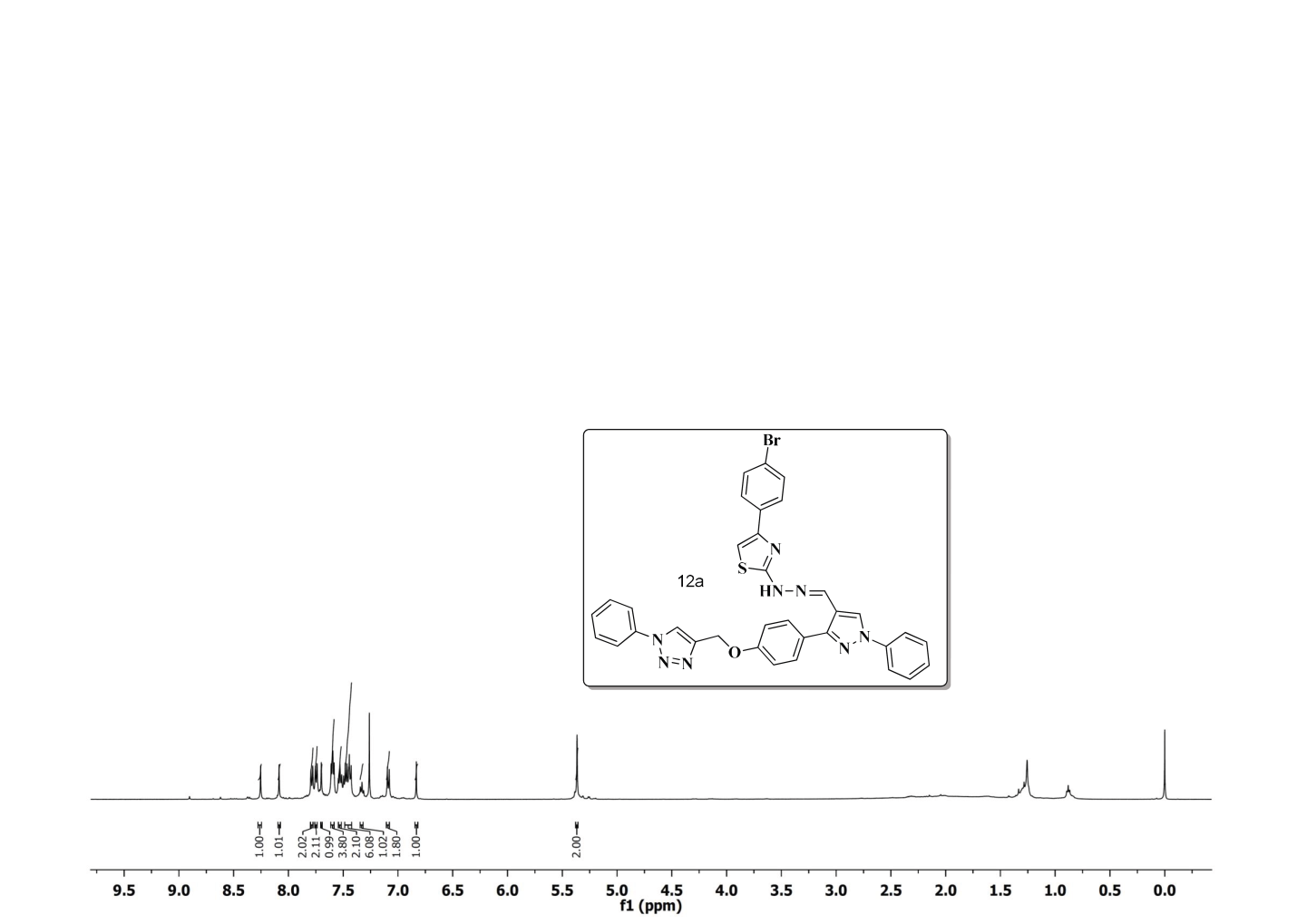


**Fig. S7. ^1^H-NMR of 4-(4-bromophenyl)-2-(2-((1-phenyl-3-(4-((1-phenyl-1H-1,2,3-triazol-4-yl)meth-oxy)-phenyl)-1H-pyrazol-4-yl)methylene) hydrazinyl) thiazole** **12a (400 MHz, CDCl_3_)**

**
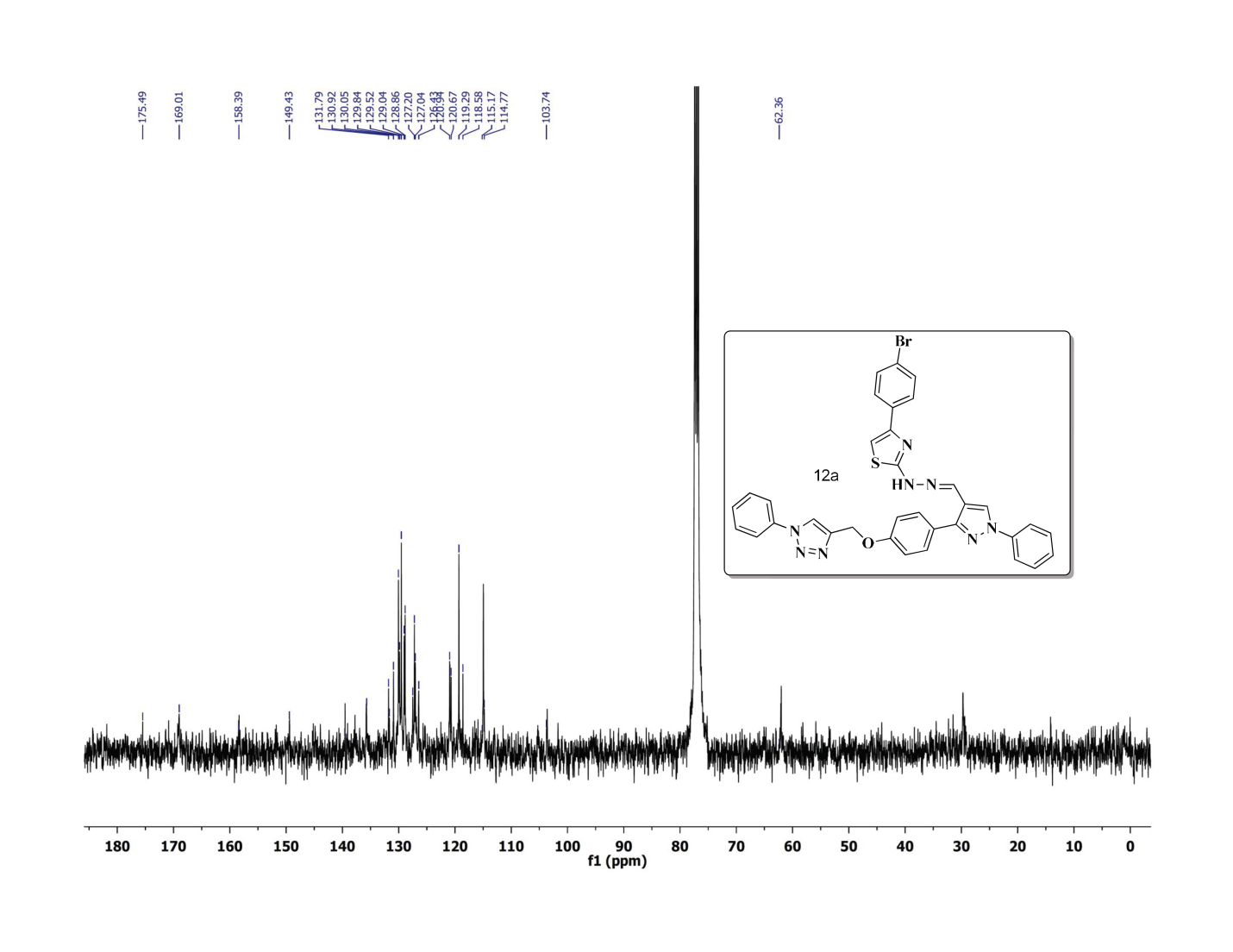
Fig. S8. ^13^C NMR of 4-(4-bromophenyl)-2-(2-((1-phenyl-3-(4-((1-phenyl-1H-1,2,3-triazol-4-yl)meth-oxy)-phenyl)-1H-pyrazol-4-yl)methylene) hydrazinyl) thiazole** **12a (100 MHz, CDCl_3_)**

**Fig. S9.**
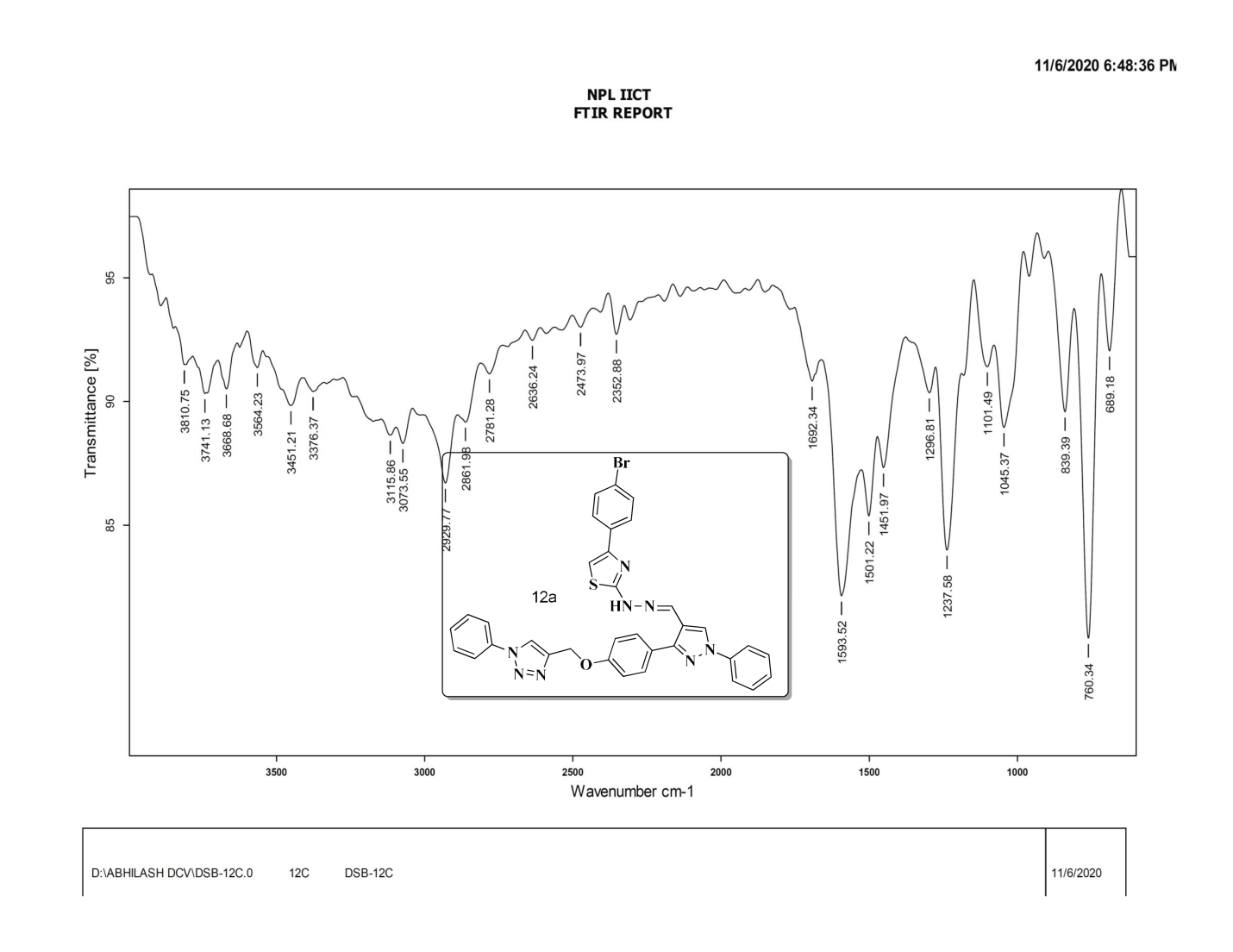
**FT-IR of 4-(4-bromophenyl)-2-(2-((1-phenyl-3-(4-((1-phenyl-1H-1,2,3-triazol-4-yl)meth-oxy)-phenyl)-1H-pyrazol-4-yl)methylene) hydrazinyl) thiazole** **12a**


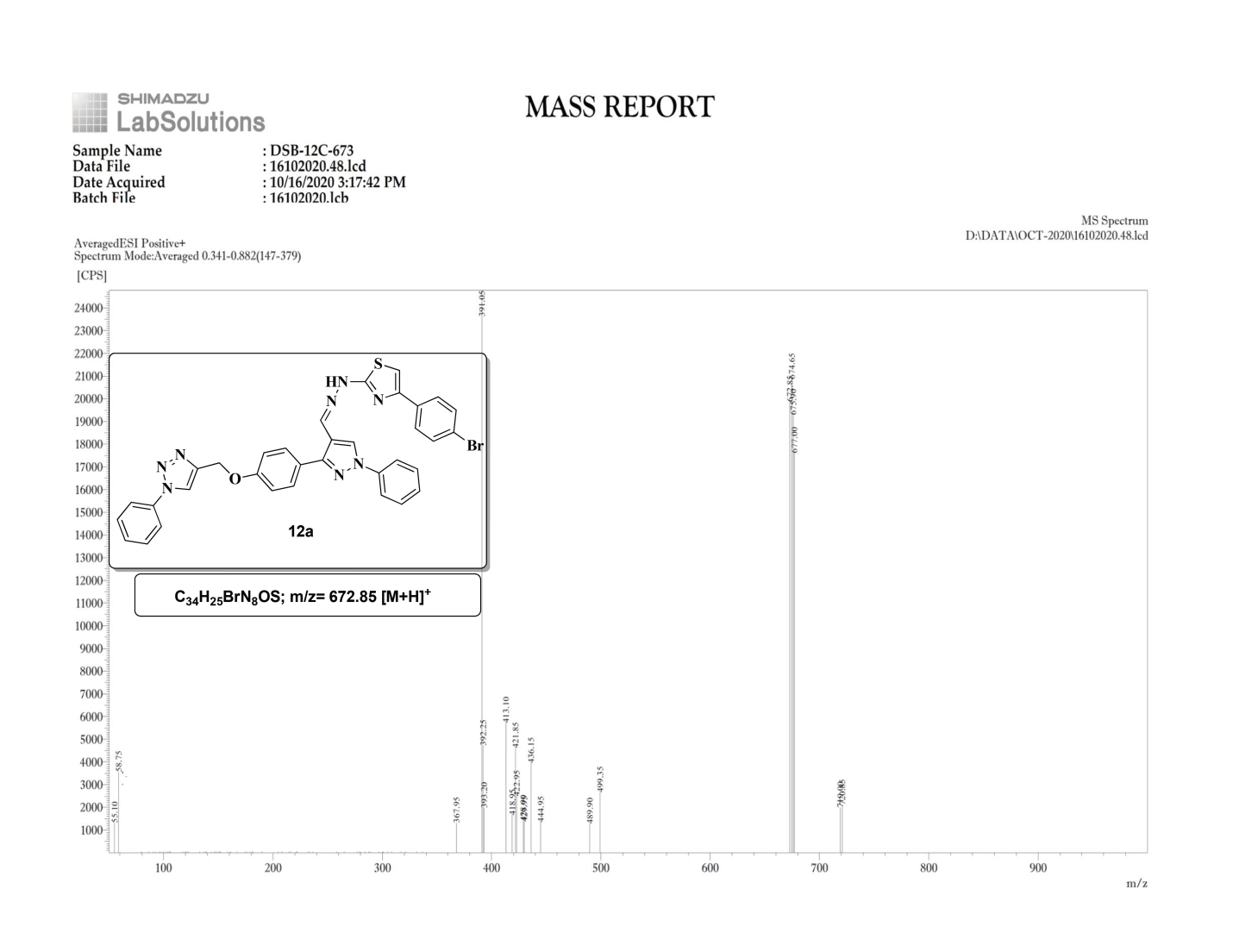


**Fig. S10. ESI-Mass of 4-(4-bromophenyl)-2-(2-((1-phenyl-3-(4-((1-phenyl-1H-1,2,3-triazol-4-yl)meth-oxy)-phenyl)-1H-pyrazol-4-yl)methylene) hydrazinyl) thiazole** **12a**


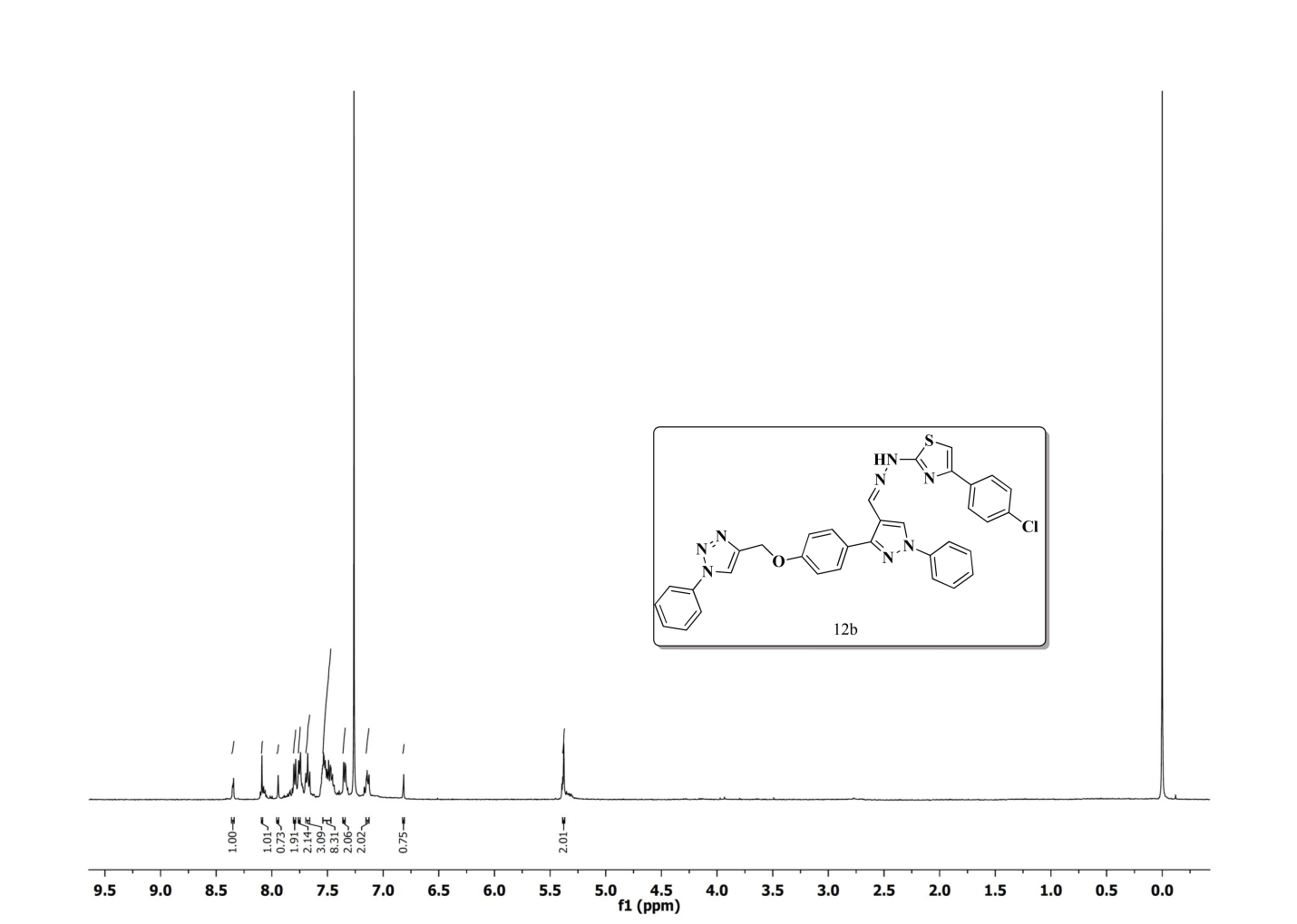


**Fig. S11. ^1^H NMR of 4-(4-chlorophenyl)-2-(2-((1-phenyl-3-(4-((1-phenyl-1H-1,2,3-triazol-4-yl)meth-oxy)-phenyl)-1H-pyrazol-4-yl)methylene)hydrazinyl)thiazole 12b (400 MHz, CDCl_3_)**

**Fig. S12.**
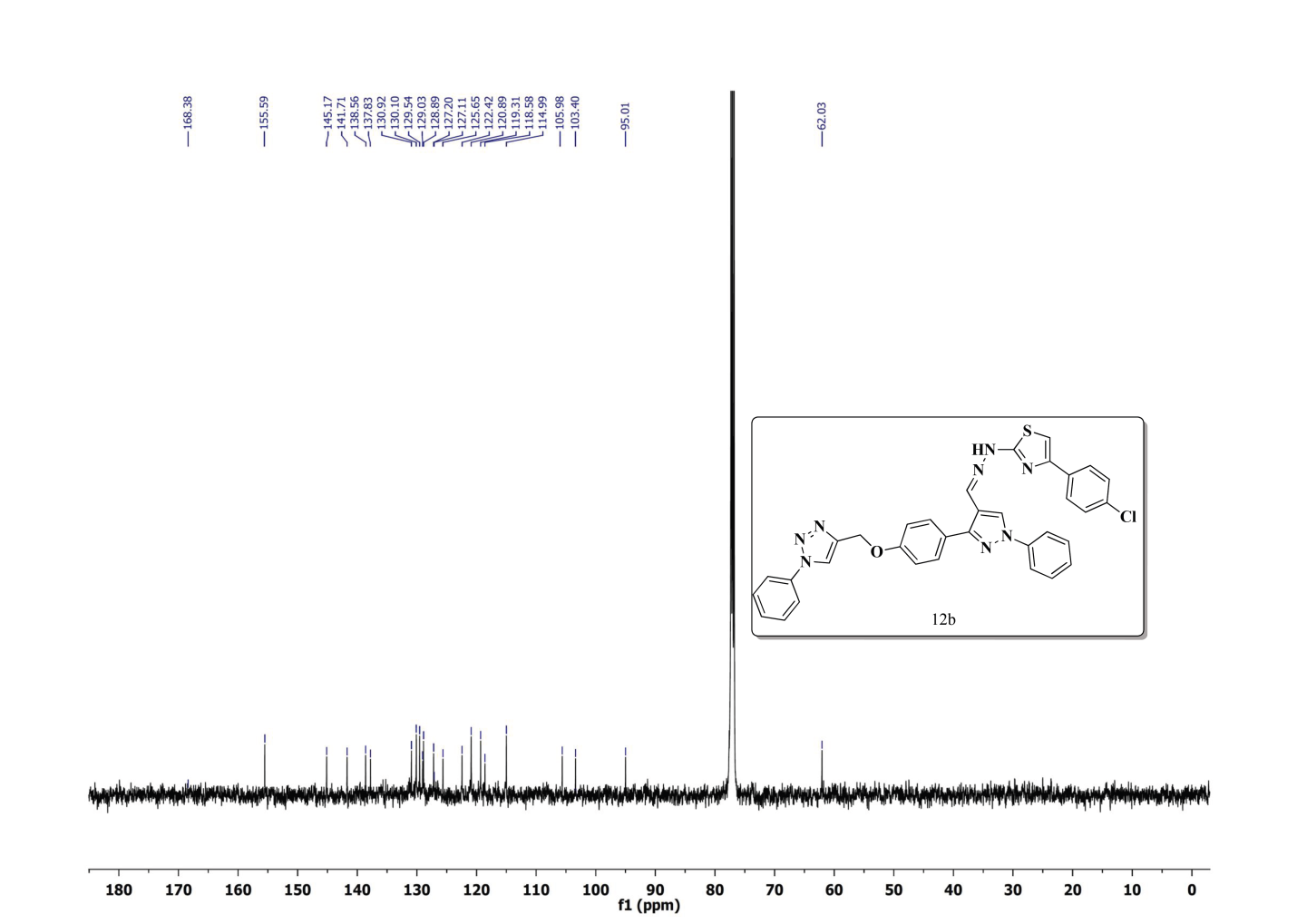
**^13^C NMR of 4-(4-chlorophenyl)-2-(2-((1-phenyl-3-(4-((1-phenyl-1H-1,2,3-triazol-4-yl)meth-oxy)-phenyl)-1H-pyrazol-4-yl)methylene)hydrazinyl)thiazole 12b (100 MHz, CDCl_3_)**

**
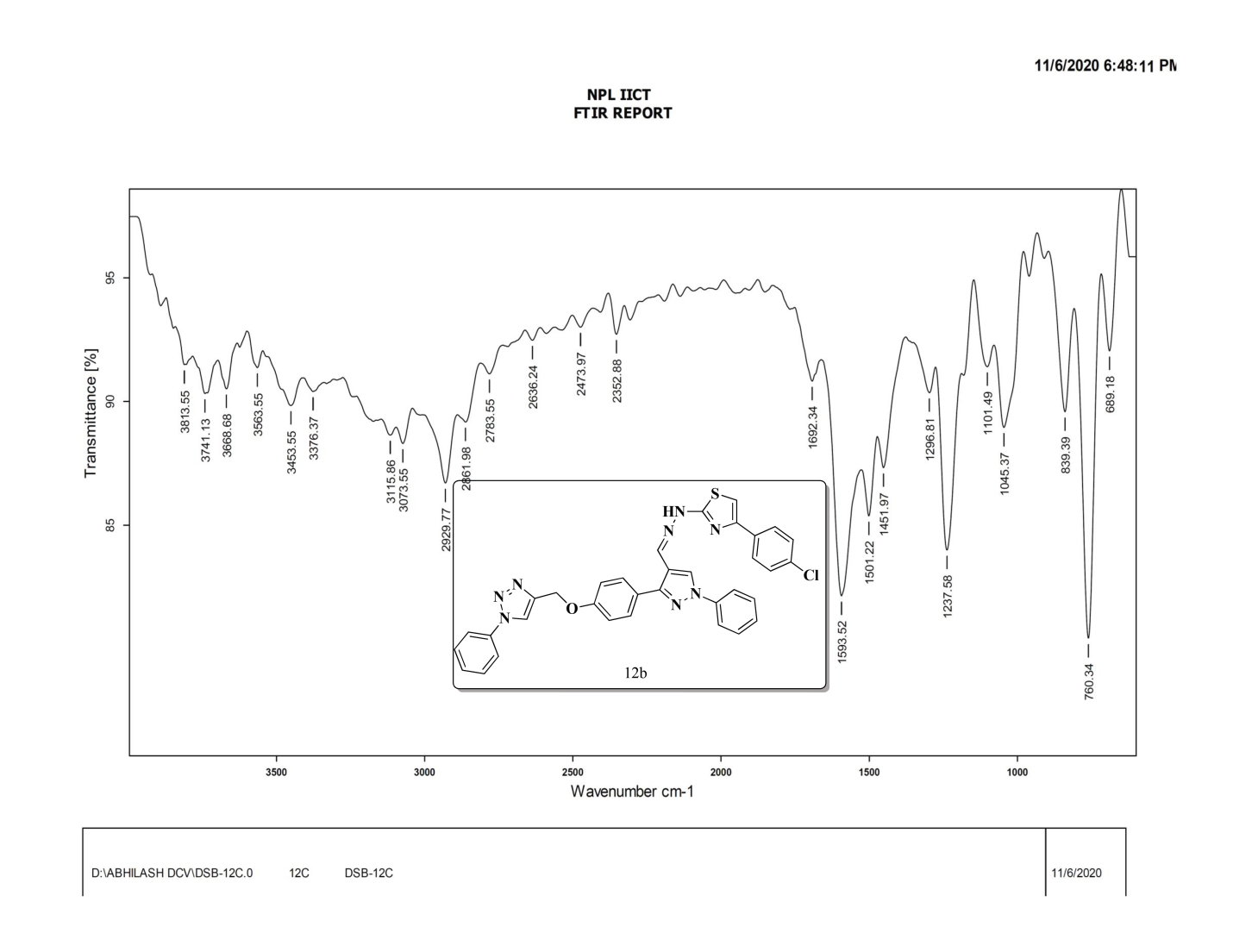
**

**Fig. S13. FT-IR of 4-(4-chlorophenyl)-2-(2-((1-phenyl-3-(4-((1-phenyl-1H-1,2,3-triazol-4-yl)meth-oxy)-phenyl)-1H-pyrazol-4-yl)methylene)hydrazinyl)thiazole 12b**


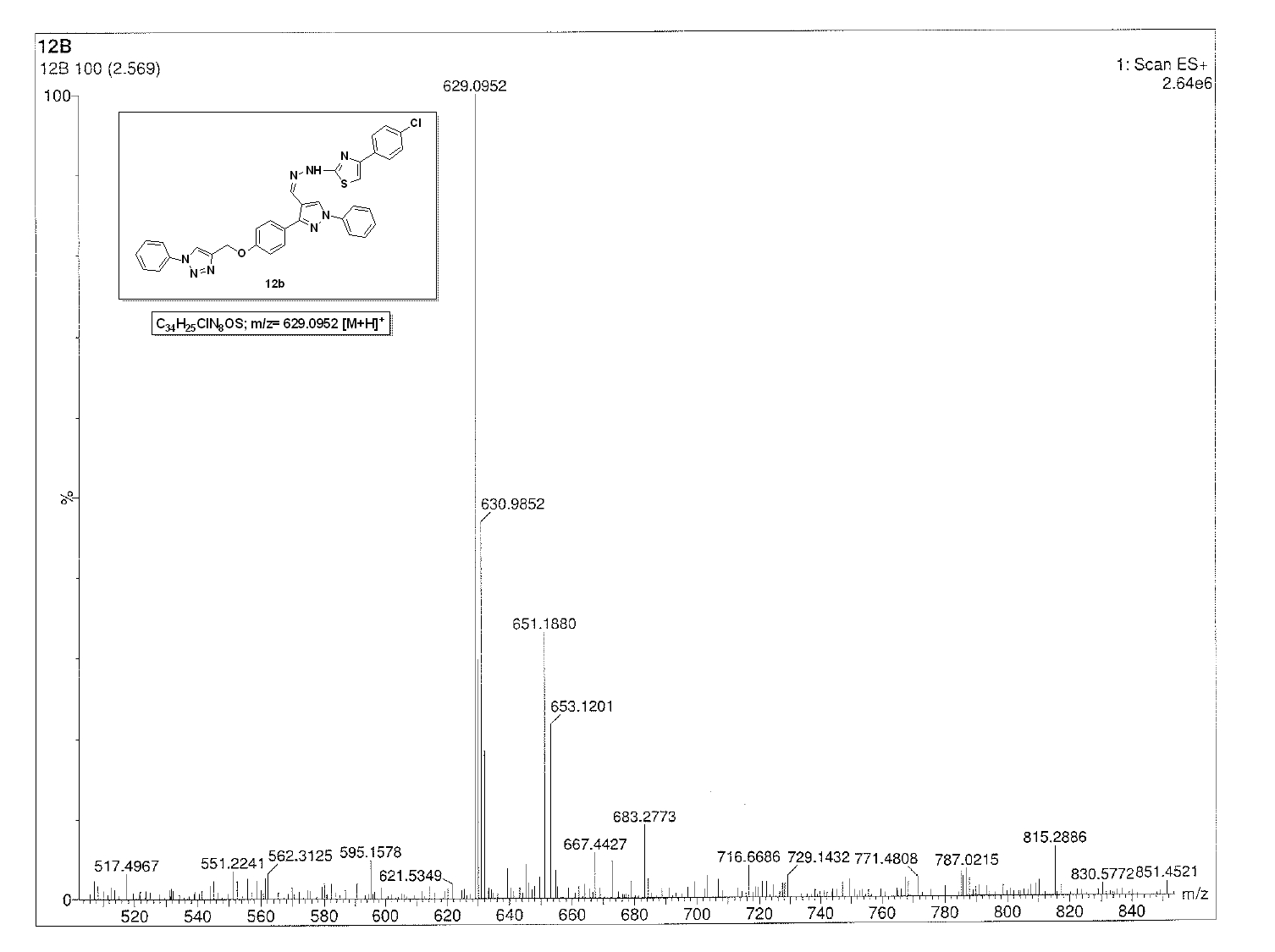


**Fig. S14. ESI-Mass of 4-(4-chlorophenyl)-2-(2-((1-phenyl-3-(4-((1-phenyl-1H-1,2,3-triazol-4-yl)meth-oxy)-phenyl)-1H-pyrazol-4-yl)methylene)hydrazinyl)thiazole 12b**

**
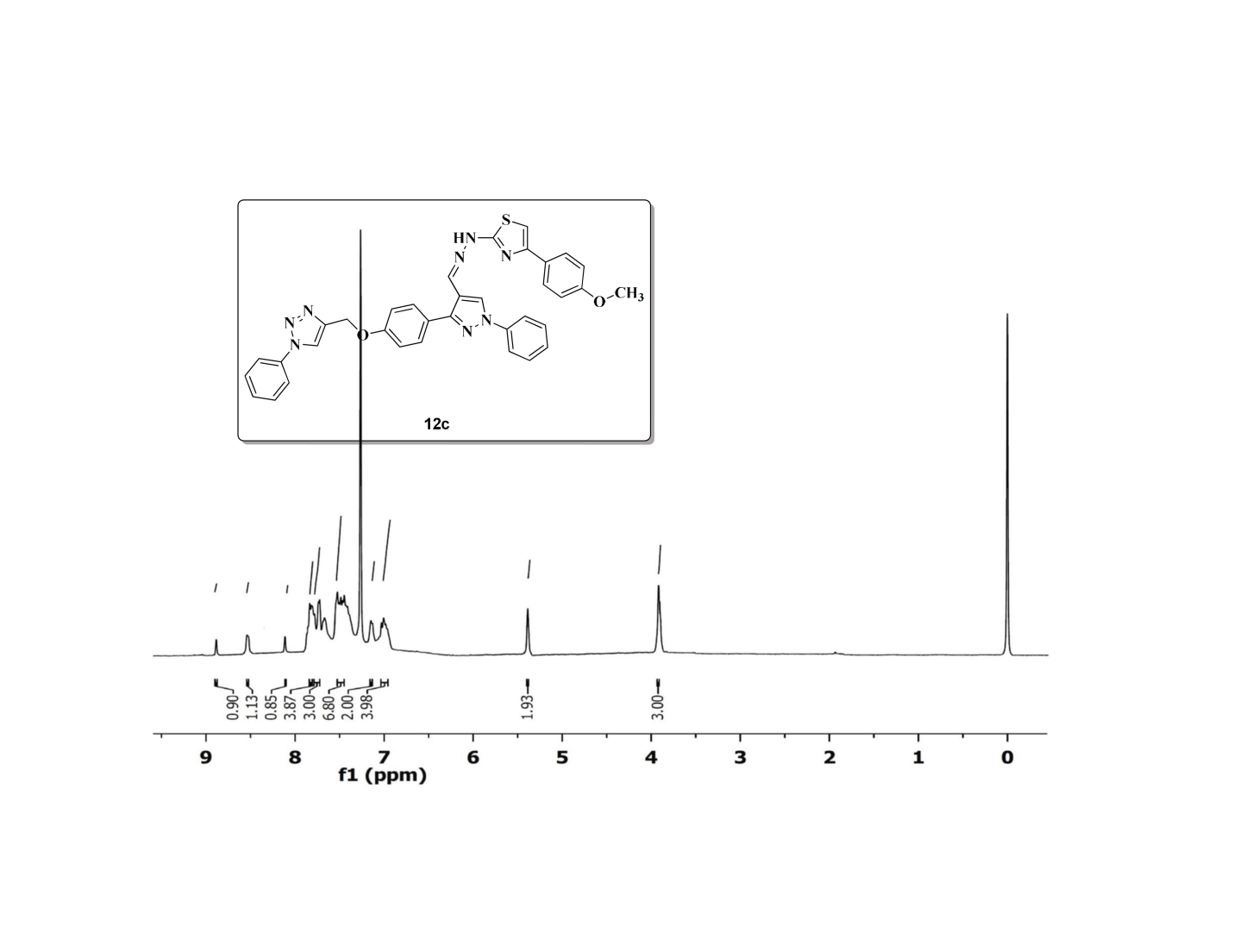
Fig. S15. ^1^H NMR of 4-(4-methoxyphenyl)-2-(2-((1-phenyl-3-(4-((1-phenyl-1H-1,2,3-triazol-4-yl)meth-oxy)-phenyl)-1H-pyrazol-4-yl)methylene)hydrazinyl)thiazole 12c (400 MHz, CDCl_3_)**

**Fig. S16.**
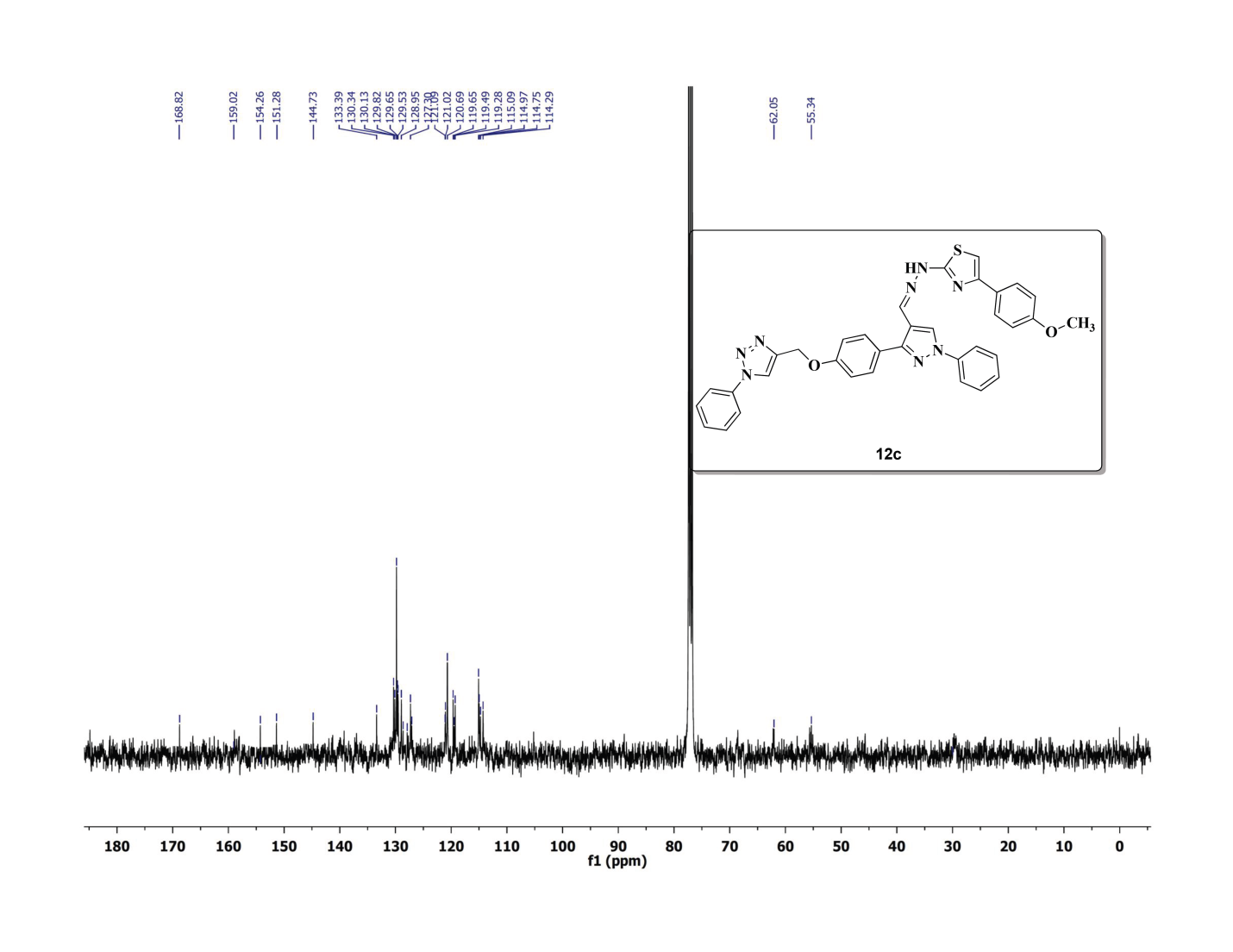
**^13^C NMR of 4-(4-methoxyphenyl)-2-(2-((1-phenyl-3-(4-((1-phenyl-1H-1,2,3-triazol-4-yl)meth-oxy)-phenyl)-1H-pyrazol-4-yl)methylene)hydrazinyl)thiazole 12c (100 MHz, CDCl_3_)**


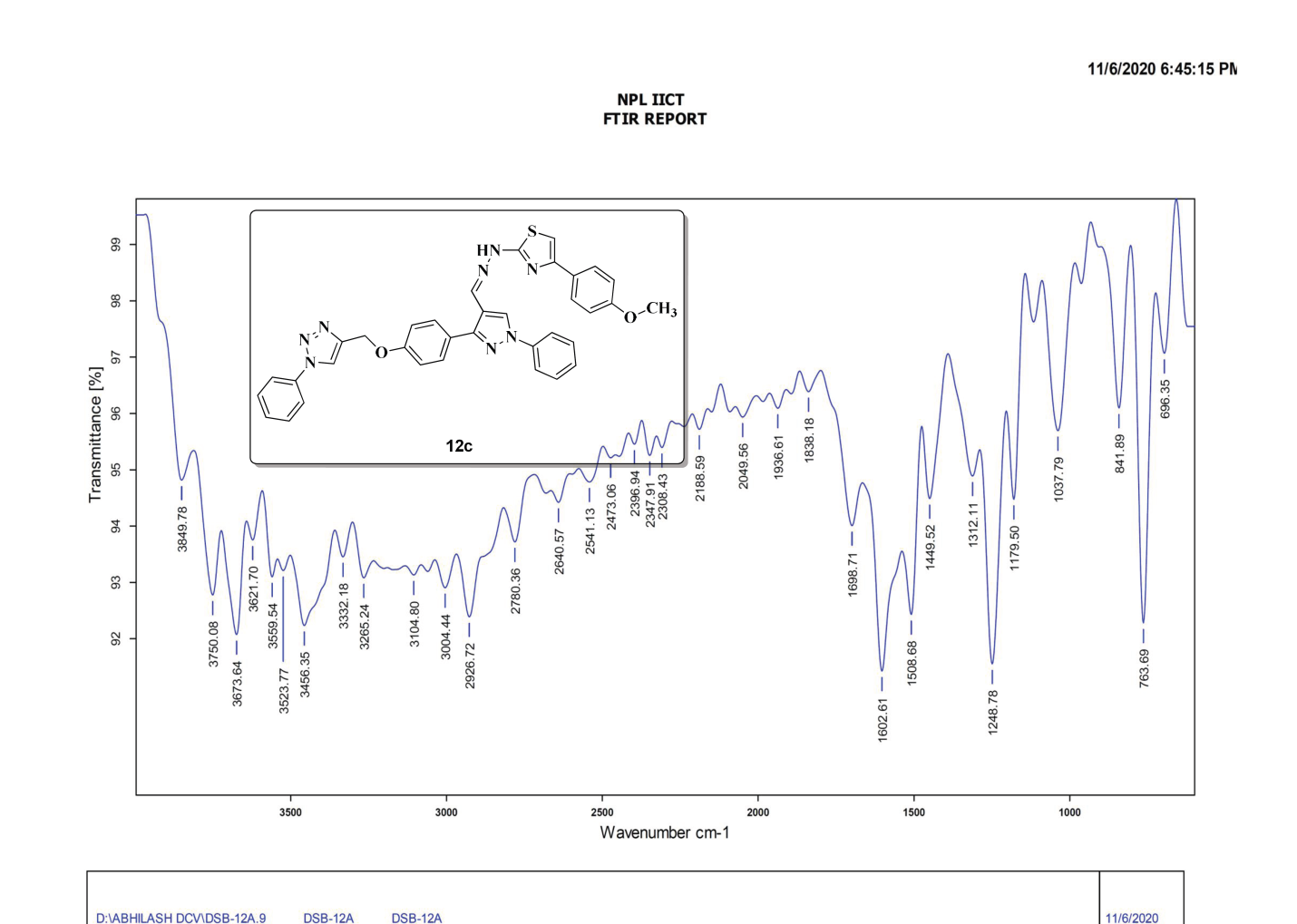


**Fig. S17. FT-IR of 4-(4-methoxyphenyl)-2-(2-((1-phenyl-3-(4-((1-phenyl-1H-1,2,3-triazol-4-yl)meth-oxy)-phenyl)-1H-pyrazol-4-yl)methylene)hydrazinyl)thiazole 12c**

**
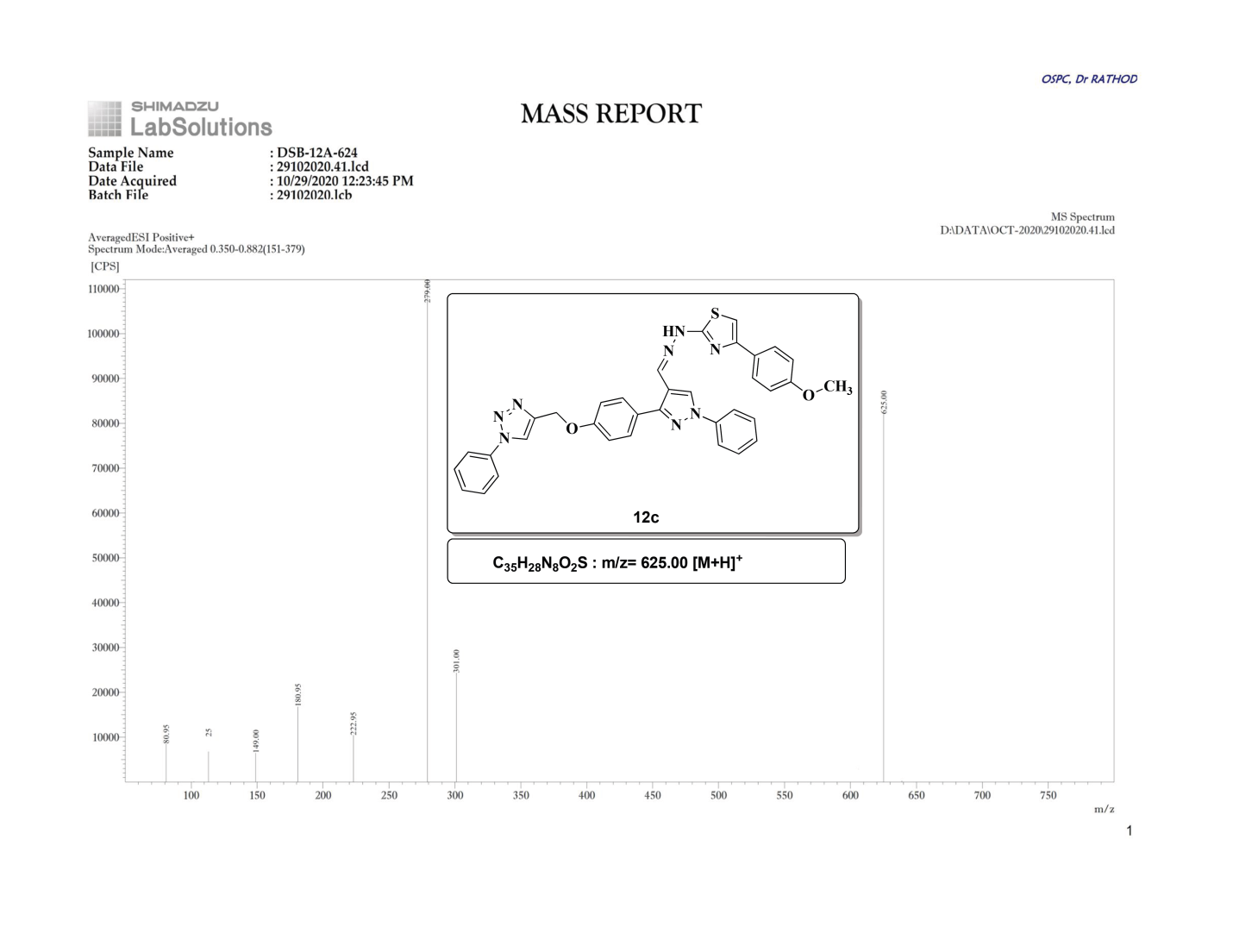
Fig. S18. ESI-Mass 4-(4-methoxyphenyl)-2-(2-((1-phenyl-3-(4-((1-phenyl-1H-1,2,3-triazol-4-yl)meth-oxy)-phenyl)-1H-pyrazol-4-yl)methylene)hydrazinyl)thiazole 12c**

**
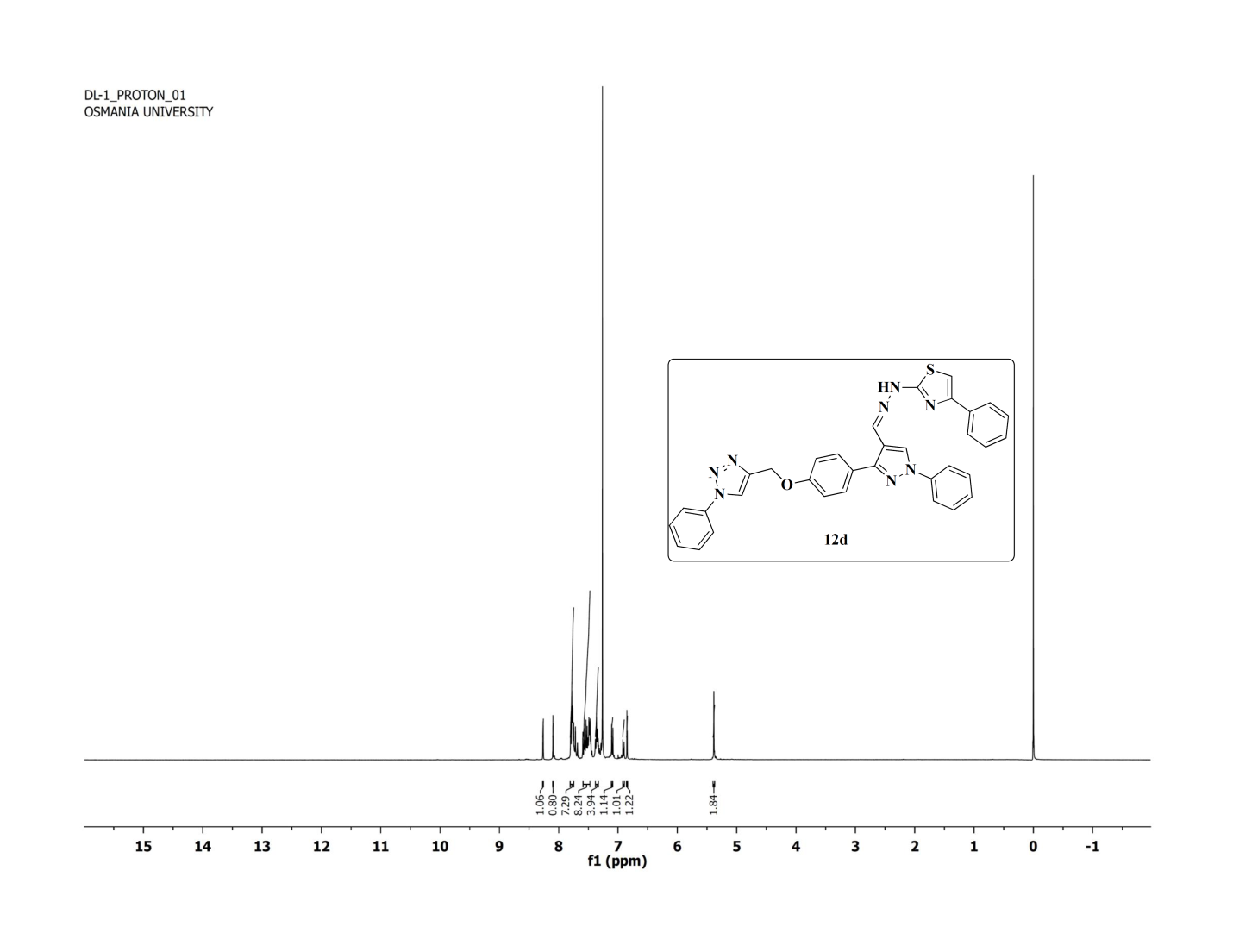
Fig. S19. ^1^H NMR of 4-phenyl-2-(2-((1-phenyl-3-(4-((1-phenyl-1H-1,2,3-triazol-4-yl)methoxy)phenyl)-1H-pyrazol-4-yl)methylene)hydrazinyl)thiazole 12d (400 MHz, CDCl_3_)**

**Fig. S20.**
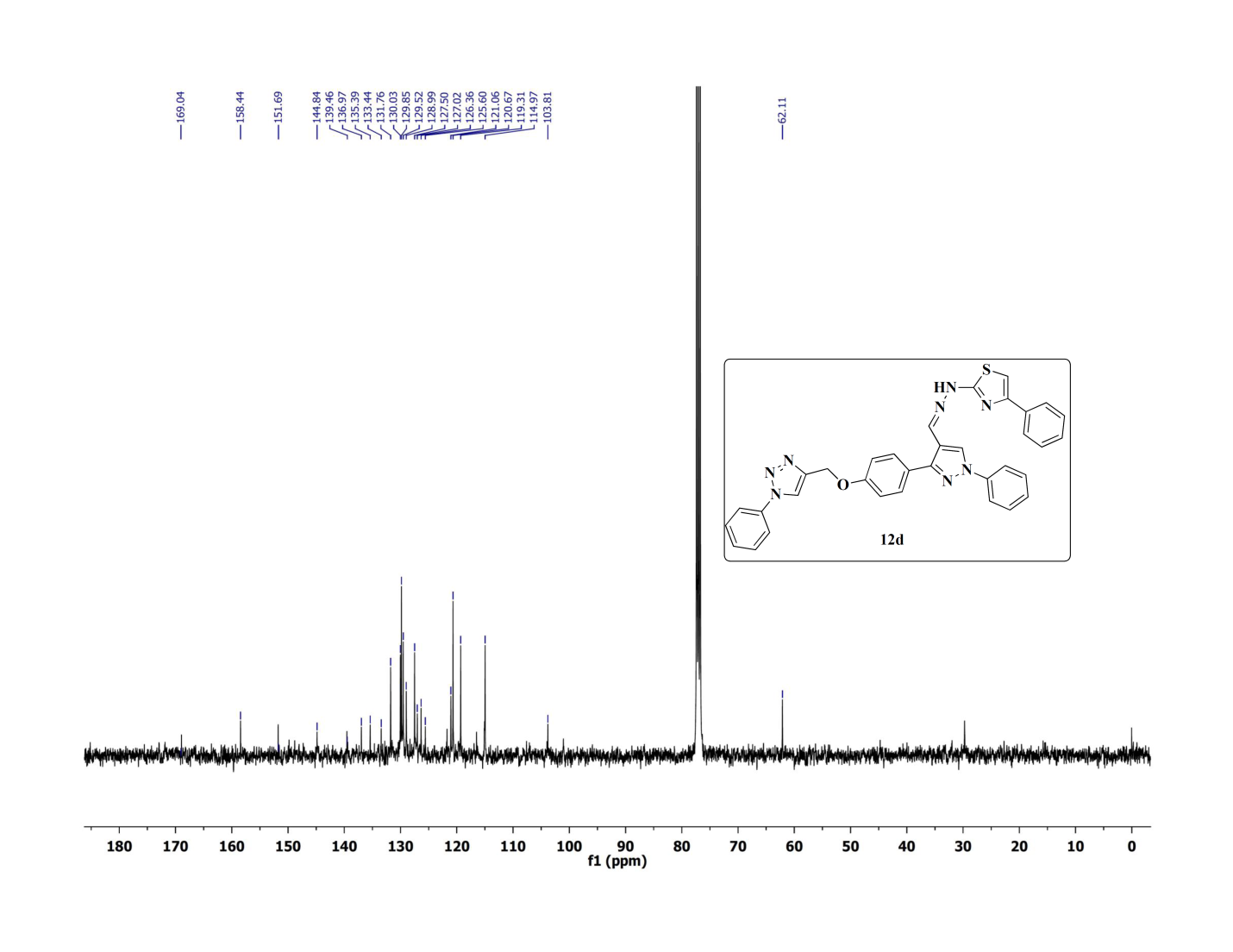
**^13^C NMR of 4-phenyl-2-(2-((1-phenyl-3-(4-((1-phenyl-1H-1,2,3-triazol-4-yl)methoxy)phenyl)-1H-pyrazol-4-yl)methylene)hydrazinyl)thiazole 12d (100 MHz, CDCl_3_)**


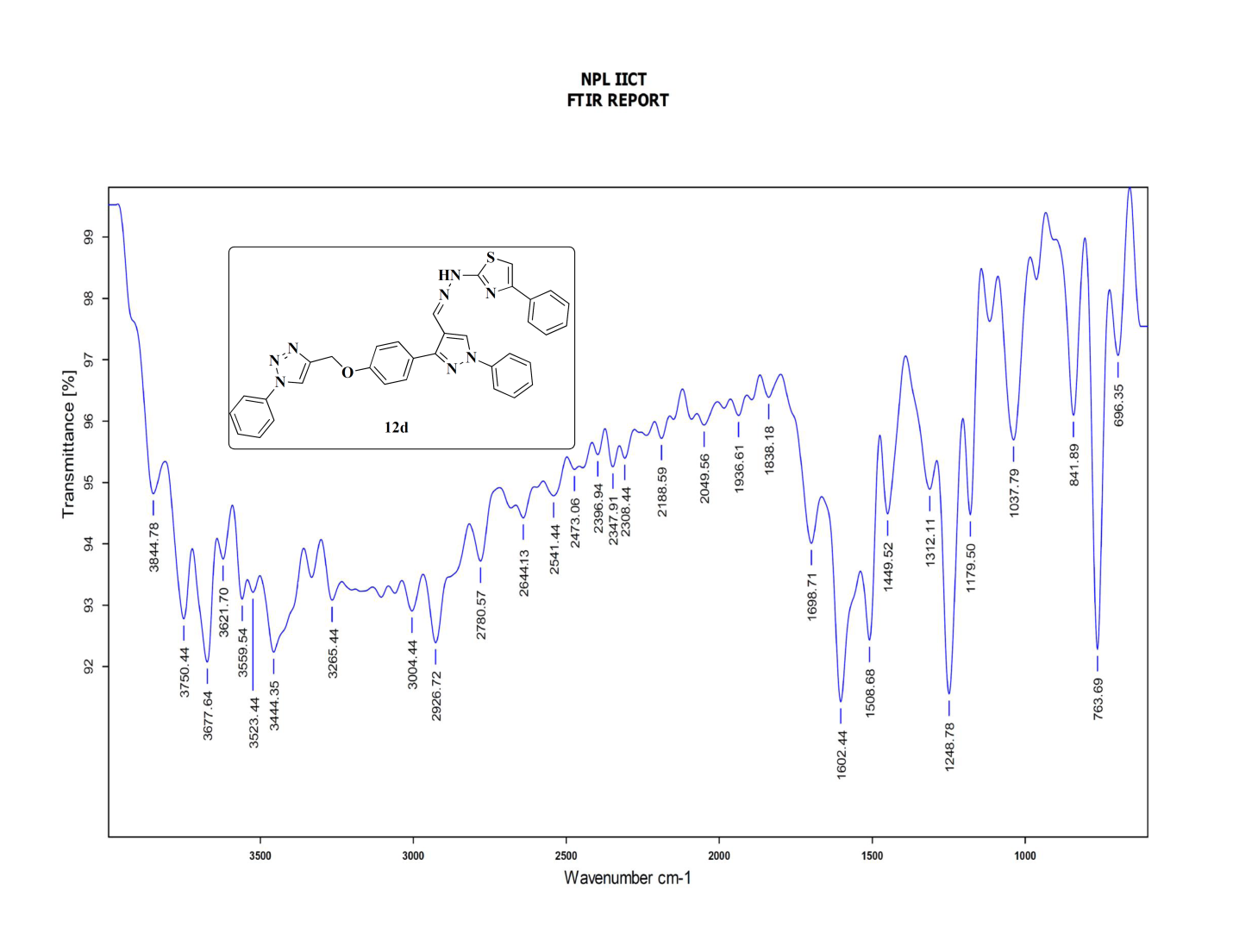


**Fig. S21. FT-IR of 4-phenyl-2-(2-((1-phenyl-3-(4-((1-phenyl-1H-1,2,3-triazol-4-yl)methoxy)phenyl)-1H-pyrazol-4-yl)methylene)hydrazinyl)thiazole 10d**

**Fig. S22.**
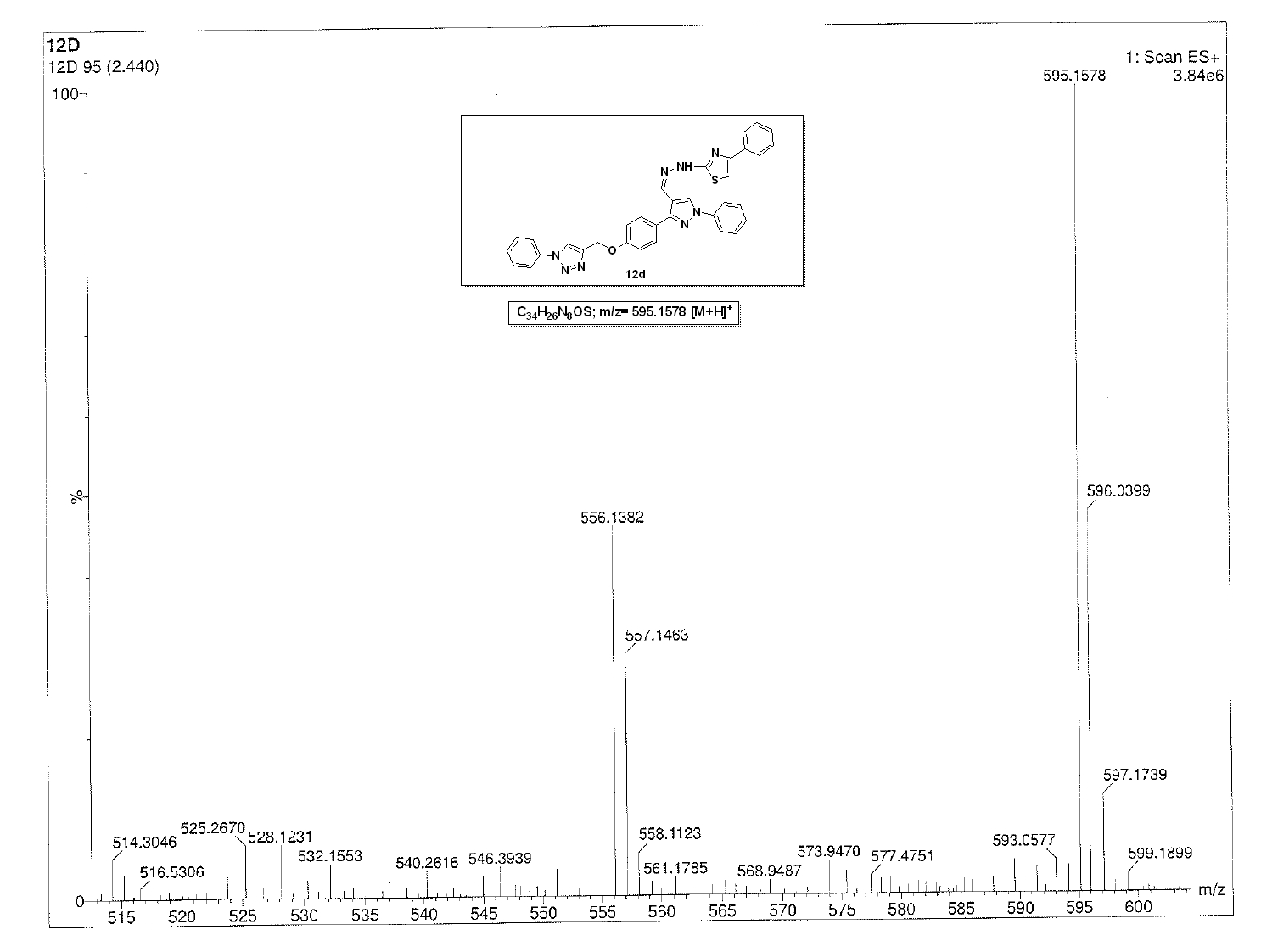
**ESI-Mass of 4-phenyl-2-(2-((1-phenyl-3-(4-((1-phenyl-1H-1,2,3-triazol-4-yl)methoxy)phenyl)-1H-pyrazol-4-yl)methylene)hydrazinyl)thiazole 12d**

**
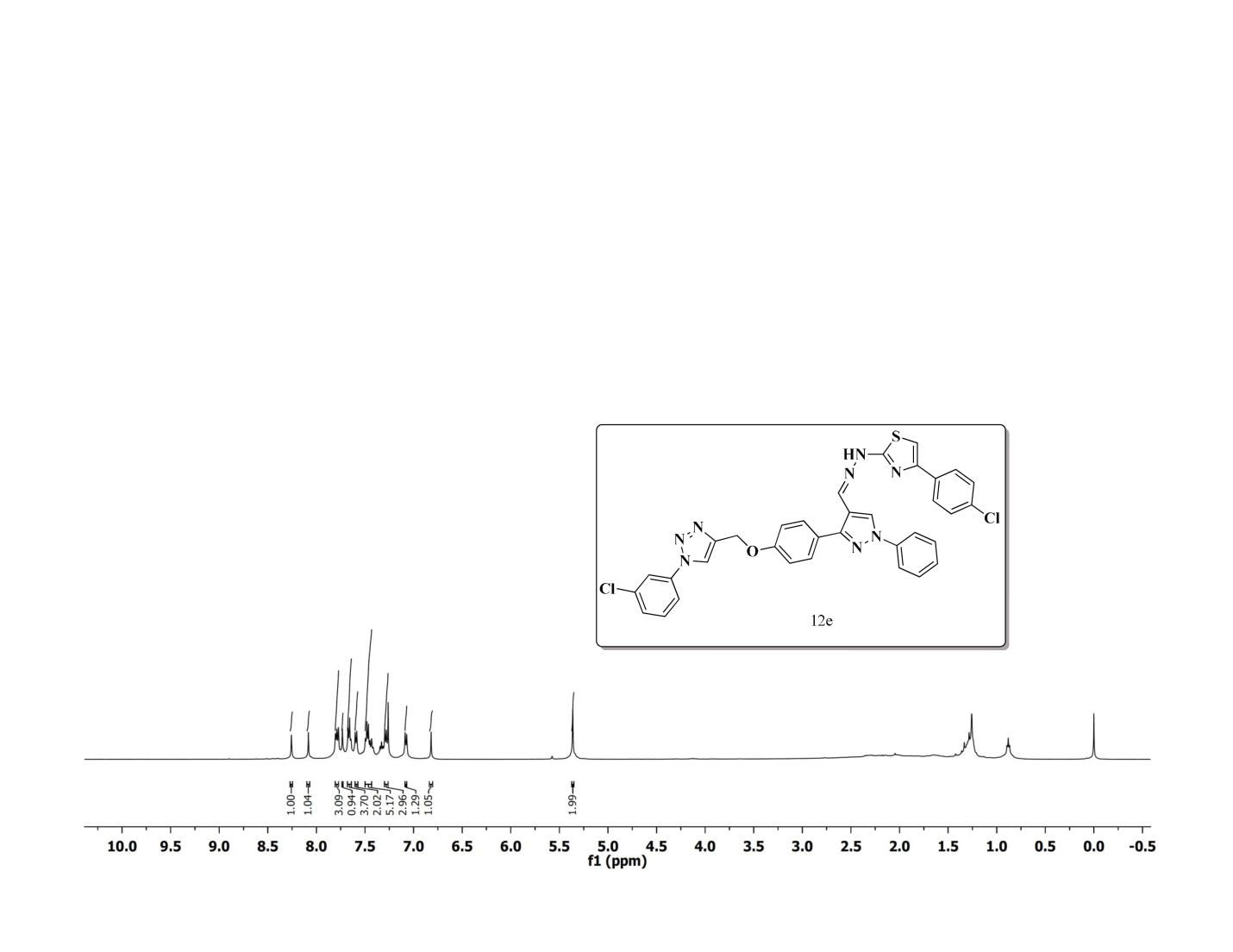
Fig. S23. ^1^H NMR of 4-(4-chlorophenyl)-2-(2-((3-(4-((1-(3-chlorophenyl)-1H-1,2,3-triazol-4-yl)methoxy)phenyl)-1-phenyl-1H-pyrazol-4-yl)methylene)hydrazinyl)thiazole 12e (400 MHz, CDCl_3_)**


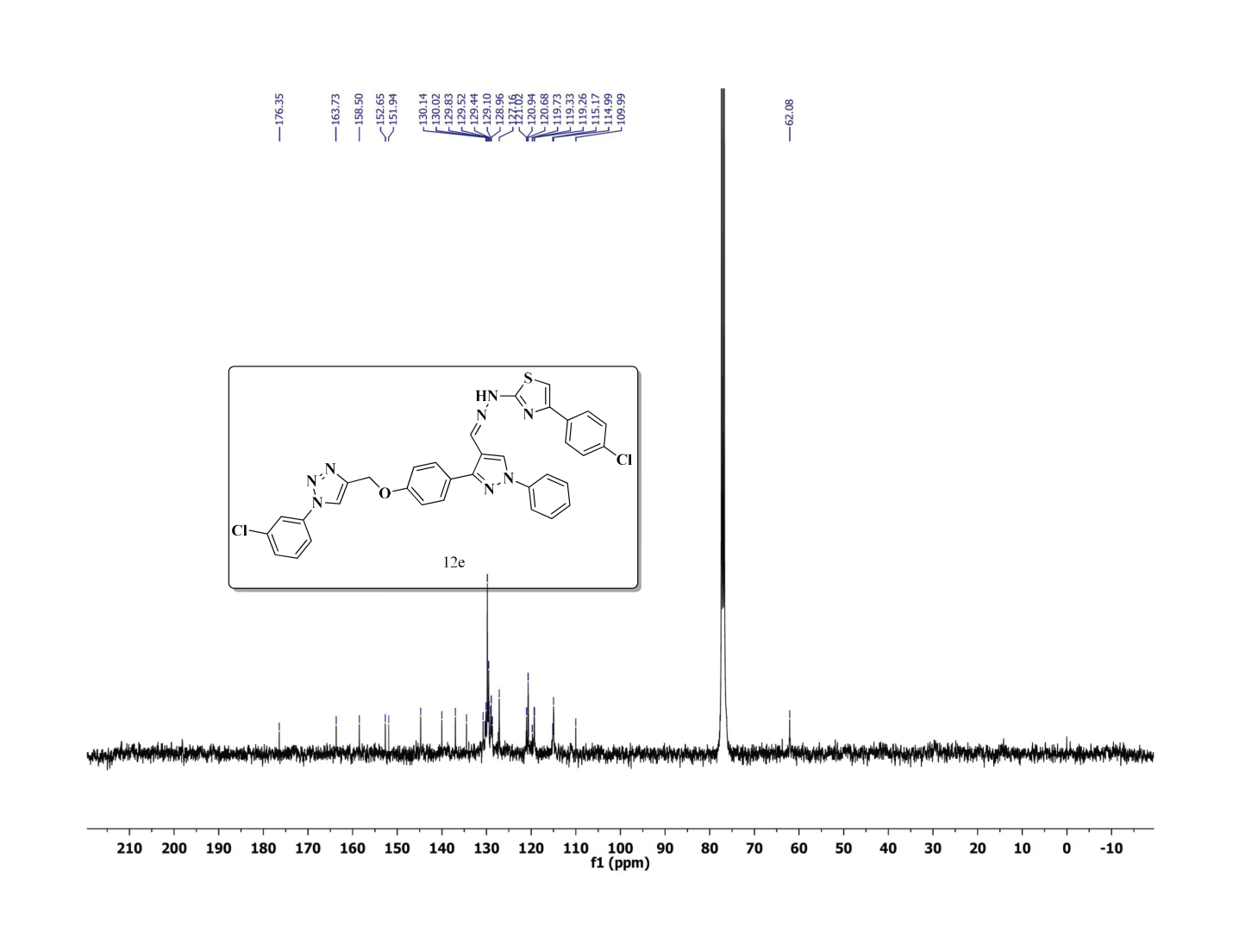


**Fig. S24. ^13^C NMR of 4-(4-chlorophenyl)-2-(2-((3-(4-((1-(3-chlorophenyl)-1H-1,2,3-triazol-4-yl)methoxy)phenyl)-1-phenyl-1H-pyrazol-4-yl)methylene)hydrazinyl)thiazole 12e (100 MHz, CDCl_3_)**


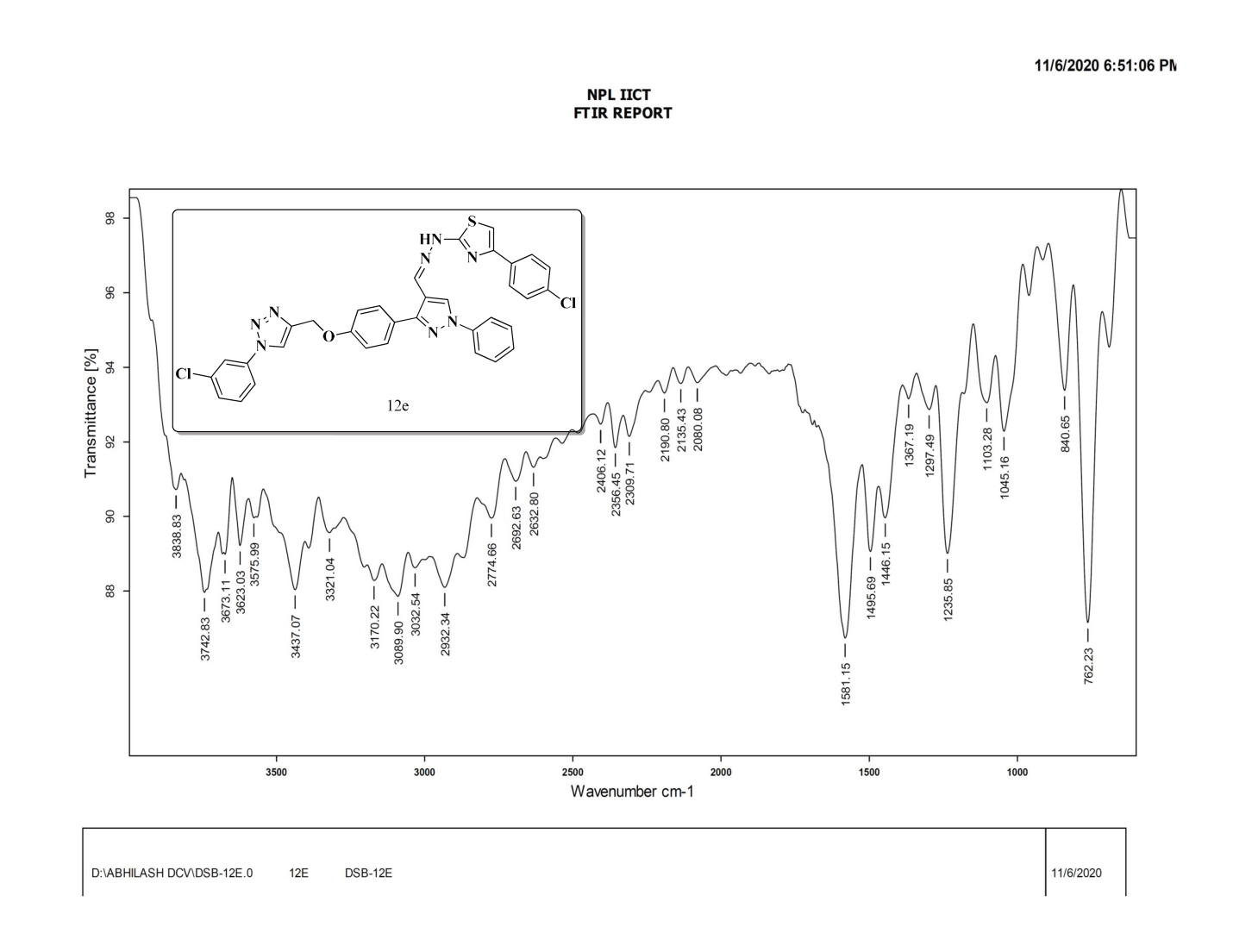


**Fig. S25. FT-IR of 4-(4-chlorophenyl)-2-(2-((3-(4-((1-(3-chlorophenyl)-1H-1,2,3-triazol-4-yl)methoxy)phenyl)-1-phenyl-1H-pyrazol-4-yl)methylene)hydrazinyl)thiazole 12e**

**
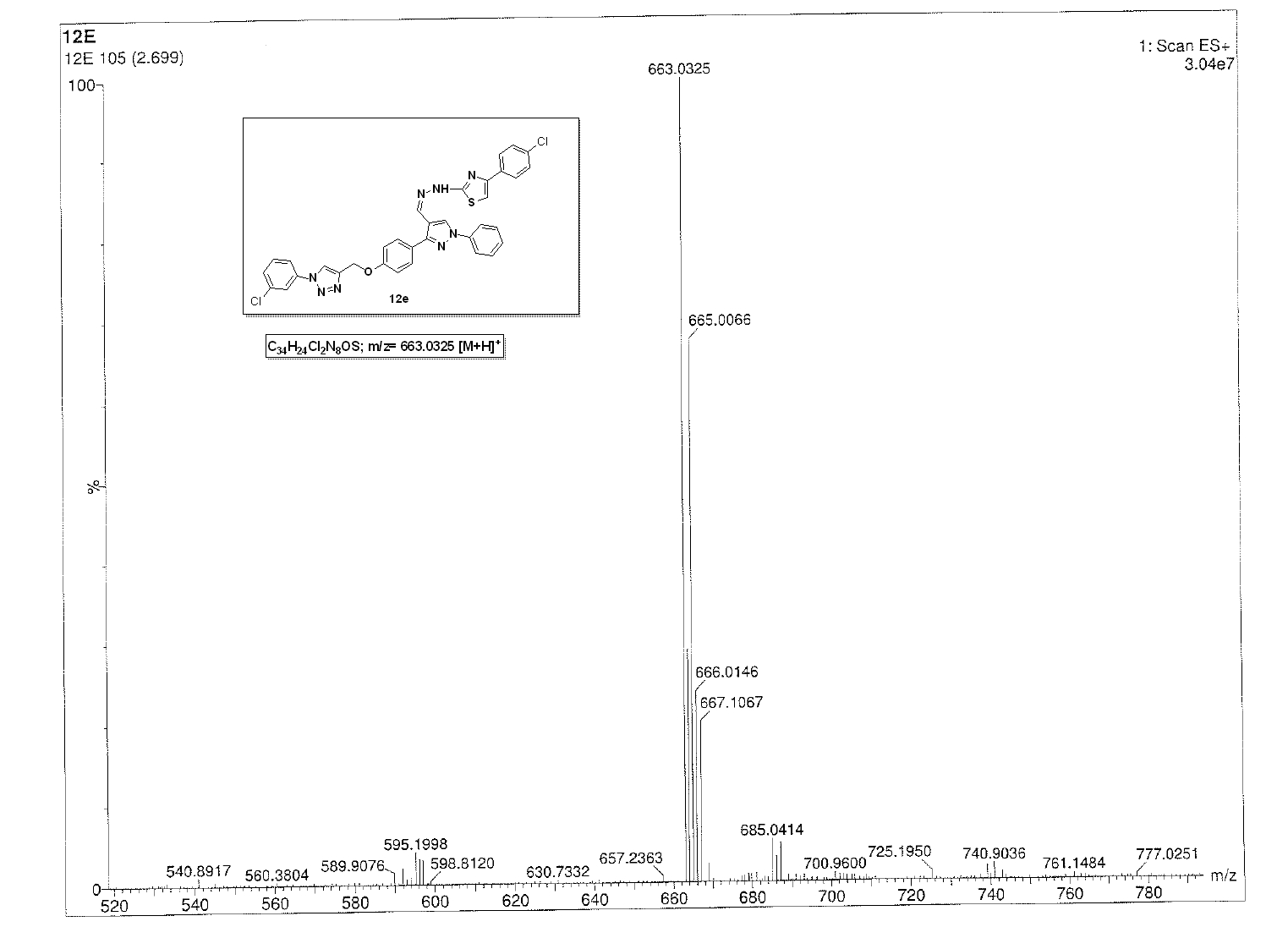
Fig. S26. ESI-Mass of 4-(4-chlorophenyl)-2-(2-((3-(4-((1-(3-chlorophenyl)-1H-1,2,3-triazol-4-yl)methoxy)phenyl)-1-phenyl-1H-pyrazol-4-yl)methylene)hydrazinyl)thiazole 12e.**


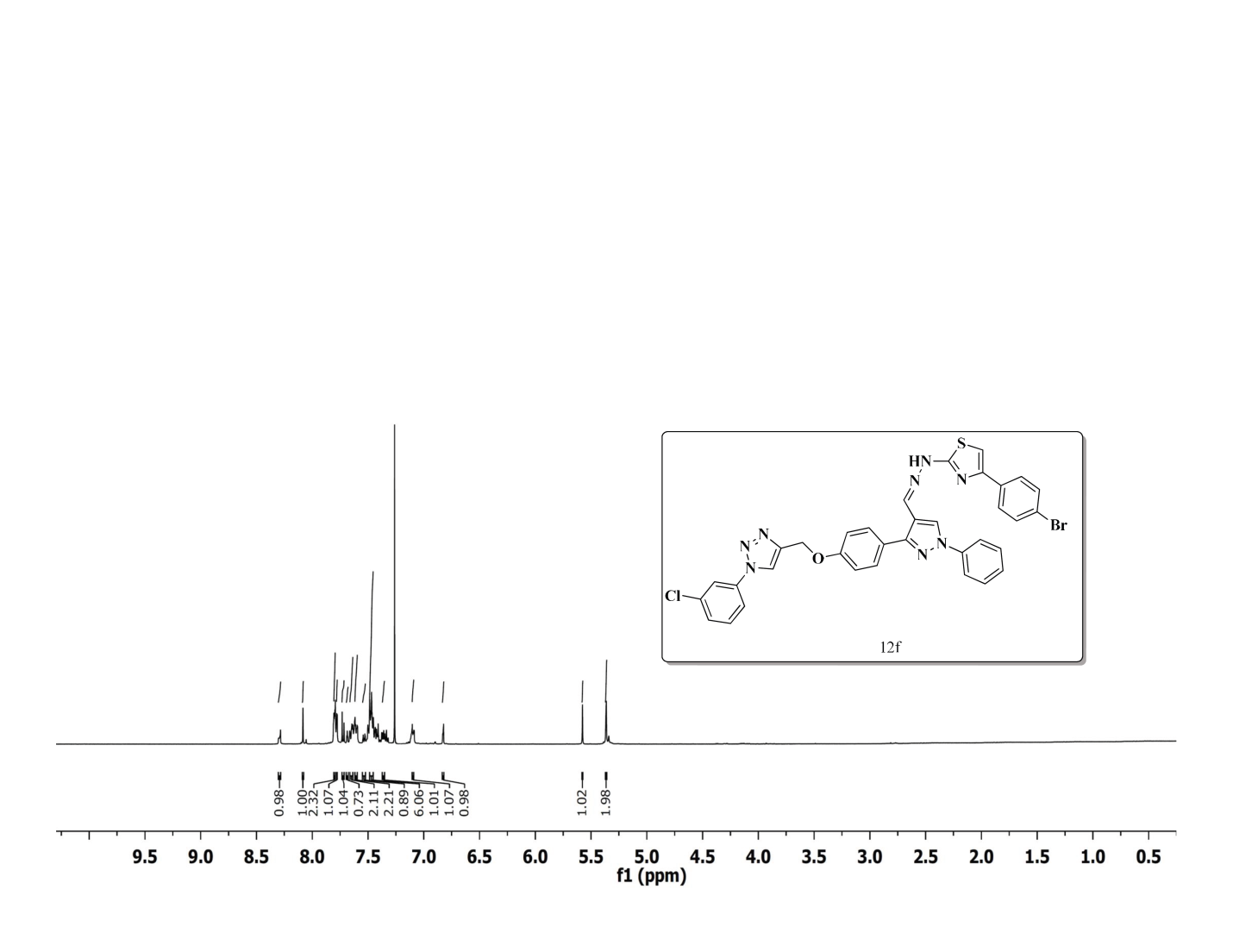


**Fig. S27. ^1^H NMR of 4-(4-bromophenyl)-2-(2-((3-(4-((1-(3-chlorophenyl)-1H-1,2,3-triazol-4-yl)methoxy)-phenyl)-1-phenyl-1H-pyrazol-4-yl)methylene)hydrazinyl)thiazole 12f (400 MHz, CDCl_3_)**

**
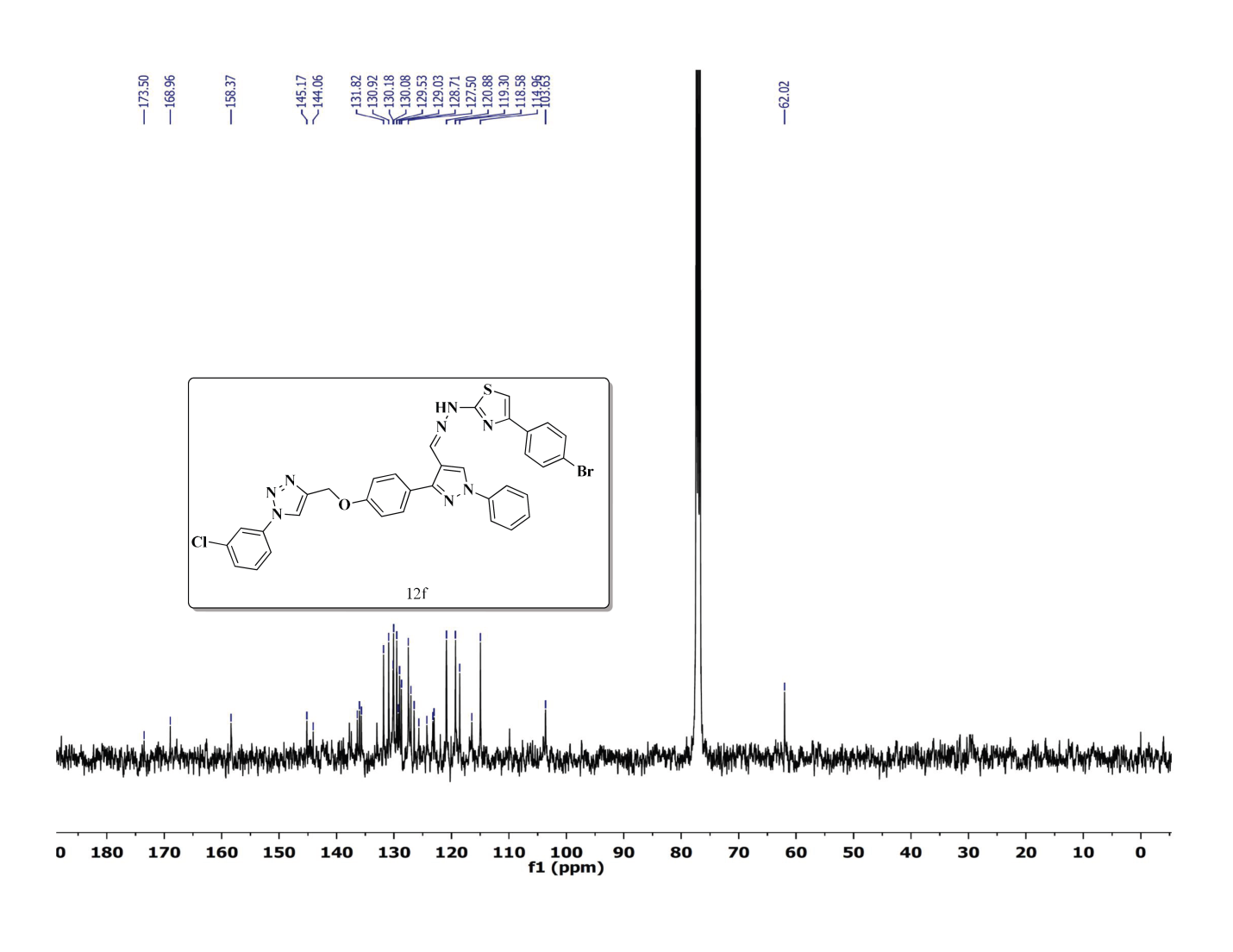
Fig. S28. ^13^C NMR of 4-(4-bromophenyl)-2-(2-((3-(4-((1-(3-chlorophenyl)-1H-1,2,3-triazol-4-yl)methoxy)-phenyl)-1-phenyl-1H-pyrazol-4-yl)methylene)hydrazinyl)thiazole 12f (100 MHz, CDCl_3_)**


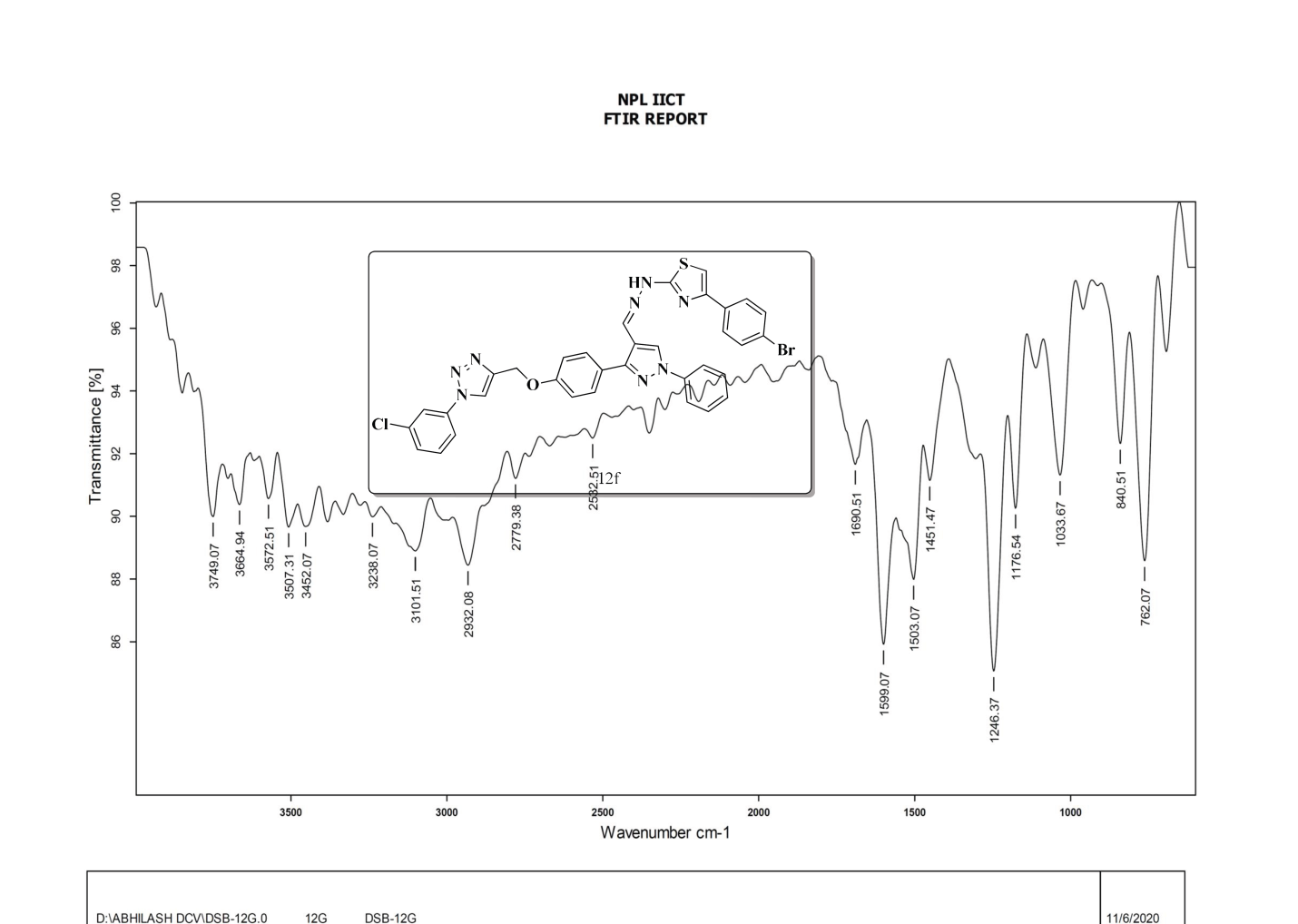


**Fig. S29. FT-IR of 4-(4-bromophenyl)-2-(2-((3-(4-((1-(3-chlorophenyl)-1H-1,2,3-triazol-4-yl)methoxy)-phenyl)-1-phenyl-1H-pyrazol-4-yl)methylene)hydrazinyl)thiazole 12f**

**
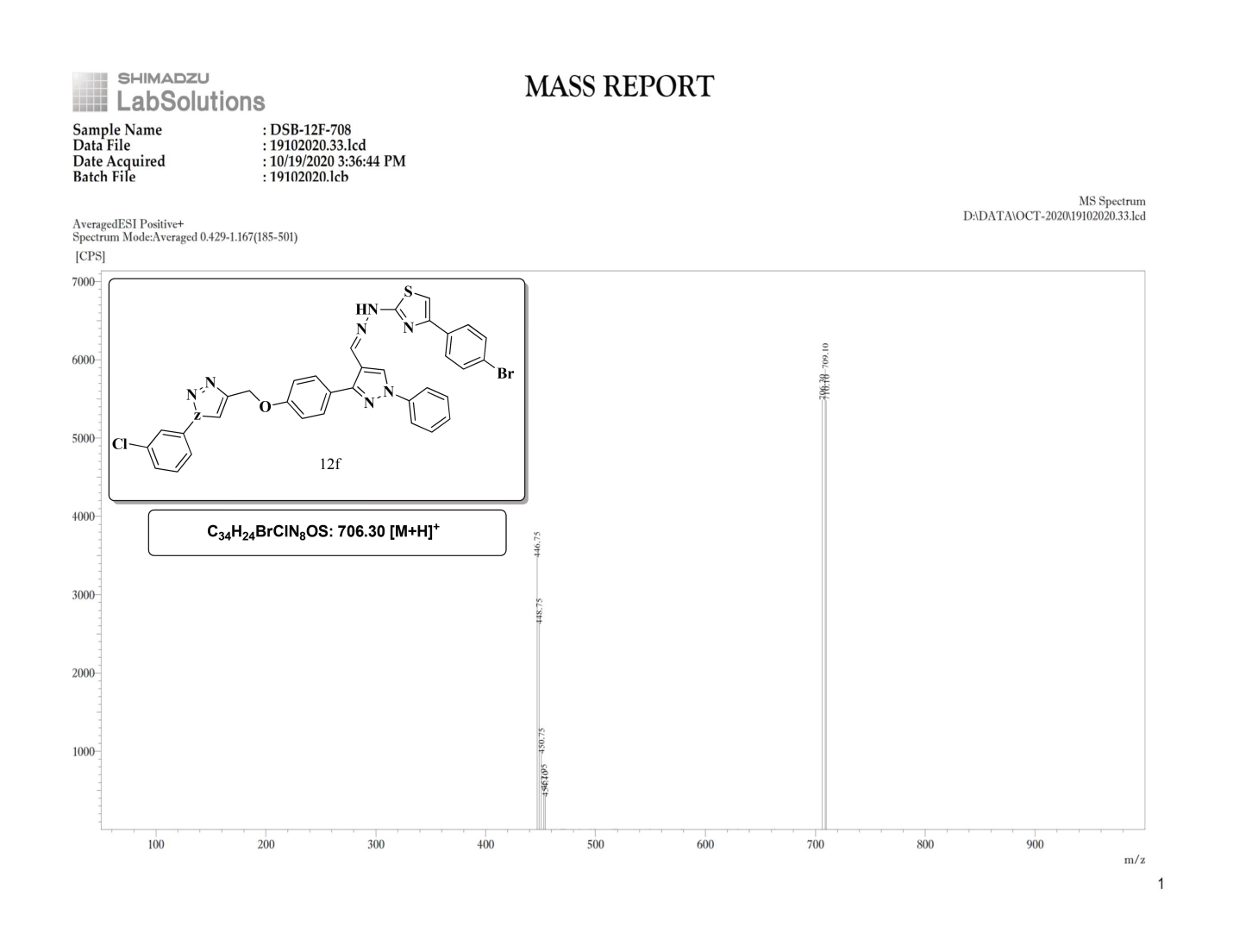
Fig. S30. ESI-Mass of 4-(4-bromophenyl)-2-(2-((3-(4-((1-(3-chlorophenyl)-1H-1,2,3-triazol-4-yl)methoxy)-phenyl)-1-phenyl-1H-pyrazol-4-yl)methylene)hydrazinyl)thiazole 10f**

**Fig. S31.**
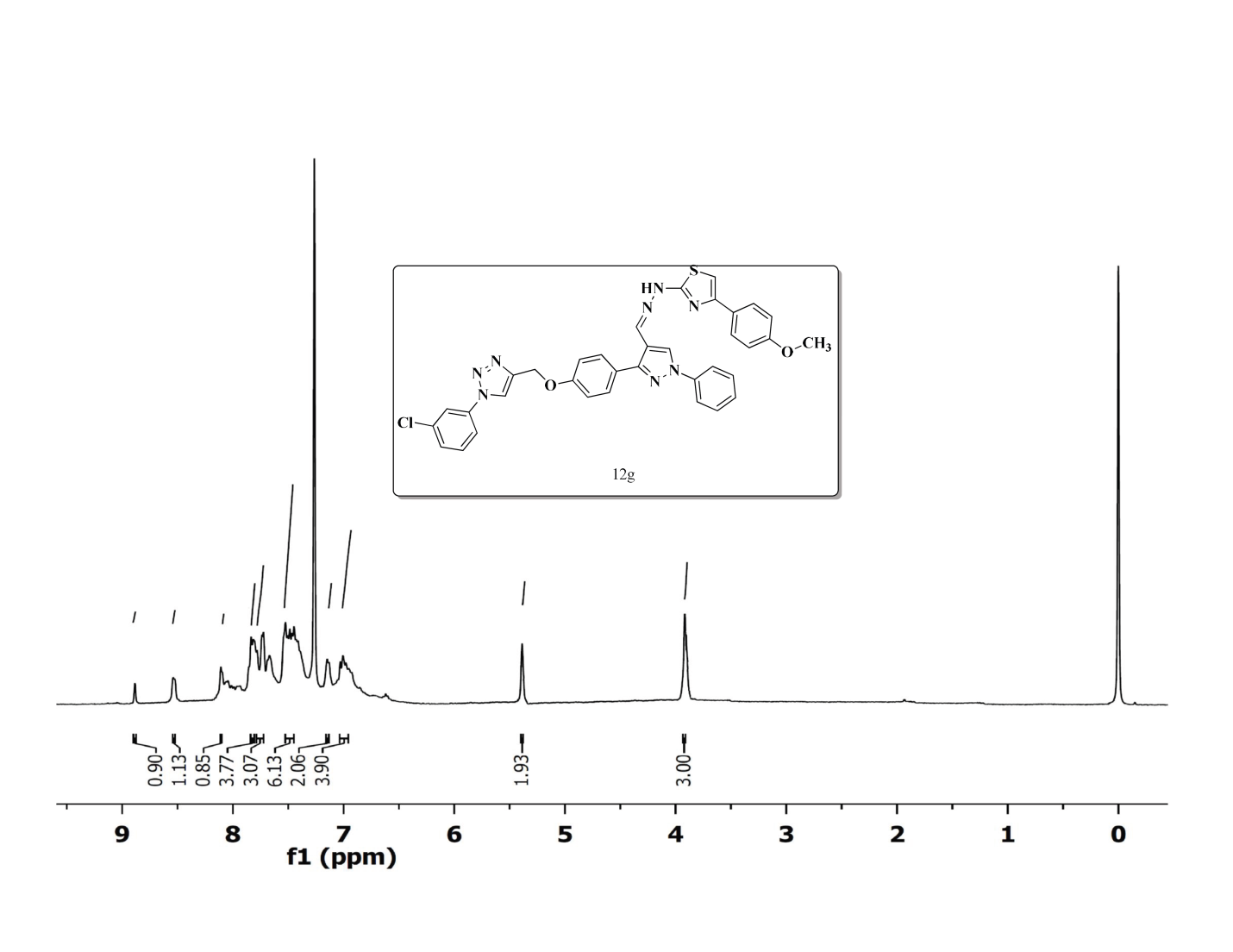
**^1^H NMR of 2-(2-((3-(4-((1-(3-chlorophenyl)-1H-1,2,3-triazol-4-yl)methoxy)phenyl)-1-phenyl-1H-pyrazol-4-yl)methylene)hydrazinyl)-4-(4-methoxyphenyl)thiazole 12g (400 MHz, CDCl_3_)**

**
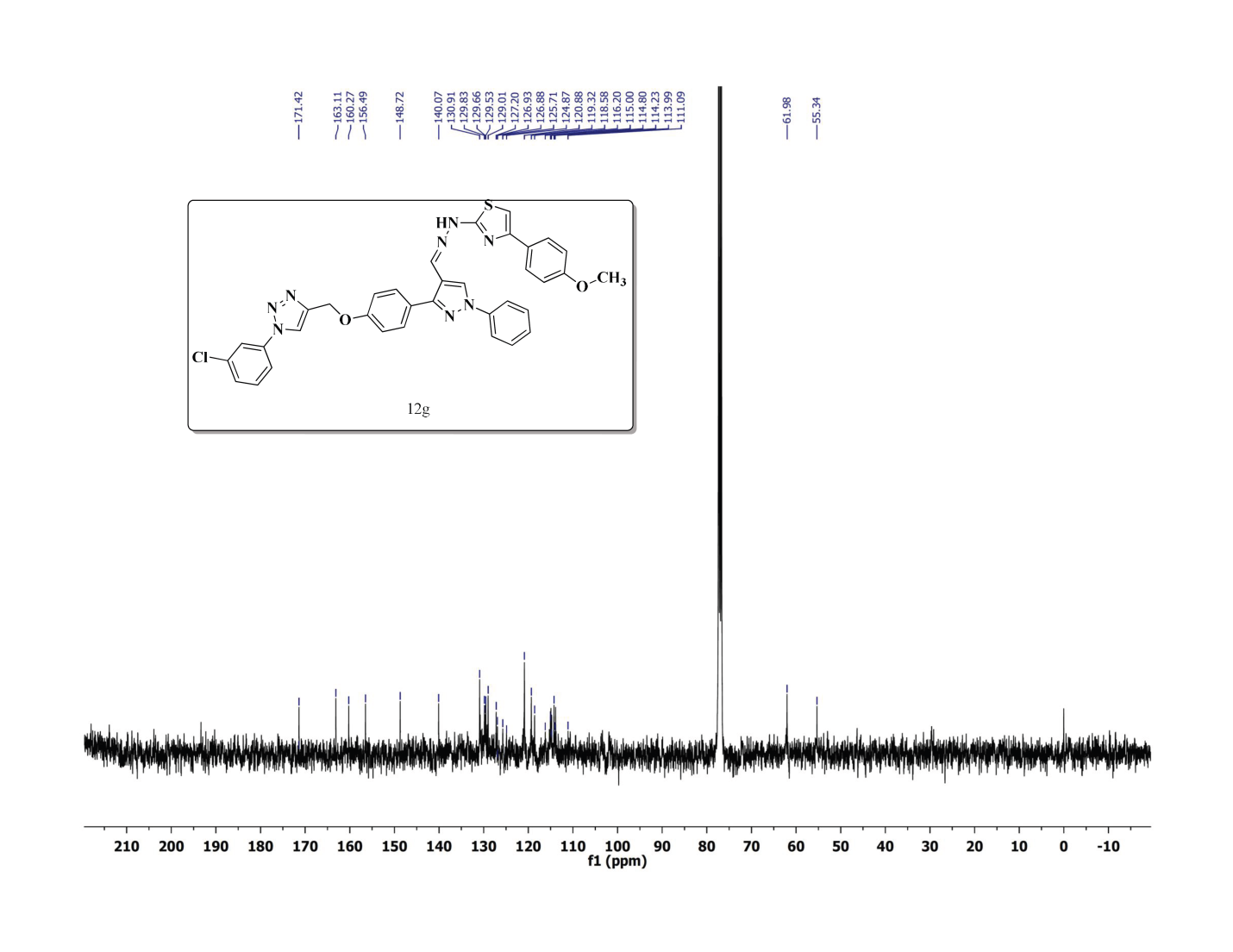
Fig. S32. ^13^C NMR of 2-(2-((3-(4-((1-(3-chlorophenyl)-1H-1,2,3-triazol-4-yl)methoxy)phenyl)-1-phenyl-1H-pyrazol-4-yl)methylene)hydrazinyl)-4-(4-methoxyphenyl)thiazole 10g (100 MHz, CDCl_3_)**


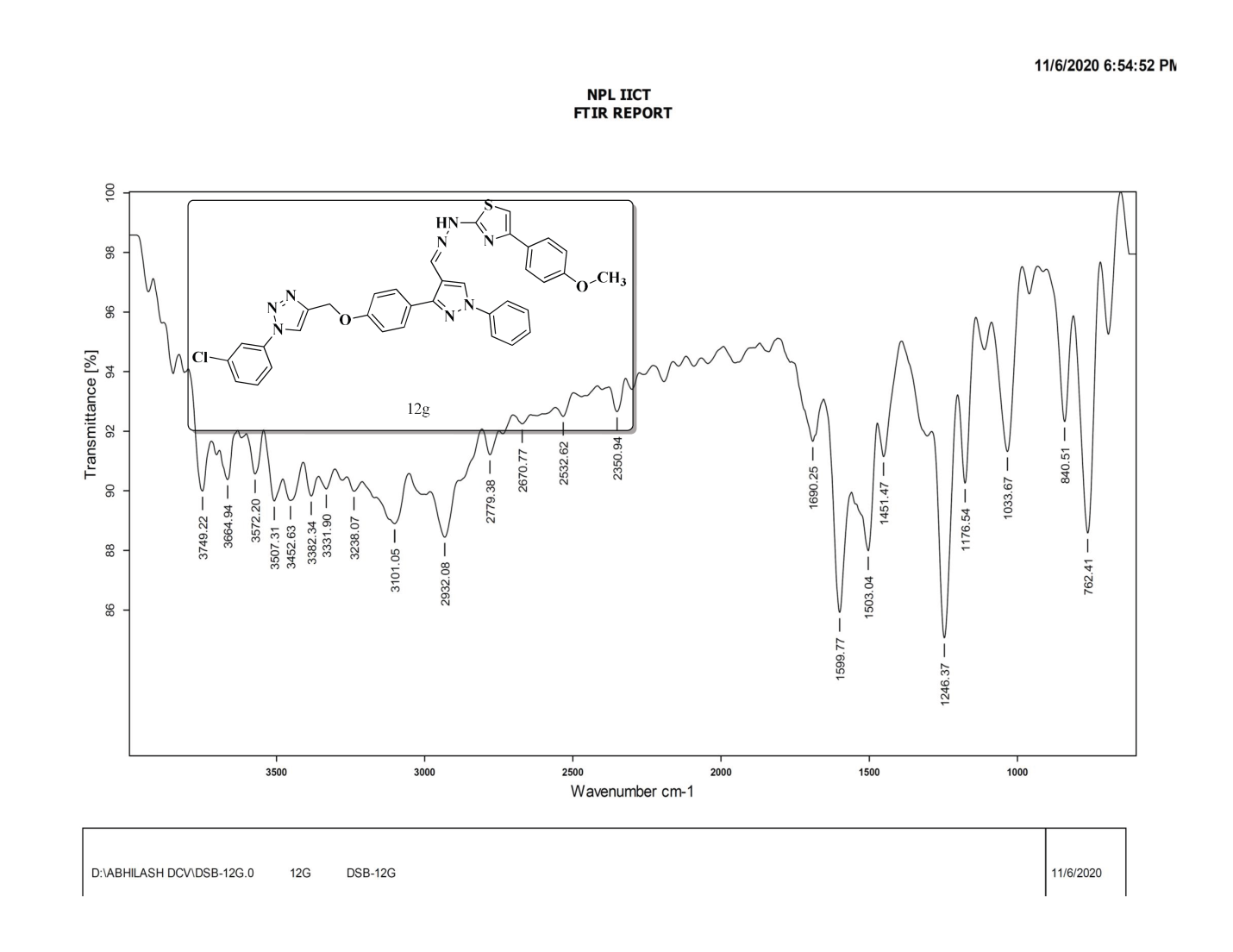


**Fig. S33. FT-IR of 2-(2-((3-(4-((1-(3-chlorophenyl)-1H-1,2,3-triazol-4-yl)methoxy)phenyl)-1-phenyl-1H-pyrazol-4-yl)methylene)hydrazinyl)-4-(4-methoxyphenyl)thiazole 12g**


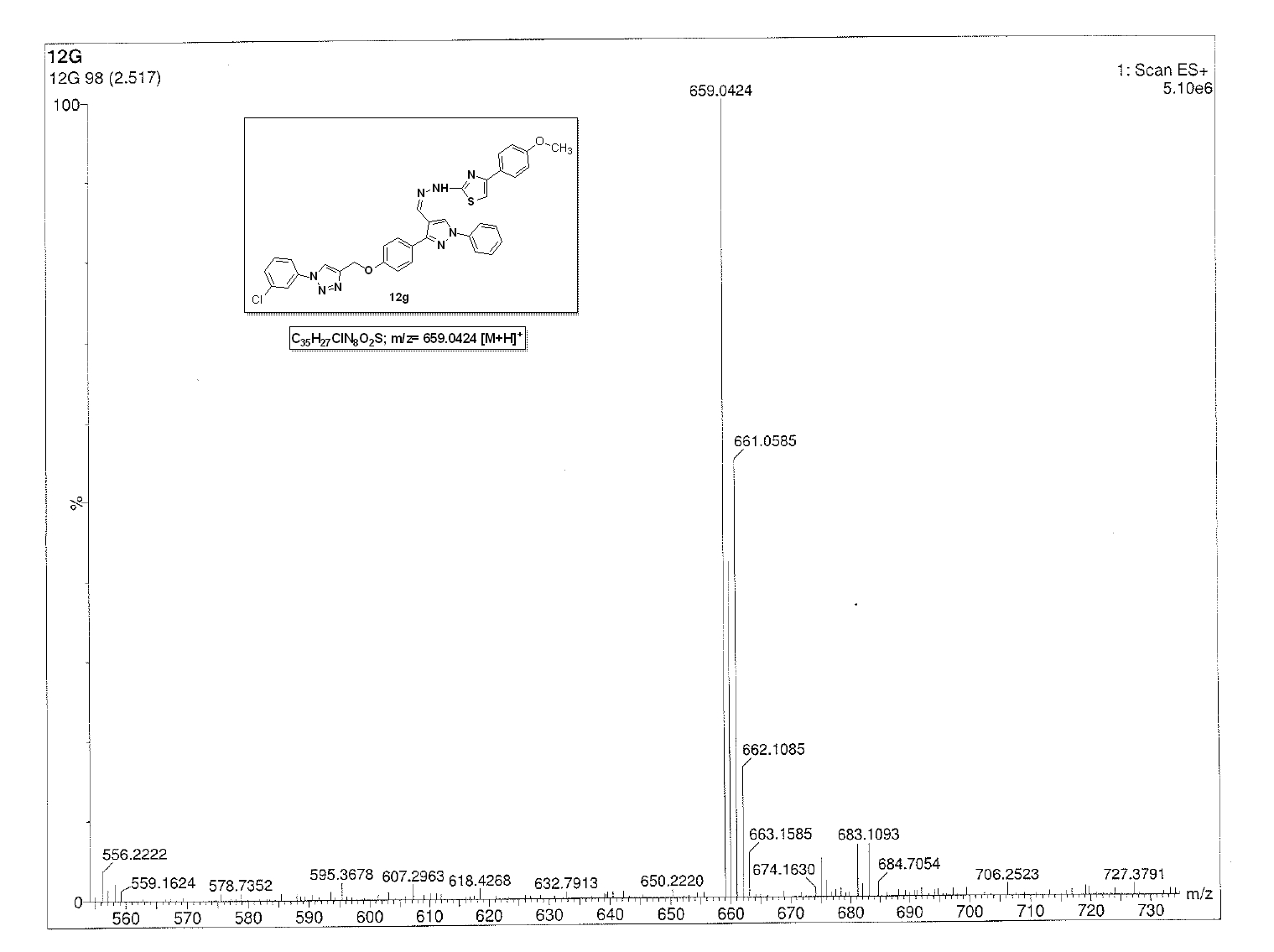


**Fig. S34. ESI-Mass of 2-(2-((3-(4-((1-(3-chlorophenyl)-1H-1,2,3-triazol-4-yl)methoxy)phenyl)-1-phenyl-1H-pyrazol-4-yl)methylene)hydrazinyl)-4-(4-methoxyphenyl)thiazole 12g**


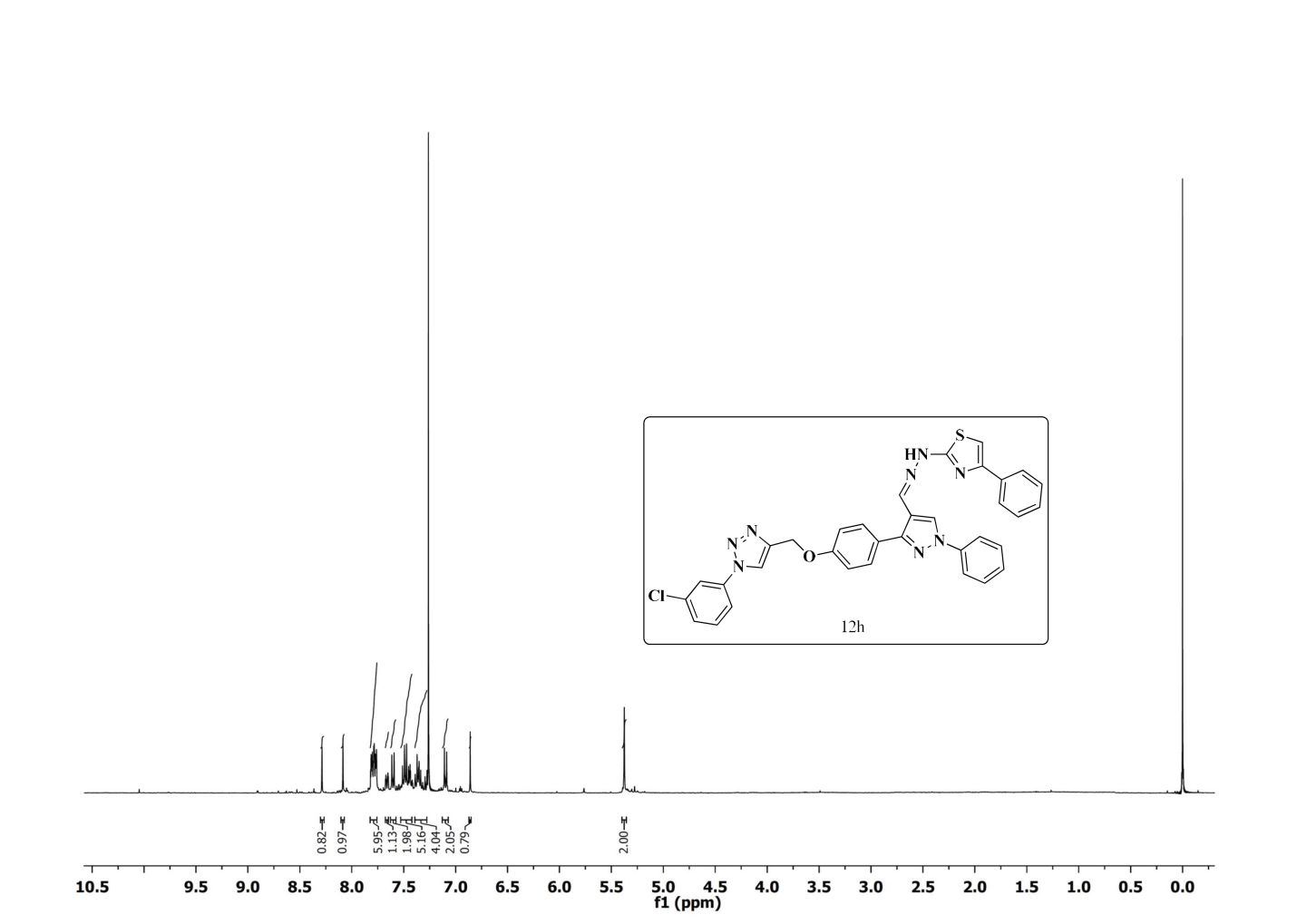


**Fig. S35. ^1^H NMR of 2-(2-((3-(4-((1-(3-chlorophenyl)-1H-1,2,3-triazol-4-yl)methoxy)phenyl)-1-phenyl-1H-pyrazol-4-yl)methylene)hydrazinyl)-4-phenylthiazole 12h (400 MHz, CDCl_3_)**

**
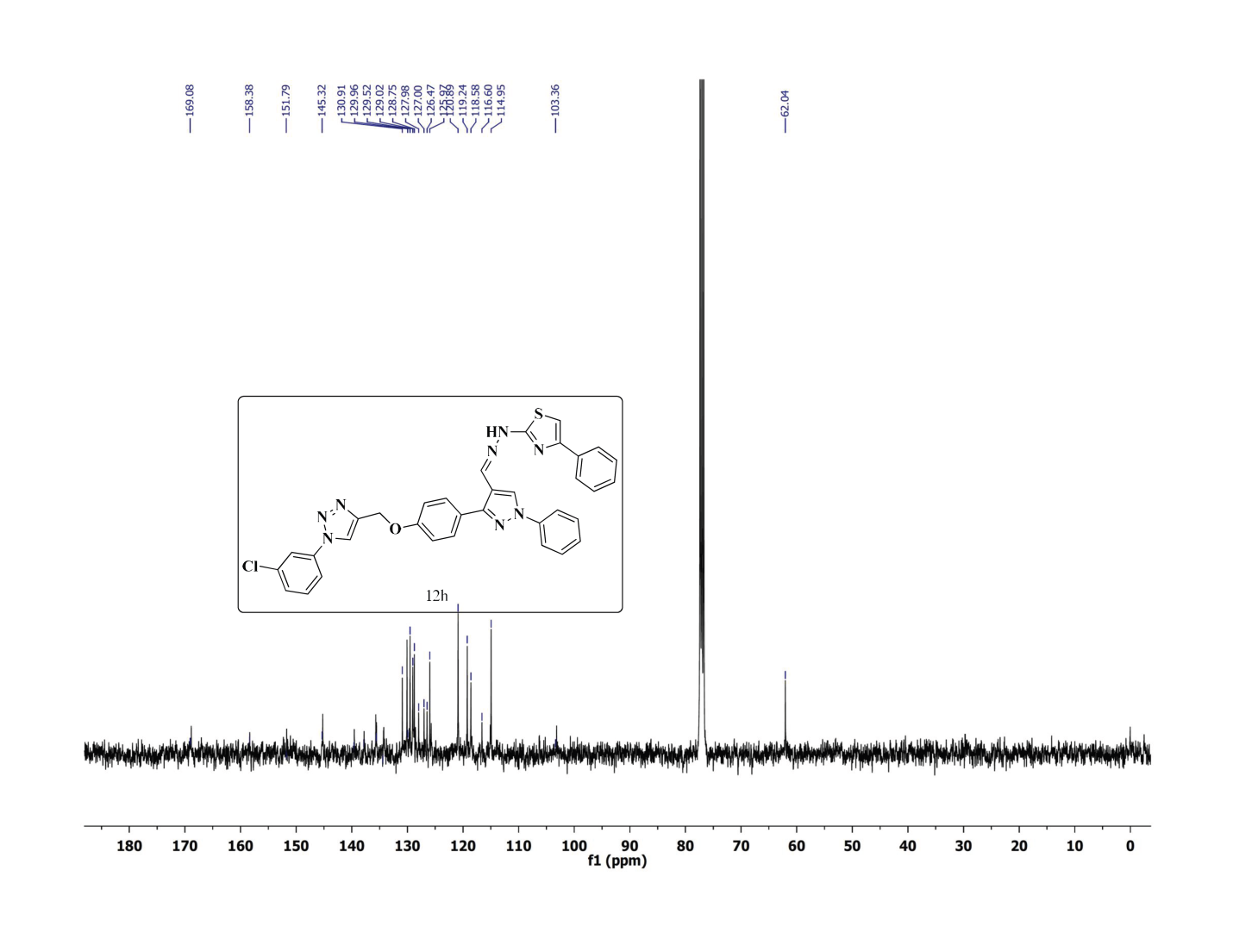
Fig. S36. ^13^C NMR of 2-(2-((3-(4-((1-(3-chlorophenyl)-1H-1,2,3-triazol-4-yl)methoxy)phenyl)-1-phenyl-1H-pyrazol-4-yl)methylene)hydrazinyl)-4-phenylthiazole 12h (100 MHz, CDCl_3_)**

**
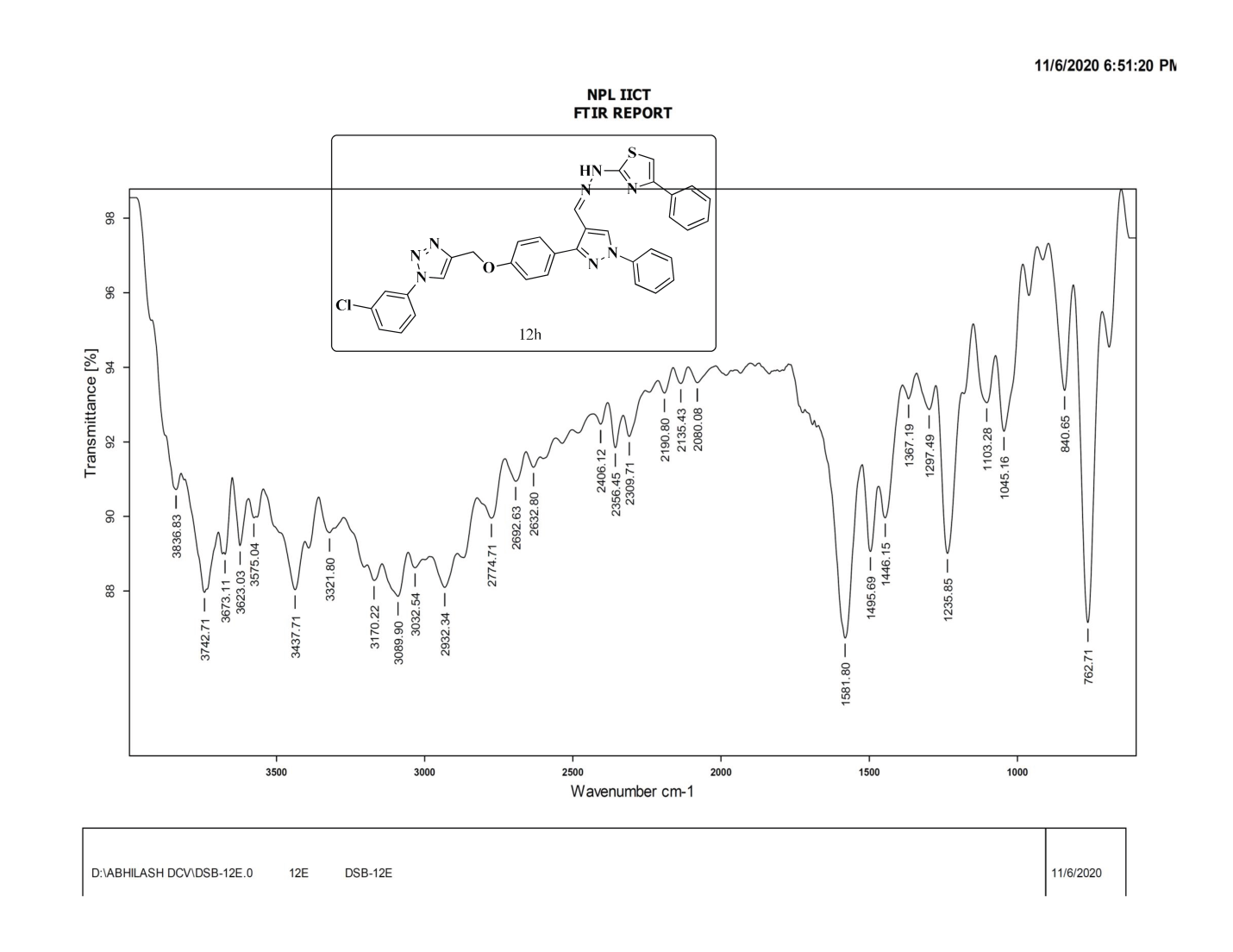
Fig. S37. FT-IR of 2-(2-((3-(4-((1-(3-chlorophenyl)-1H-1,2,3-triazol-4-yl)methoxy)phenyl)-1-phenyl-1H-pyrazol-4-yl)methylene)hydrazinyl)-4-phenylthiazole 10h**


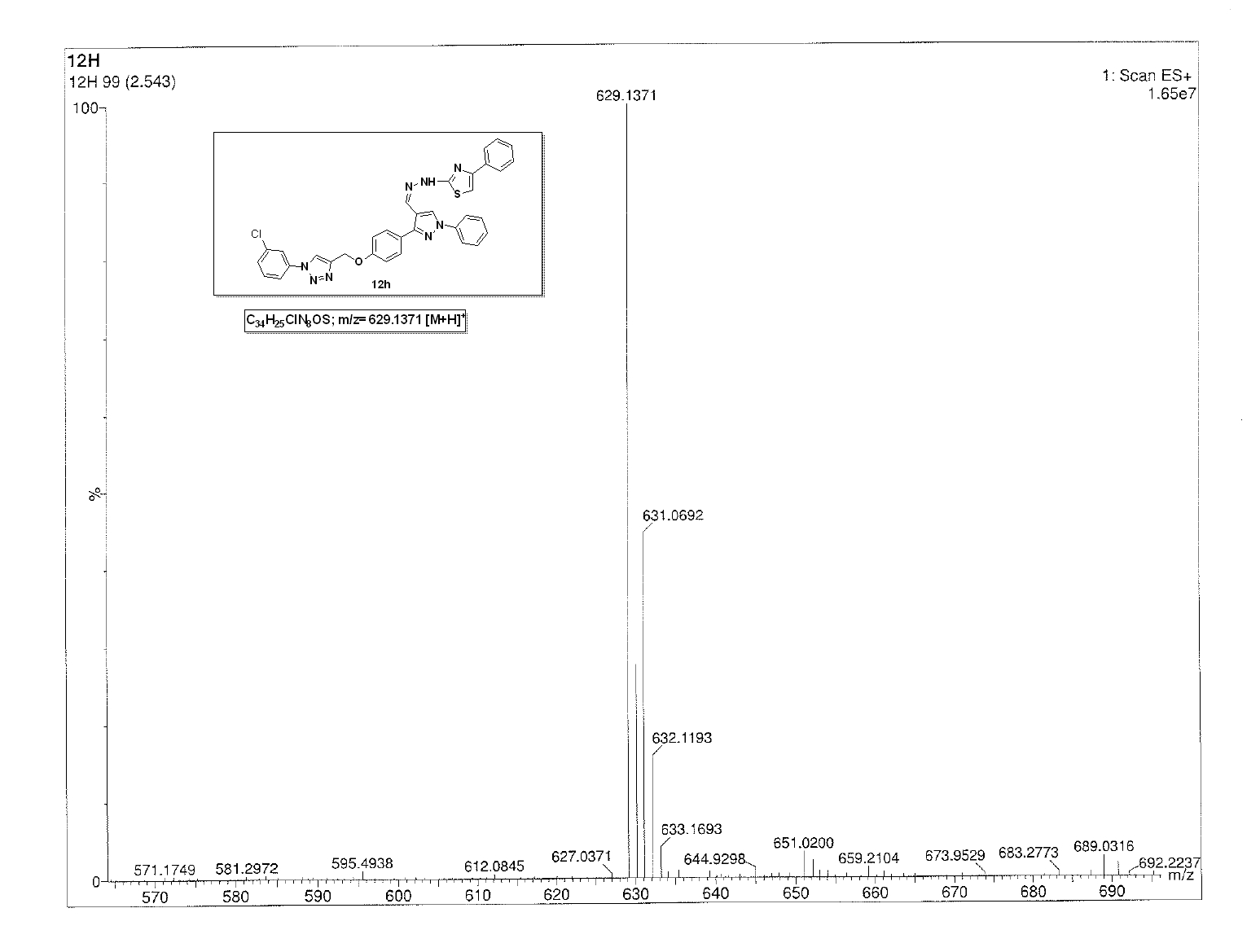


**Fig. S38. ESI-Mass of 2-(2-((3-(4-((1-(3-chlorophenyl)-1H-1,2,3-triazol-4-yl)methoxy)phenyl)-1-phenyl-1H-pyrazol-4-yl)methylene)hydrazinyl)-4-phenylthiazole 12h**

**
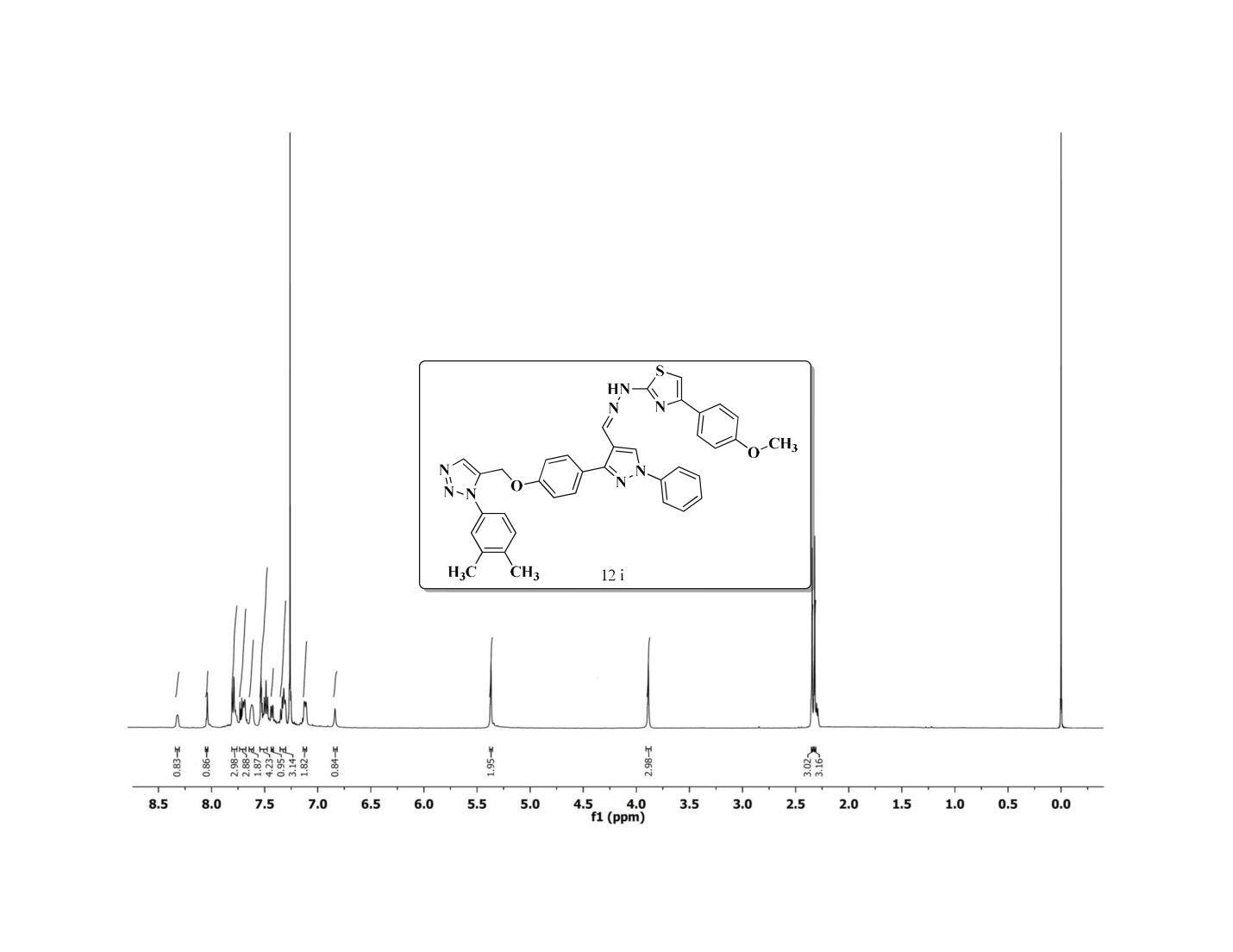
Fig. S39. ^1^H NMR of 2-(2-((3-(4-((1-(3,4-dimethylphenyl)-1H-1,2,3-triazol-4-yl)methoxy)phenyl)-1-phenyl-1H-pyrazol-4-yl)methylene)hydrazinyl)-4-(4-methoxyphenyl)thiazole 12i (400 MHz, CDCl_3_)**


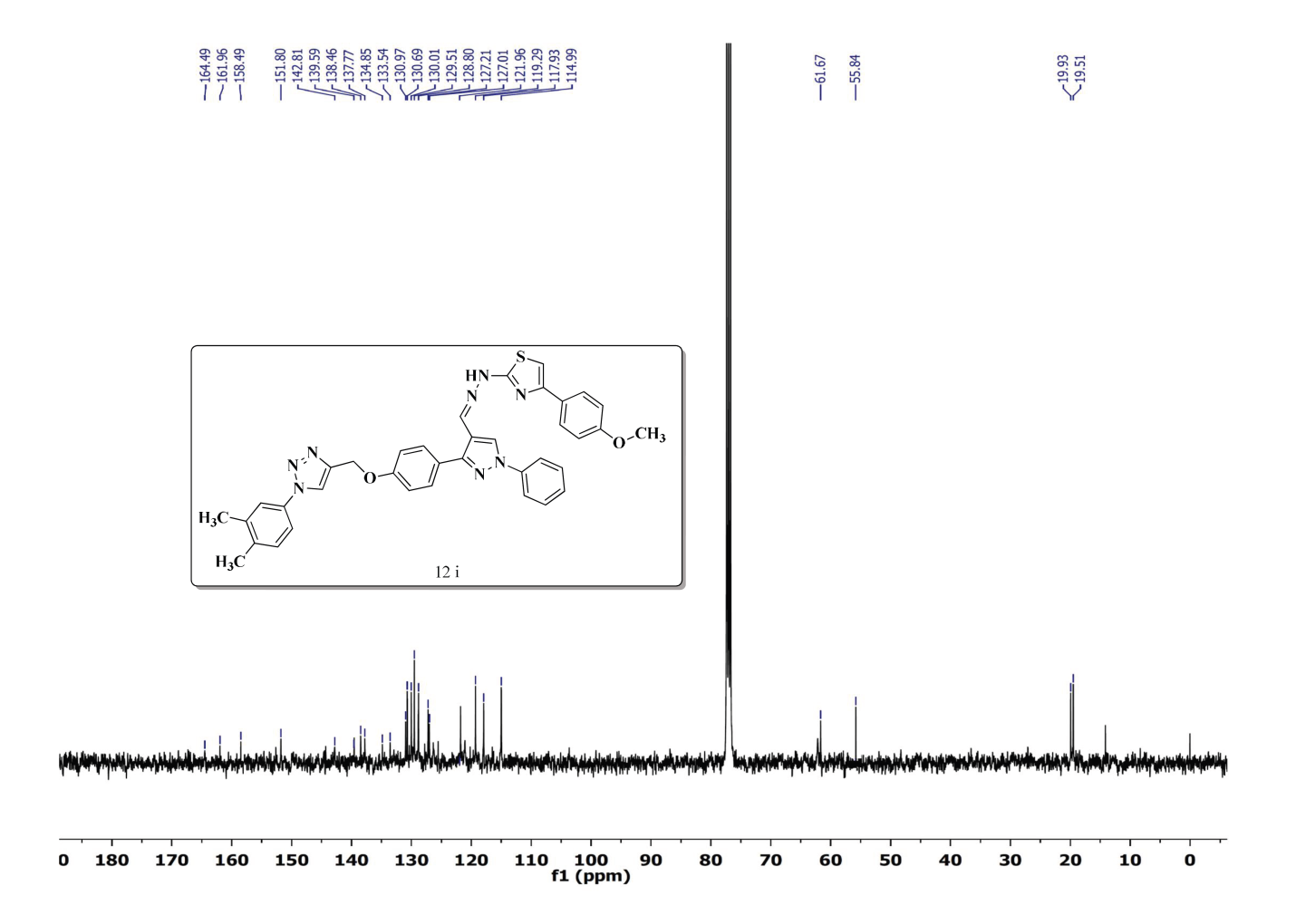


**Fig. S40. ^13^C NMR of 2-(2-((3-(4-((1-(3,4-dimethylphenyl)-1H-1,2,3-triazol-4-yl)methoxy)phenyl)-1-phenyl-1H-pyrazol-4-yl)methylene)hydrazinyl)-4-(4-methoxyphenyl)thiazole 12i (100 MHz, CDCl_3_)**

**
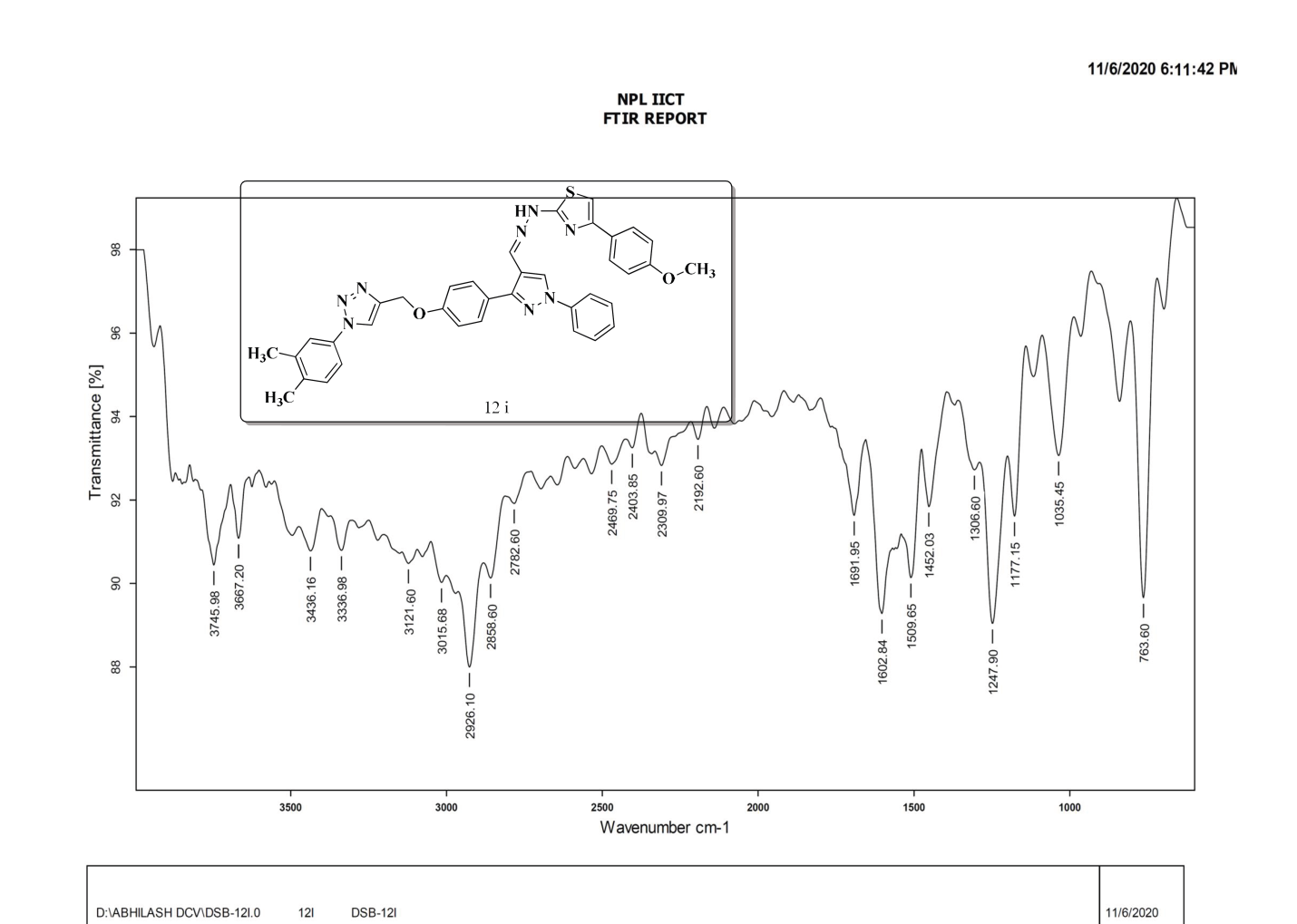
Fig. S41. FT-IR of 2-(2-((3-(4-((1-(3,4-dimethylphenyl)-1H-1,2,3-triazol-4-yl)methoxy)phenyl)-1-phenyl-1H-pyrazol-4-yl)methylene)hydrazinyl)-4-(4-methoxyphenyl)thiazole 10i**


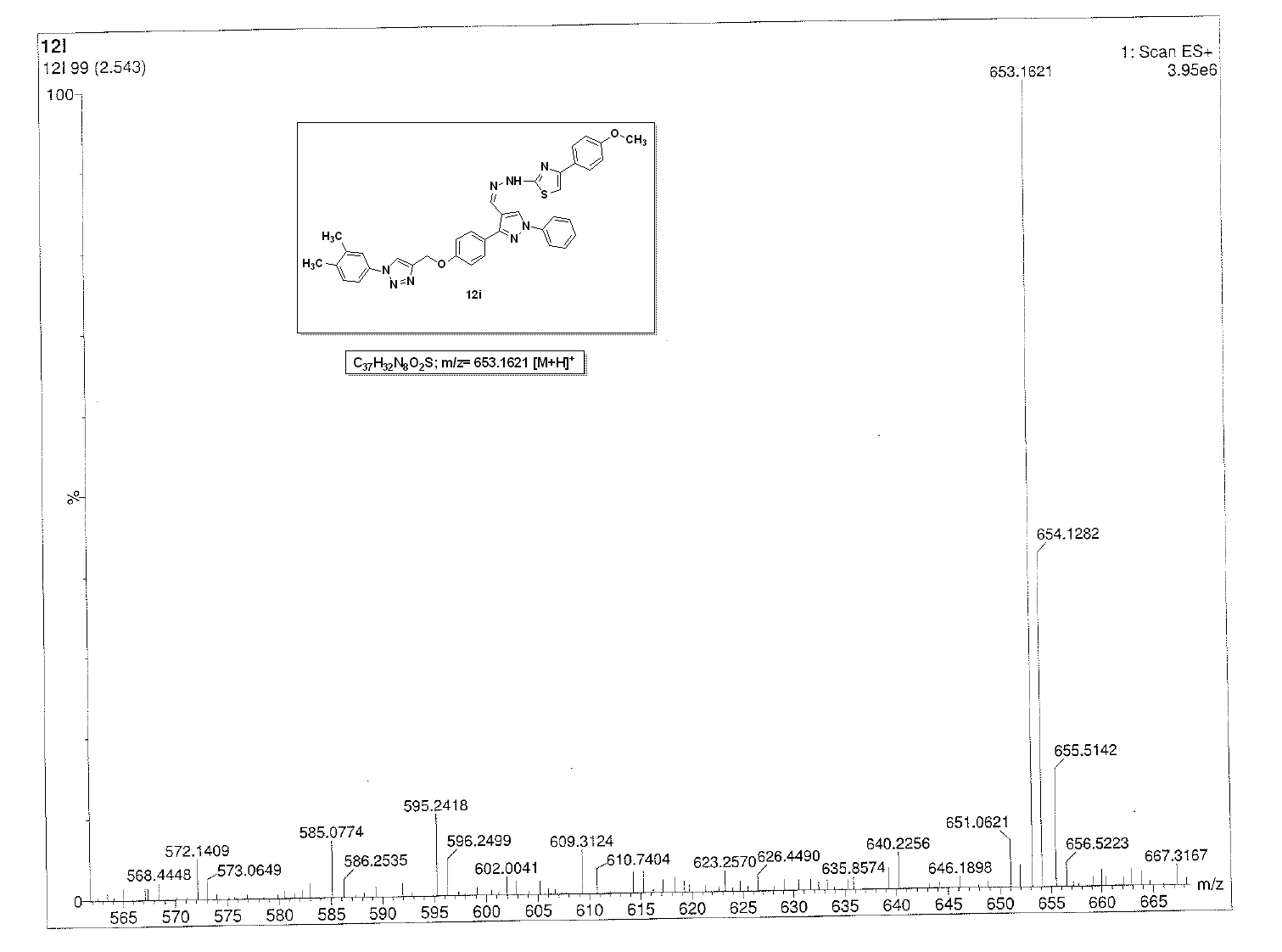


**Fig. S42. ESI-Mass of 2-(2-((3-(4-((1-(3,4-dimethylphenyl)-1H-1,2,3-triazol-4-yl)methoxy)phenyl)-1-phenyl-1H-pyrazol-4-yl)methylene)hydrazinyl)-4-(4-methoxyphenyl)thiazole 12i**

**
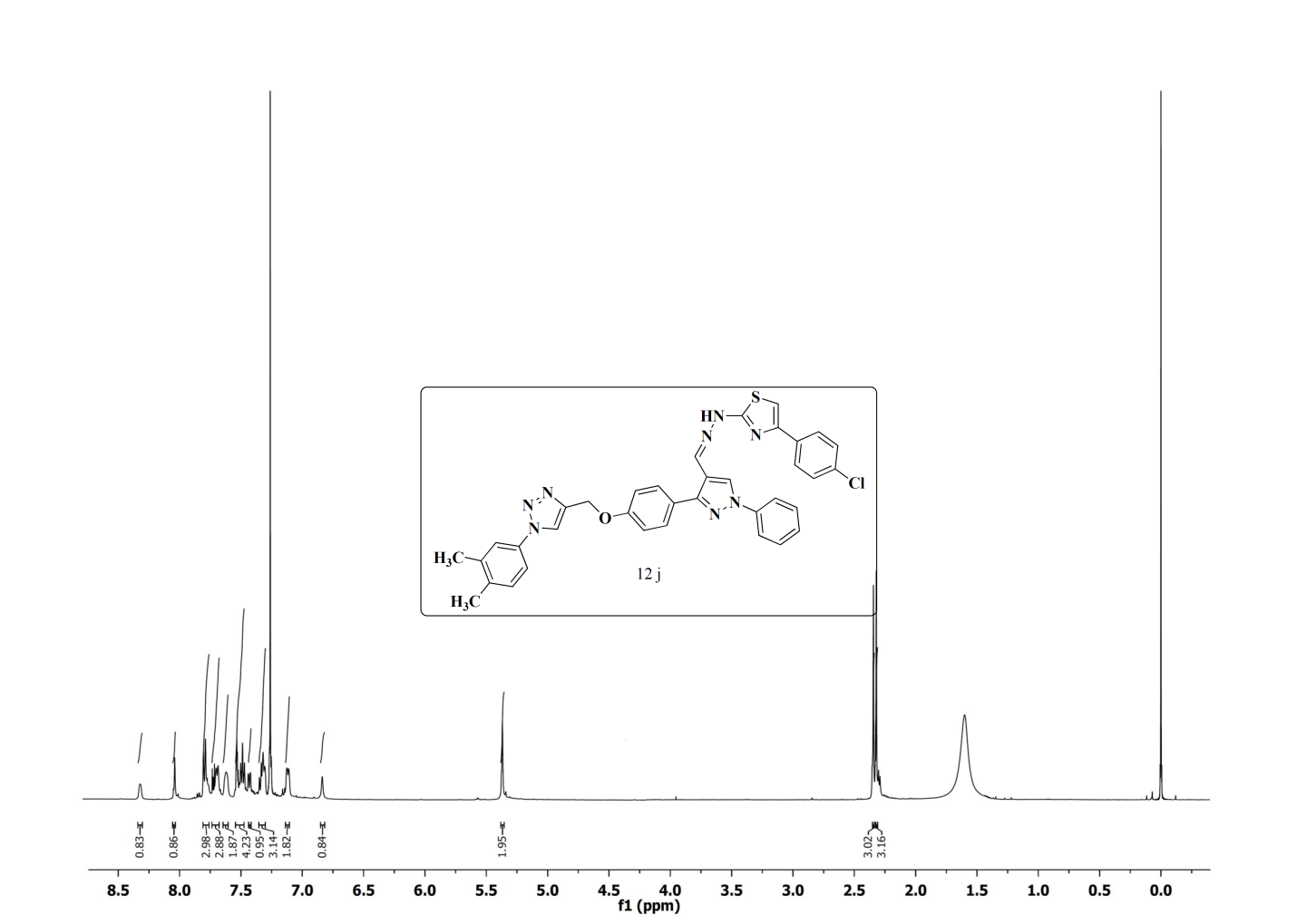
Fig. S43. ^1^H NMR of 4-(4-chlorophenyl)-2-(2-((3-(4-((1-(3,4-dimethylphenyl)-1H-1,2,3-triazol-4-yl)methoxy)phenyl)-1-phenyl-1H-pyrazol-4-yl)methylene)hydrazinyl)thiazole 12j (400 MHz, CDCl_3_)**

**
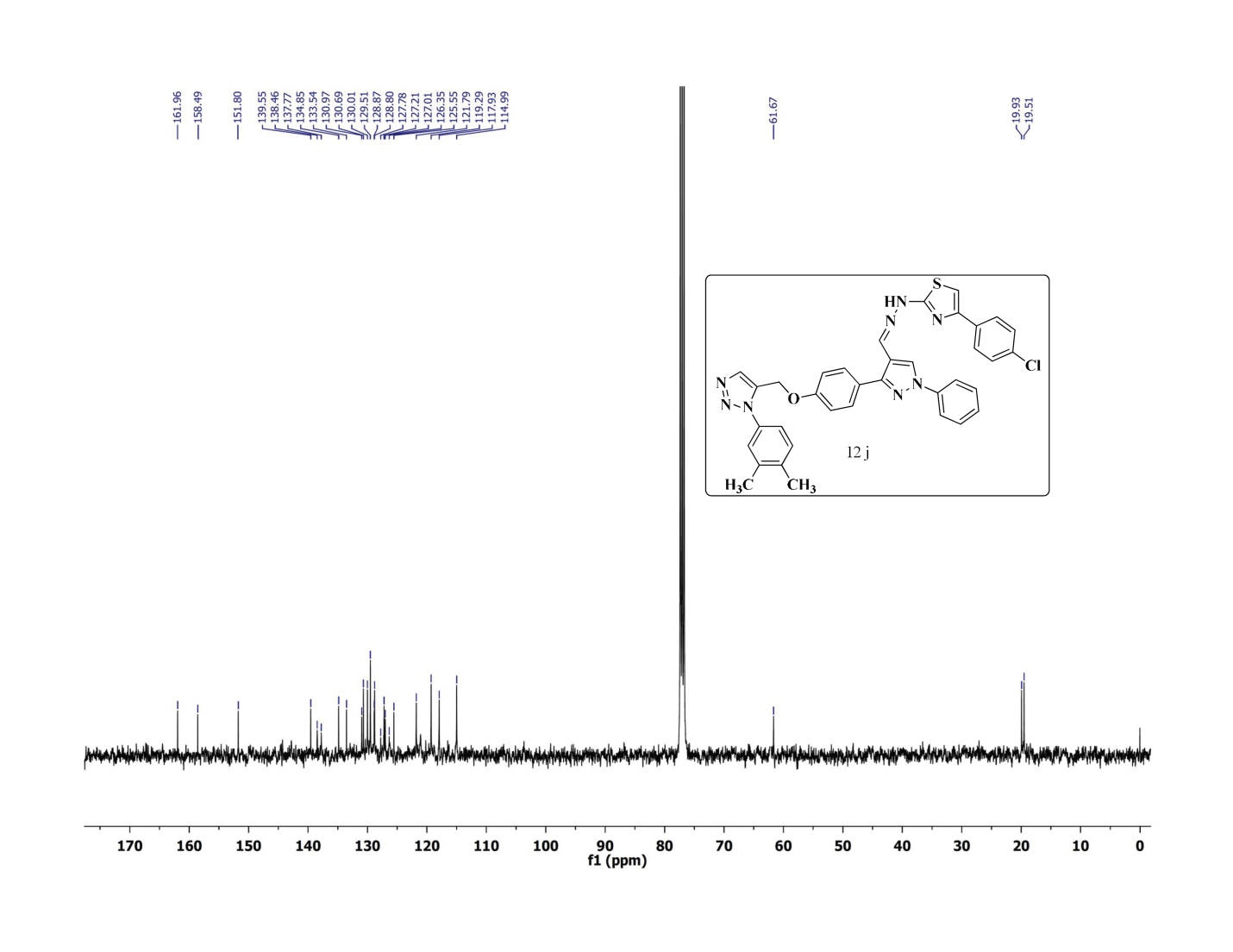
Fig. S44. ^13^C NMR of 4-(4-chlorophenyl)-2-(2-((3-(4-((1-(3,4-dimethylphenyl)-1H-1,2,3-triazol-4-yl)methoxy)phenyl)-1-phenyl-1H-pyrazol-4-yl)methylene)hydrazinyl)thiazole 12j (100 MHz, CDCl_3_)**

**
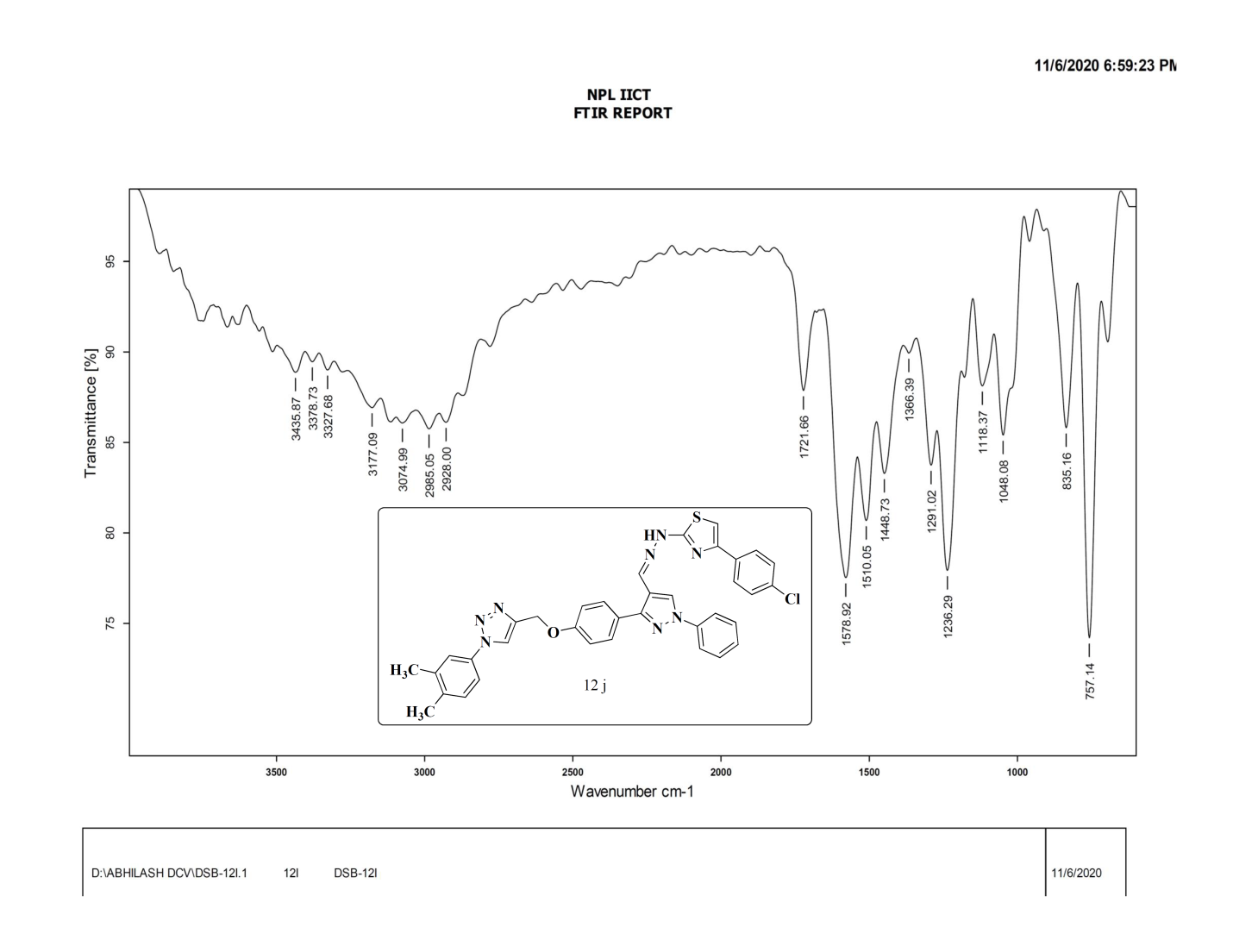
Fig. S45. FT-IR of 4-(4-chlorophenyl)-2-(2-((3-(4-((1-(3,4-dimethylphenyl)-1H-1,2,3-triazol-4-yl)methoxy)phenyl)-1-phenyl-1H-pyrazol-4-yl)methylene)hydrazinyl)thiazole 12j**

**
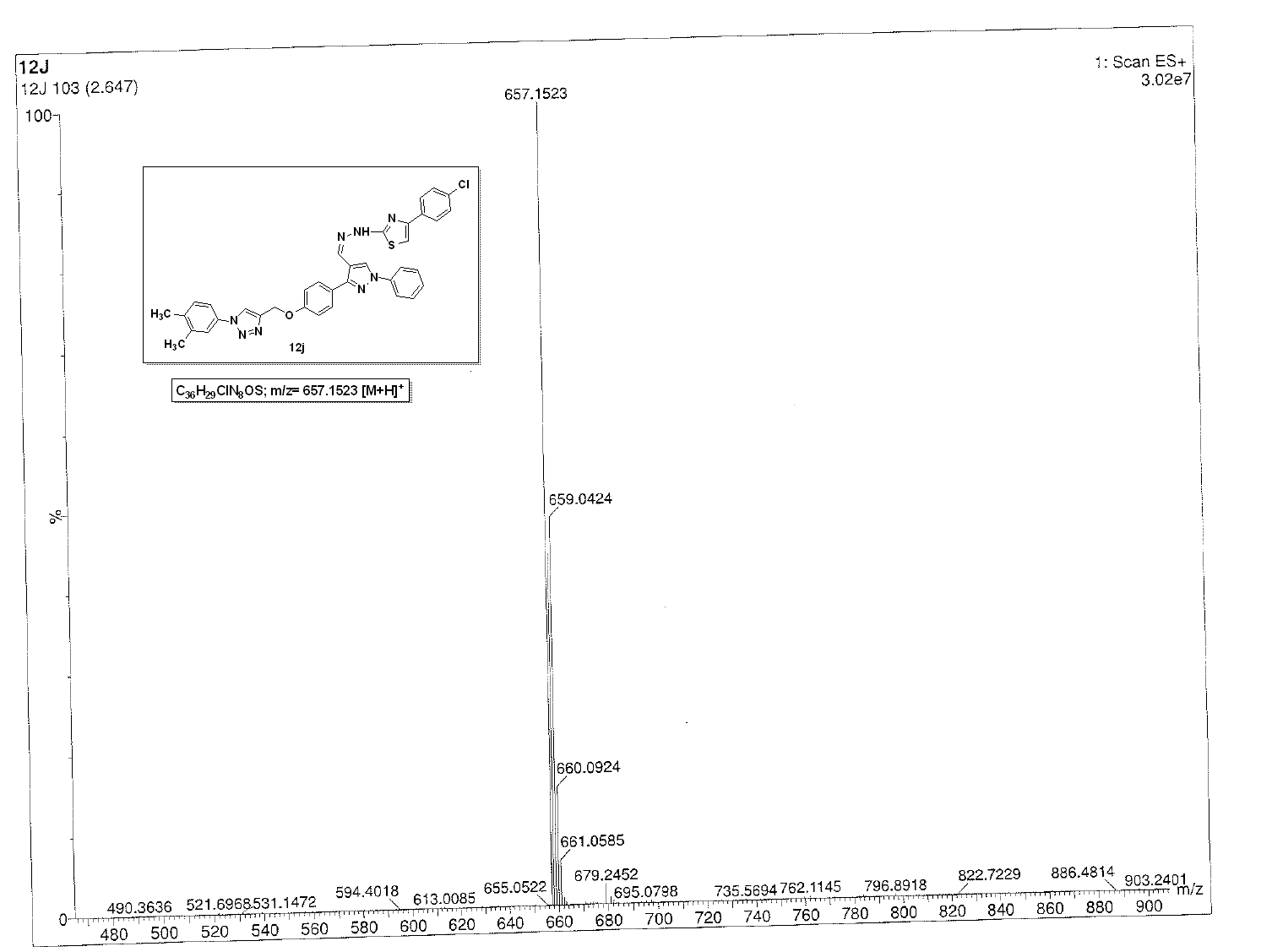
Fig. S46. ESI-Mass of 4-(4-chlorophenyl)-2-(2-((3-(4-((1-(3,4-dimethylphenyl)-1H-1,2,3-triazol-4-yl)methoxy)phenyl)-1-phenyl-1H-pyrazol-4-yl)methylene)hydrazinyl)thiazole 12j**

**
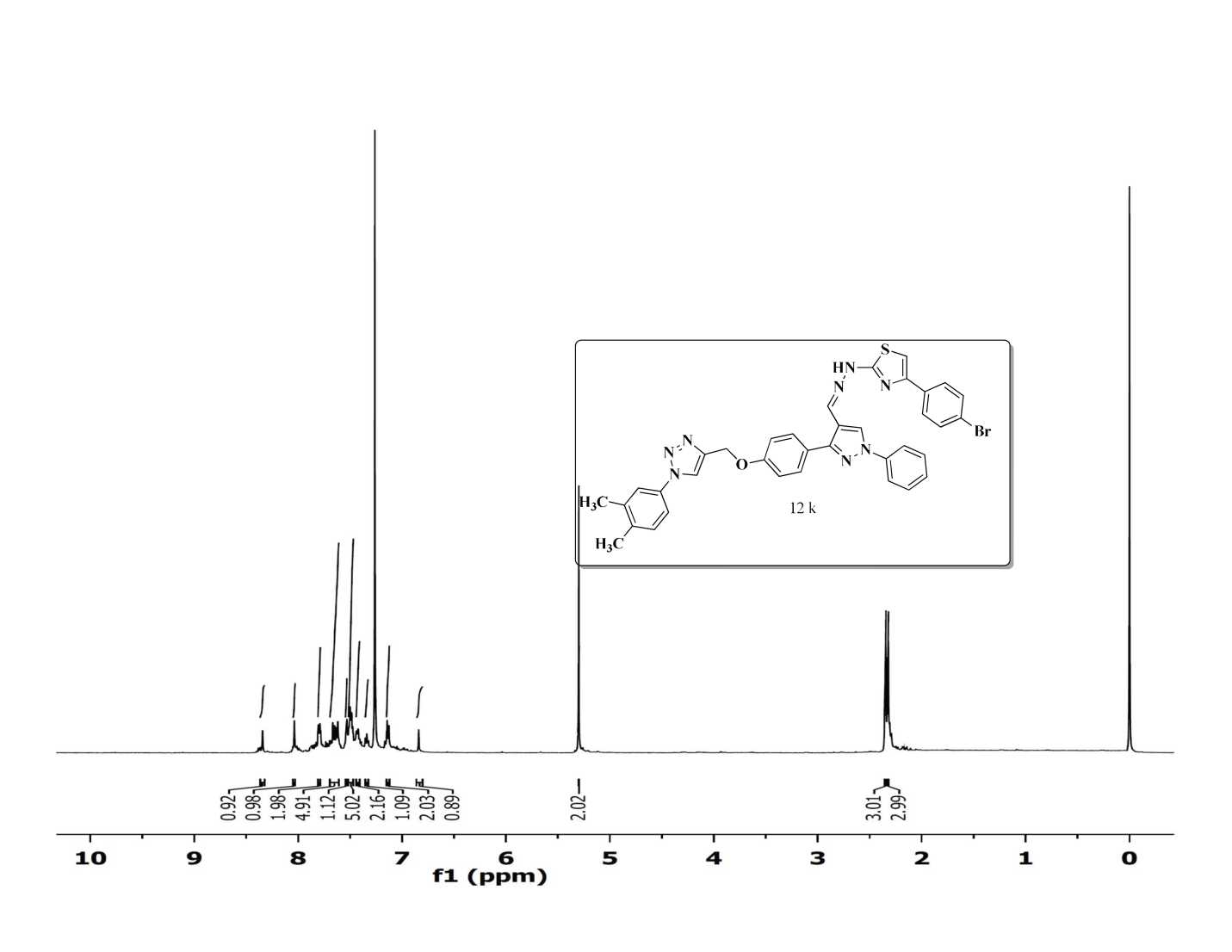
Fig. S47. ^1^H NMR of 4-(4-bromophenyl)-2-(2-((3-(4-((1-(3,4-dimethylphenyl)-1H-1,2,3-triazol-4-yl)methoxy)phenyl)-1-phenyl-1H-pyrazol-4-yl)methylene)hydrazinyl)thiazole 12k (400 MHz, CDCl_3_)**

**
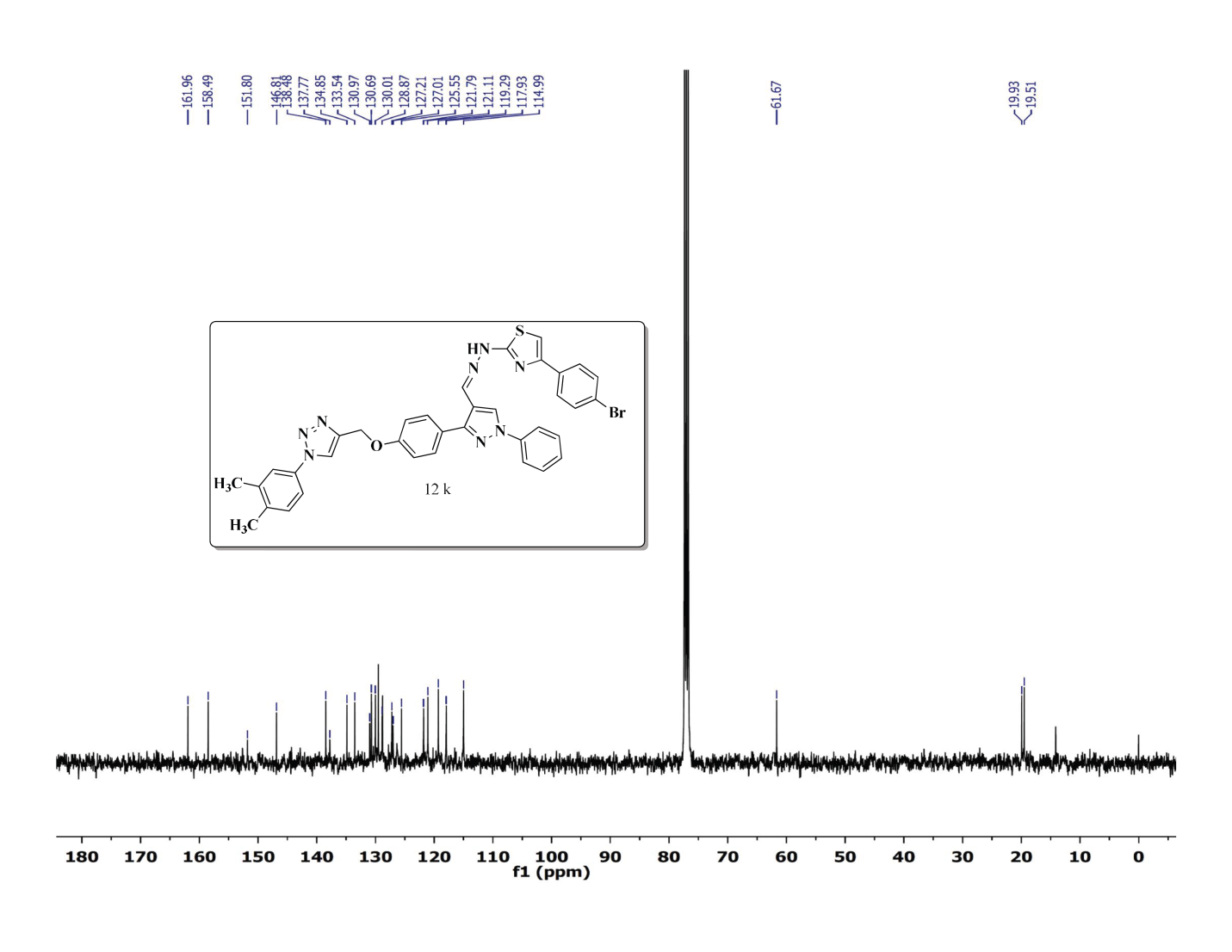
Fig. S48. ^13^C NMR of 4-(4-bromophenyl)-2-(2-((3-(4-((1-(3,4-dimethylphenyl)-1H-1,2,3-triazol-4-yl)methoxy)phenyl)-1-phenyl-1H-pyrazol-4-yl)methylene)hydrazinyl)thiazole 12k (100 MHz, CDCl_3_)**

**
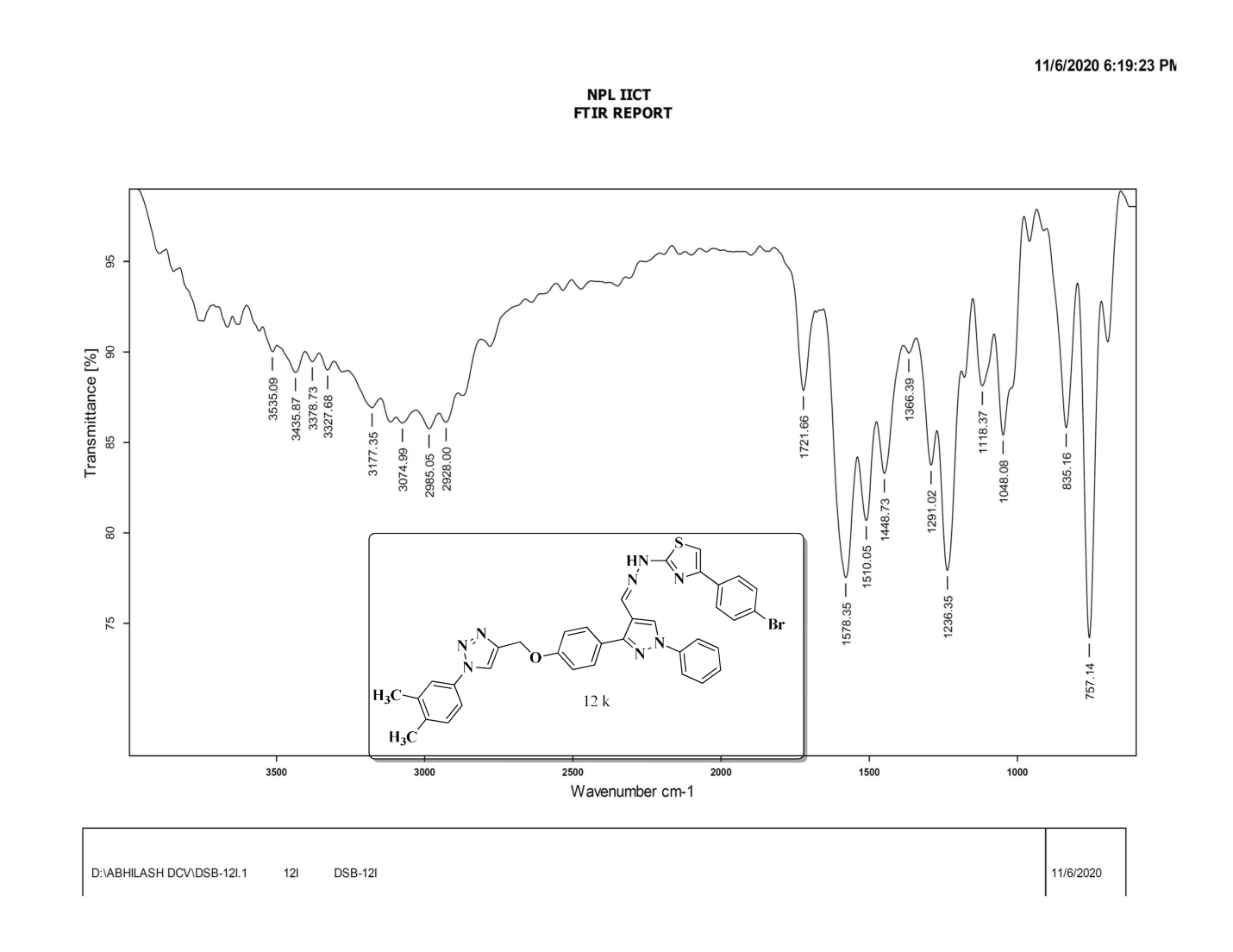
Fig. S49. FT-IR of 4-(4-bromophenyl)-2-(2-((3-(4-((1-(3,4-dimethylphenyl)-1H-1,2,3-triazol-4-yl)methoxy)phenyl)-1-phenyl-1H-pyrazol-4-yl)methylene)hydrazinyl)thiazole 12k**

**
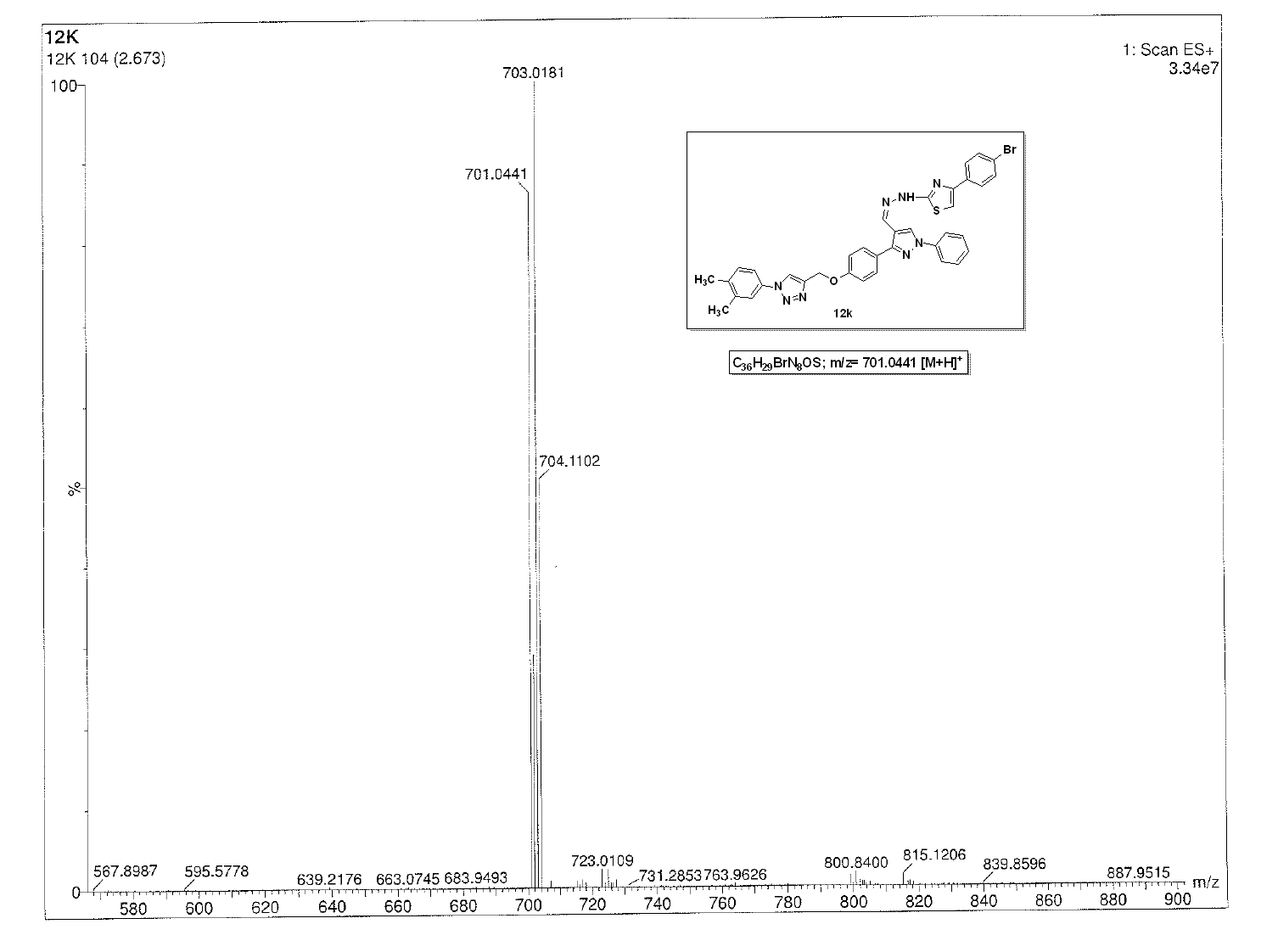
Fig. S50. ESI-Mass of 4-(4-bromophenyl)-2-(2-((3-(4-((1-(3,4-dimethylphenyl)-1H-1,2,3-triazol-4-yl)methoxy)phenyl)-1-phenyl-1H-pyrazol-4-yl)methylene)hydrazinyl)thiazole 12k**

**
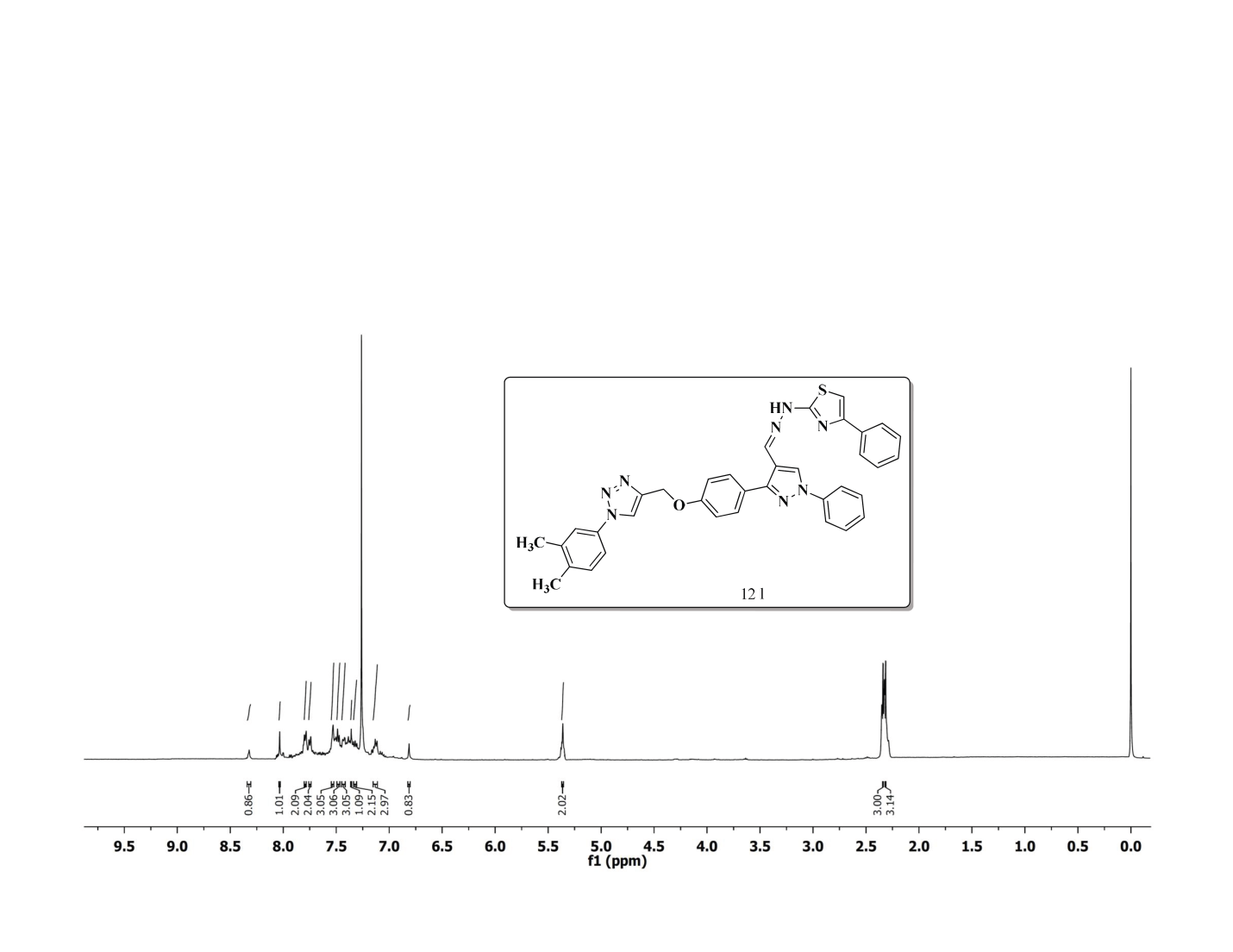
Fig. S51. ^1^H NMR of 2-(2-((3-(4-((1-(3,4-dimethylphenyl)-1H-1,2,3-triazol-4-yl)methoxy)phenyl)-1-phenyl-1H-pyrazol-4-yl)methylene)hydrazinyl)-4-phenylthiazole 12l (400 MHz, CDCl_3_)**

**Fig. S52.**
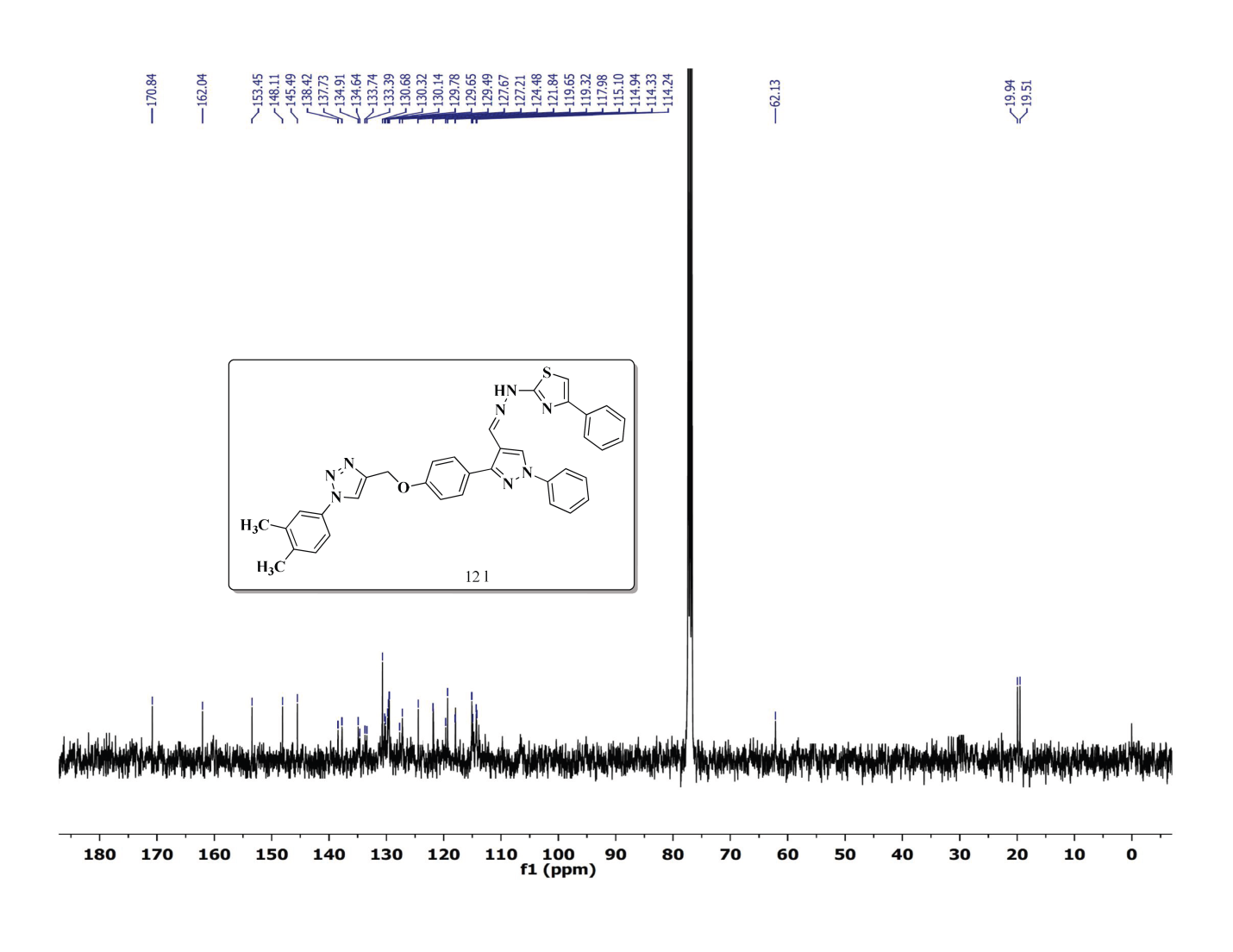
**^13^C NMR of 2-(2-((3-(4-((1-(3,4-dimethylphenyl)-1H-1,2,3-triazol-4-yl)methoxy)phenyl)-1-phenyl-1H-pyrazol-4-yl)methylene)hydrazinyl)-4-phenylthiazole 12l (100 MHz, CDCl_3_)**

**Fig. S53.**
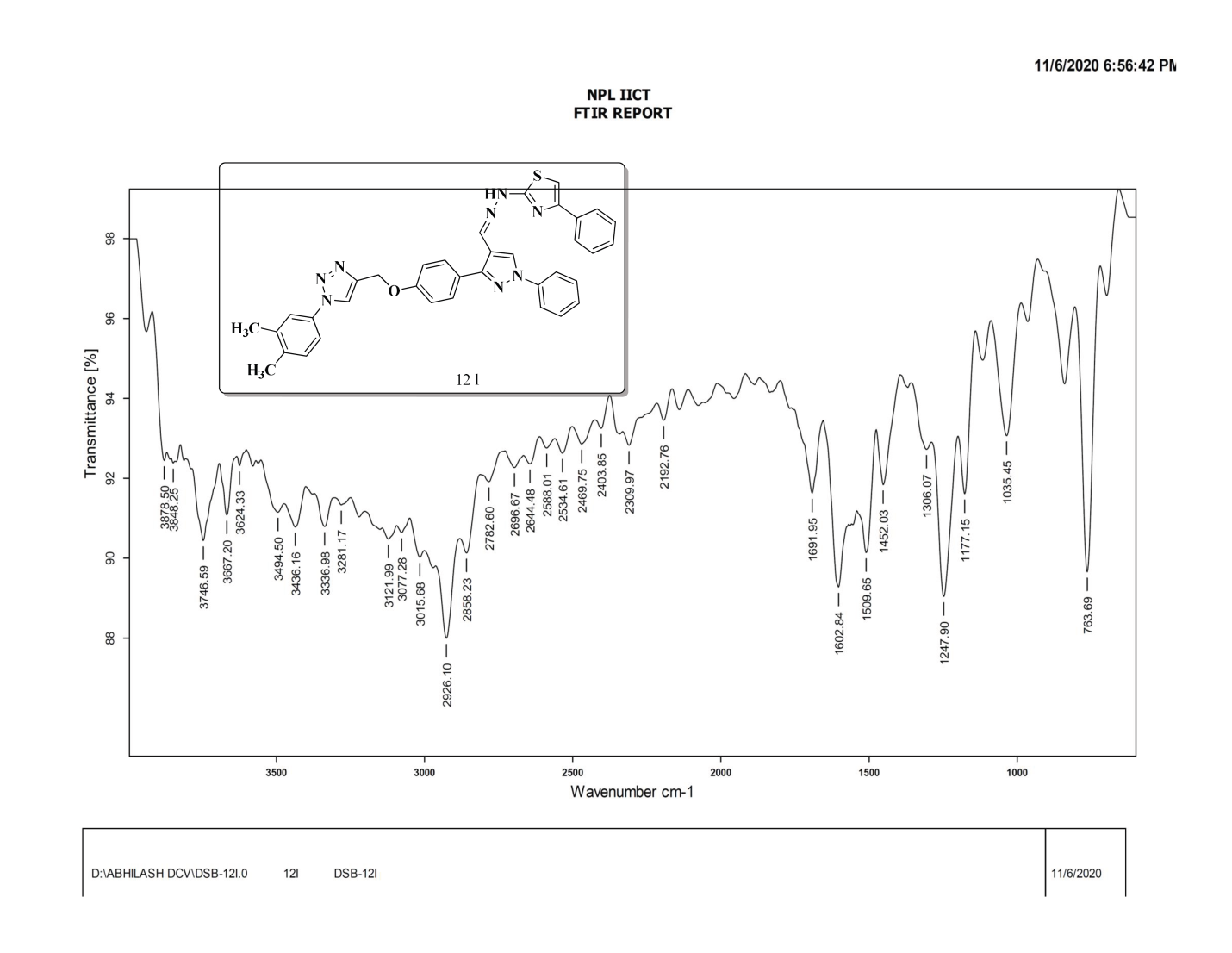
**FT-IR of 2-(2-((3-(4-((1-(3,4-dimethylphenyl)-1H-1,2,3-triazol-4-yl)methoxy)phenyl)-1-phenyl-1H-pyrazol-4-yl)methylene)hydrazinyl)-4-phenylthiazole 12l**

**
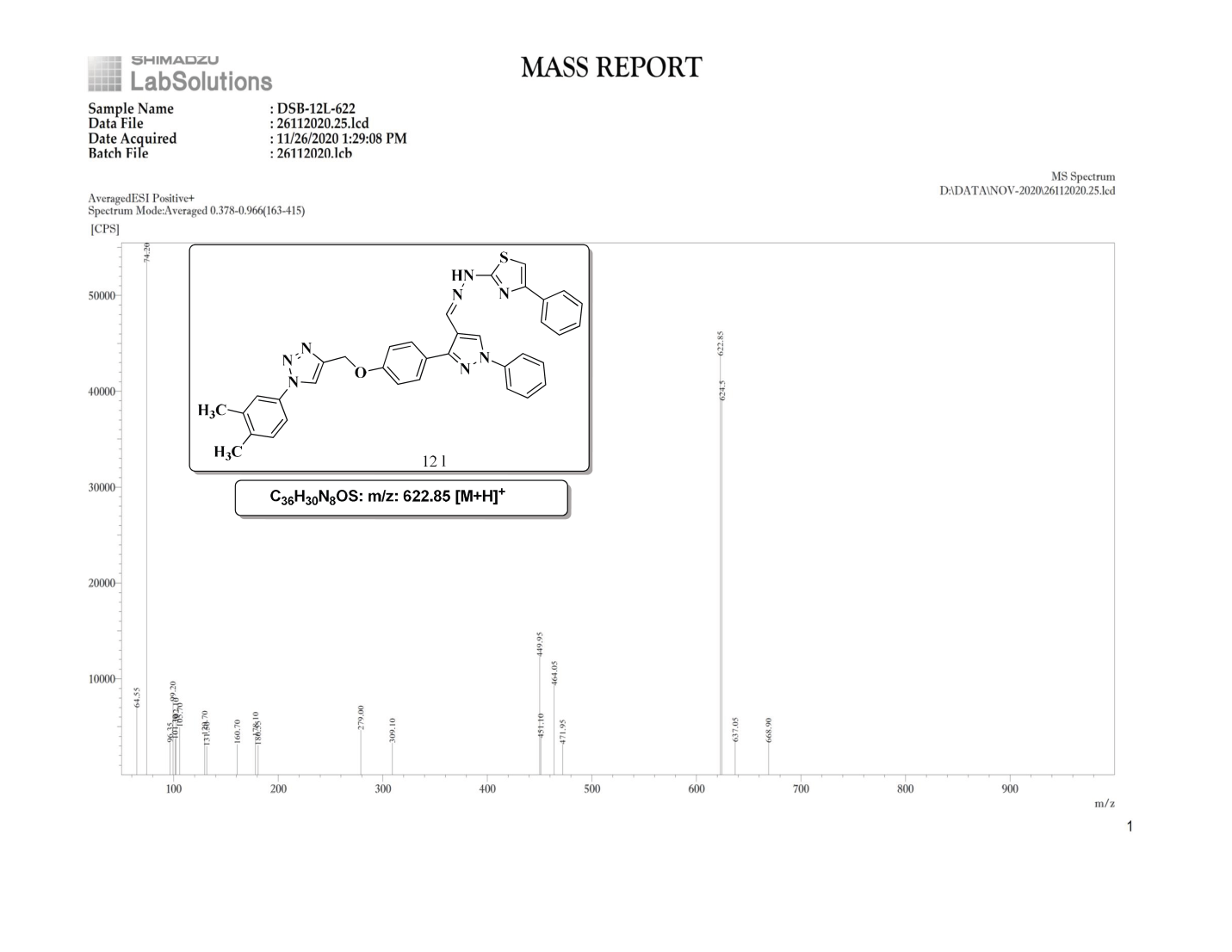
Fig. S54. ESI-Mass of 2-(2-((3-(4-((1-(3,4-dimethylphenyl)-1H-1,2,3-triazol-4-yl)methoxy)phenyl)-1-phenyl-1H-pyrazol-4-yl)methylene)hydrazinyl)-4-phenylthiazole 12l**

**Biological Evaluation**

**Additional information for present antibacterial assays:**

**Provided here are the raw data for works carried in present study.**

**Table S1** MIC of synthesized **12a-l**compounds (μg/ml) against gram-positive and gram-negative bacterial strains

Initially we performed zone of inhibition studies then we confirmed it with MIC inhibitory assays.

**Table S1a Table S1b**

| **Compound** | *S. aureus* MTCC 96 | | | |
| --- | --- | --- | --- | --- |
|  |  |  |  |  |
|  | 1 | 2 | 3 | MIC |
| **12a** | 6.7 | 6.6 | 6.7 | 6.666666667 |
| **12b** | 7.1 | 7.2 | 7.2 | 7.166666667 |
| **12e** | 4.7 | 4.8 | 4.8 | 4.766666667 |
| **12f** | 5 | 5.2 | 5.1 | 5.1 |
| **12g** | 7.9 | 8 | 8 | 7.966666667 |
| **12h** | 7.2 | 7.1 | 7.2 | 7.166666667 |
| **12i** | 9.8 | 9.8 | 9.7 | 9.766666667 |
| **12j** | 8.7 | 8.8 | 8.8 | 8.766666667 |
| **12k** | 4.1 | 4.1 | 4.2 | 4.133333333 |
| **12l** | 9.5 | 9.5 | 9.4 | 9.466666667 |
| *Novobiocin* | 3.9 | 3.9 | 3.8 | 3.866666667 |
| *Ampicillin* | 10 | 10.1 | 10 | 10.03333333 |

| **Compound** | *B. subtilis* MTCC 441 | | | |
| --- | --- | --- | --- | --- |
|  |  |  |  |  |
|  | 1 | 2 | 3 | MIC |
| **12a** | 6.7 | 6.6 | 6.7 | 6.666666667 |
| **12b** | 6.1 | 6.2 | 6.1 | 6.133333333 |
| **12e** | 6.2 | 6.2 | 6.2 | 6.2 |
| **12f** | 6.2 | 6.2 | 6.2 | 6.2 |
| **12g** | 7.2 | 7.2 | 7.2 | 7.2 |
| **12h** | 8 | 8 | 8 | 8 |
| **12i** | 7.2 | 7.2 | 7.2 | 7.2 |
| **12j** | 8 | 8 | 8 | 8 |
| **12k** | 6.2 | 6.2 | 6.2 | 6.2 |
| **12l** | 6.2 | 6.2 | 6.2 | 6.2 |
| *Ampicillin* | 10.1 | 10.1 | 10 | 10.06666667 |

**Table S1c**

| **Compound** | *P. aeruginosa* MTCC 424 | | | |
| --- | --- | --- | --- | --- |
|  |  |  |  |  |
|  | 1 | 2 | 3 | MIC |
| **12a** | 9.6 | 9.5 | 9.5 | 9.533333333 |
| **12b** | 9.8 | 9.8 | 9.7 | 9.766666667 |
| **12e** | 9.8 | 9.8 | 9.7 | 9.766666667 |
| **12f** | 8.7 | 8.8 | 8.6 | 8.7 |
| **12g** | 9.8 | 9.8 | 9.9 | 9.833333333 |
| **12h** | 9.5 | 9.5 | 9.6 | 9.533333333 |
| **12i** | 9.8 | 9.8 | 9.9 | 9.833333333 |
| **12j** | 10 | 10 | 10.1 | 10.03333333 |
| **12k** | 9.5 | 9.5 | 9.6 | 9.533333333 |
| **12l** | 9.5 | 9.6 | 9.5 | 9.533333333 |
| *Ampicillin* | 10.1 | 10 | 10.1 | 10.06666667 |

**Table S1d**

| **Compound** | *E. coli* MTCC 443 | | | |
| --- | --- | --- | --- | --- |
|  |  |  |  |  |
|  | 1 | 2 | 3 | MIC |
| **12a** | 10.1 | 10.1 | 10 | 10.06666667 |
| **12b** | 10.1 | 10 | 10 | 10.03333333 |
| **12e** | 10.1 | 10 | 10 | 10.03333333 |
| **12f** | 10 | 10.1 | 10 | 10.03333333 |
| **12g** | 10 | 10.1 | 10 | 10.03333333 |
| **12h** | 10.1 | 10 | 10 | 10.03333333 |
| **12i** | >50 | >50 | >50 | >50 |
| **12j** | >25 | >25 | >25 | >25 |
| **12k** | **>25** | **>25** | **>25** | **>25** |
| **12l** | >50 | >50 | >50 | >50 |
| *Ampicillin* | 3.8 | 3.9 | 3.9 | 3.866666667 |


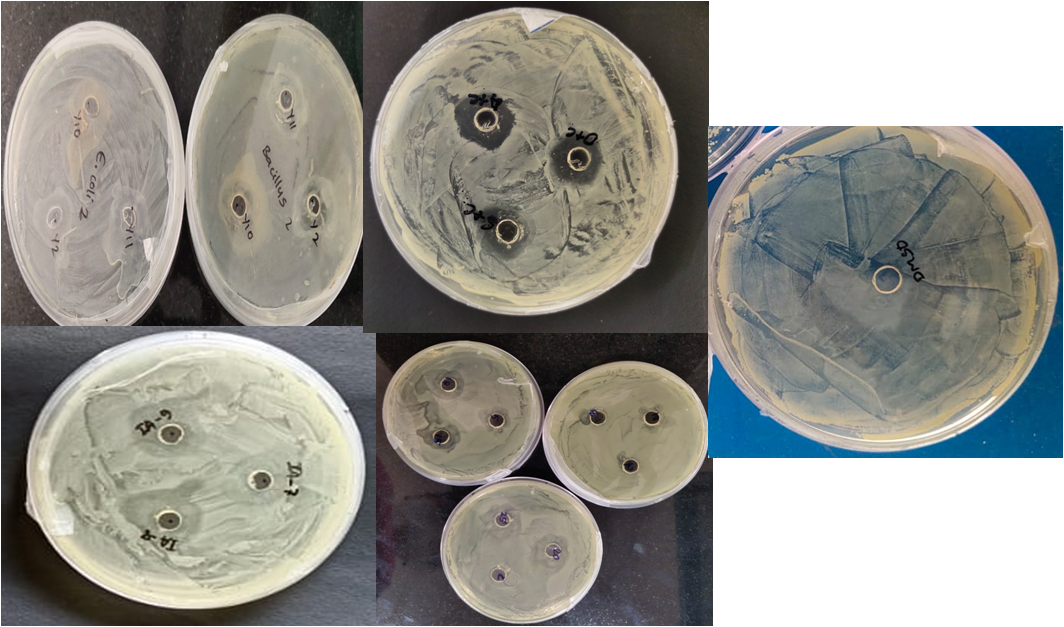


Fig. S55**.** Zone of inhibition test for antimicrobial activity

**Raw Data of Docking** [**Click Here**](https://drive.google.com/file/d/1E4bU2ANXD_kYy-tqjZr4gAHh7hmdSnWY/view?usp=sharing)

**NMR FIDs** [**Click here**](https://drive.google.com/file/d/1GmCyB8zQd6Z3EIA8ViEJ-UbuZ2mI6HWG/view?usp=sharing)
